# Integrative Analysis Reveals Common and Unique Roles of Tetraspanins in Fibrosis and Emphysema

**DOI:** 10.3389/fgene.2020.585998

**Published:** 2020-12-10

**Authors:** Lokesh P. Tripathi, Mari N. Itoh, Yoshito Takeda, Kazuyuki Tsujino, Yasushi Kondo, Atsushi Kumanogoh, Kenji Mizuguchi

**Affiliations:** ^1^National Institutes of Biomedical Innovation, Health and Nutrition, Ibaraki, Japan; ^2^Artificial Intelligence Center for Health and Biomedical Research (ArCHER), National Institutes of Biomedical Innovation, Health and Nutrition, Ibaraki, Japan; ^3^Department of Respiratory Medicine, Allergy and Rheumatic Diseases, Osaka University Graduate School of Medicine, Suita, Japan; ^4^Research Division, Sumitomo Dainippon Pharma Co., Ltd., Osaka, Japan; ^5^Institute for Protein Research, Osaka University, Suita, Japan

**Keywords:** tetraspanins, COPD, IPF, integrative gene expression analysis, cellular networks, signaling pathways, systems biology, biomarker discovery, disease biology

## Abstract

While both chronic obstructive pulmonary disease (COPD) and idiopathic pulmonary fibrosis (IPF) are multifactorial disorders characterized by distinct clinical and pathological features, their commonalities and differences have not been fully elucidated. We sought to investigate the preventive roles of tetraspanins Cd151 and Cd9 -that are involved in diverse cellular processes in lung pathophysiology- in pulmonary fibrosis and emphysema, respectively, and to obtain a deeper understanding of their underlying molecular mechanisms toward facilitating improved therapeutic outcomes. Using an integrative approach, we examined the transcriptomic changes in the lungs of Cd151- and Cd9-deficient mice using functional-enrichment-analysis, pathway-perturbation-analysis and protein-protein-interaction (PPI) network analysis. Circadian-rhythm, extracellular-matrix (ECM), cell-adhesion and inflammatory responses and associated factors were prominently influenced by Cd151-deletion. Conversely, cellular-junctions, focal-adhesion, vascular-remodeling, and TNF-signaling were deeply impacted by Cd9-deletion. We also highlighted a “common core” of factors and signaling cascades that underlie the functions of both Cd151 and Cd9 in lung pathology. Circadian dysregulation following Cd151-deletion seemingly facilitated progressive fibrotic lung phenotype. Conversely, TGF-β signaling attenuation and TNF-signaling activation emerged as potentially novel functionaries of Cd9-deletion-induced emphysema. Our findings offer promising avenues for developing novel therapeutic treatments for pulmonary fibrosis and emphysema.

## Introduction

COPD is a progressive lung disease that is characterized by the exposure to a noxious agent (such as cigarette smoke) resulting in airflow limitation that is not fully reversible. A subset of COPD patients sustain the destruction of lung elastin and other ECM proteins, apoptosis of alveolar cells, or repair failure that leads to airspace enlargement, a characteristic of emphysema (Vogelmeier et al., 2017). IPF, on the other hand, is characterized by interstitial pneumonia, uncontrolled and progressive ECM-deposition, and abnormal alveolar remodeling (Raghu et al., 2011). Combined pulmonary fibrosis and emphysema (CPFE) is recognized as a clinical syndrome that is defined by the co-existence of emphysema and parenchymal fibrosis in the patient (Cottin et al., 2005). While CPFE patients have distinct clinical features and possibly different outcomes, their pathogenesis remains unclear.

Tetraspanins are integral membrane proteins with four transmembrane helices that participate in varied cellular and physiological processes (Charrin et al., 2014; Termini and Gillette, 2017). Some tetraspanins such as Cd151 are widely expressed in a variety of cancer types and positively regulate tumor progression (Kumari et al., 2015). Cd9, in contrast, negatively regulates tumor progression.

We had previously demonstrated that Cd151 is necessary for the integrity of the alveolar epithelial cells (AECs); Cd151 deletion had resulted in the activation of phosphorylated Smad2 and EMT-like changes that may potentially contribute to the development of pulmonary fibrosis in the mouse lung. Indeed, it was further observed that Cd151-knockout mice (Cd151KO) had spontaneously developed age-related pulmonary fibrosis (Tsujino et al., 2012). Conversely, Cd9-deletion exacerbated lipopolysaccharide (LPS) induced lung inflammation that was characterized by macrophage infiltration and the overproduction of TNF-α and matrix metalloproteinsases (MMPs). Moreover, Cd9-deficient mice (Cd9KO) were prone to progressive emphysema, a major pathological component of COPD (Takeda et al., 2008; Suzuki et al., 2009). We further demonstrated that Cd9/Cd81 double-knockout mice spontaneously developed pulmonary emphysema (Jin et al., 2018) and that Cd9 likely performs protective roles in lung inflammation and emphysema.

**FIGURE 1:**
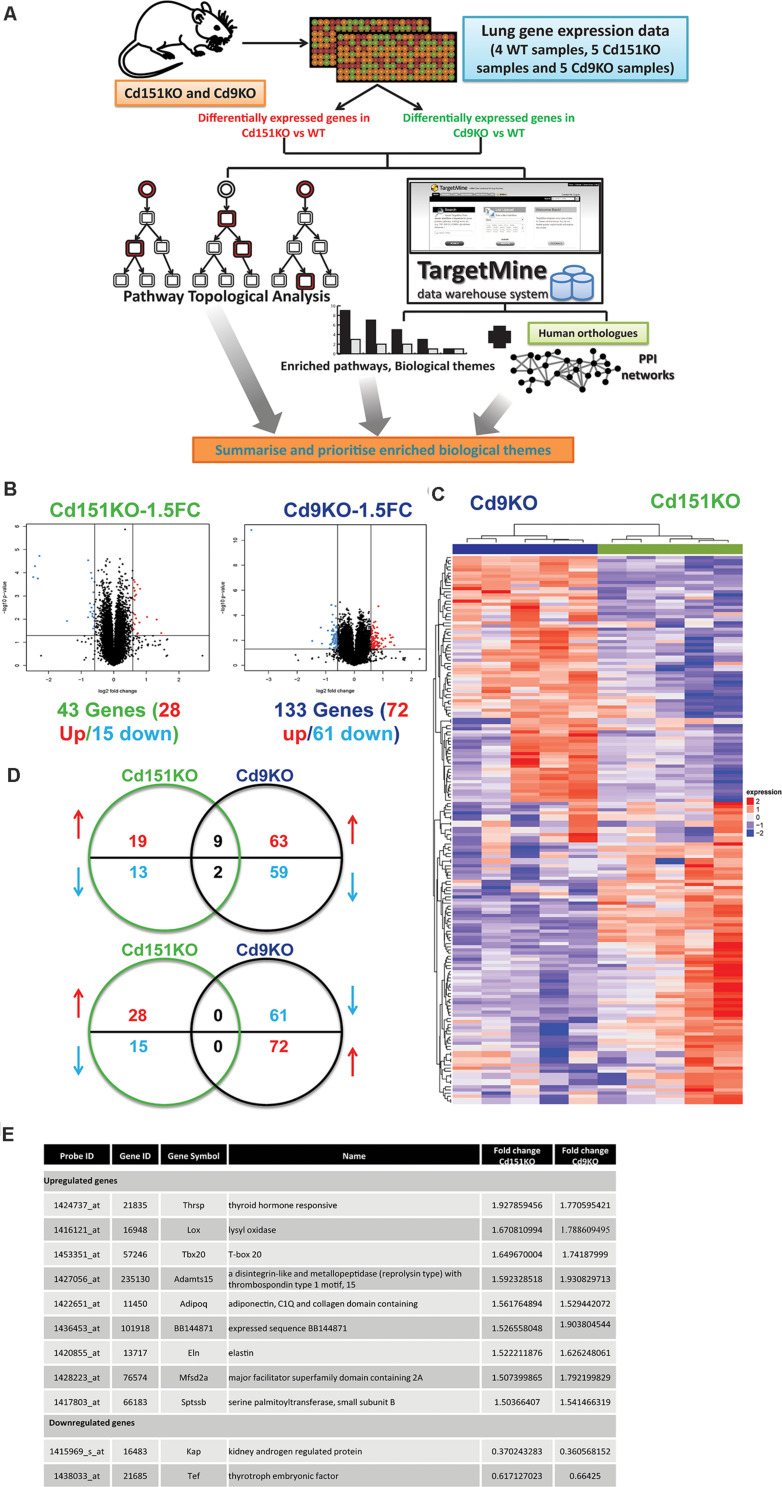
**(A)** A multi-omics framework to investigate the transcriptomic changes underlying the deletion of Cd151 and Cd9. **(B)** Volcano plots for Cd151KO and Cd9KO sDEGs (1.5FC). Upregulated probes (genes) are highlighted in red, whereas the downregulated probes (genes) are highlighted in blue. **(C)** A heatmap of the significantly fluctuated gene expression levels in Cd151KO (green) and Cd9KO (deep blue). Red indicates relative higher expression, blue indicates relative lower expression; values in each row are normalized gene expression. Both rows and columns were hierarchically clustered and the results were illustrated as dendrograms. **(D)** Cd151KO (green) and Cd9KO (deep blue) sDEGs show very little overlap in either upregulated genes (red upward arrow) or downregulated genes (blue downward arrow). **(E)** The similarly affected genes in Cd151KO and Cd9KO indicating convergent underlying mechanisms.

Here, we have employed an integrative bioinformatics approach to investigate the transcriptomic changes in the mouse lung tissue underlying the deletions of Cd151 and Cd9 to (a) gain a perspective of a corresponding phenomenon in context of the human lung and; (b) to better understand the preventive roles of Cd151 and Cd9 in pulmonary fibrosis and emphysema, respectively (Figure 1A). First, we examined the significantly differentially expressed genes (sDEGs) in Cd151KO and Cd9KO lungs for enriched biological themes to highlight key cellular processes that were dysregulated by Cd151- and Cd9-deletions. Next, we investigated the perturbative impacts of these dysregulated gene expressions on cellular signaling pathways. We finally investigated these transcriptomic changes in the context of human orthologous PPI networks (PPINs) to highlight affected signaling cascades and probable crosslinks between them.

Our analysis has led to a deeper understanding of the molecular mechanisms underlying lung fibrosis and emphysema and provided promising avenues for improved therapeutic applications. We describe our analysis and discuss our observations below.

## Materials and Methods

### Animal Models

Cd151KO and Cd9KO were generated as before (Tsujino et al., 2012). All animal experiments were approved by the Animal Care and Use Committee of Osaka University, and all of the animal procedures were performed in accordance with the Osaka University guidelines on animal care.

### Sample Preparation and Microarray Gene Expression Analysis

Lungs removed from mice (WT (*n* = 4), CD151 KO (*n* = 5), and CD9 KO (*n* = 5), at 20 weeks of age) were immersed in RNA-Later solution (Invitrogen) and stored at −80°C until RNA extraction. The lungs were transferred into QIAzol lysis reagent (Qiagen) and disrupted and homogenized using a TissueLyser homogenizer (Qiagen). Total RNA was extracted from the lysates using the RNeasy mini kits (Qiagen) according to the manufacturer’s instructions.

Gene expression profiles were obtained from 100 ng total RNA per sample using GeneChip Mouse Genome 430 2.0 Arrays and GeneChip 3’-IVT Express Kit (Affymetrix). The signal intensities derived from probe sets were normalized among samples using the Microarray Suite (MAS) 5.0 algorithm implemented in the Expression Console software (Affymetrix), where signal intensities of all probe sets were linearly scaled as their trimmed mean values to be 500. QC metrics reported by the software were confirmed to be within normal ranges.

The differences between the two groups were evaluated using two-sided Student’s *t*-test and *p* ≤ 0.05 were considered statistically significant. Probesets were judged to be differentially expressed if the Fold change (FC) ≥ 1.5 (upregulated) or FC ≤ 0.6667 (downregulated), in Cd151KO/Cd9KO vs. WT. These probesets were transformed into sDEG-sets for subsequent analyses; for genes with multiple probesets, the highest FC values were selected.

### Gene Set Functional Enrichment (GSFE) Analysis

Cd151KO and Cd9KO sDEGs were examined with TargetMine data analysis platform^1^ (Chen et al., 2011, 2016). The enrichment of specific KEGG pathways (Kanehisa et al., 2016), Reactome pathways (Fabregat et al., 2016) and Gene Ontology (GO) associations was estimated using Fisher’s exact test (Gene Ontology Consortium, 2015). Inferred *p*-values were further adjusted for multiple-test-correction to control the false-discovery-rate using the Benjamini-Hochberg procedure (Benjamini et al., 2001), and the annotations/pathways were considered significant if the adjusted *p*-values ≤ 0.05. The enriched associations were visualized as heatmaps with the TargetMine auxiliary toolkit (Chen et al., 2016).

### Co-expression Gene Module Network Analysis

To identify co-expressed genes specifically associated with Cd151KO or Cd9KO, we employed the weighted gene co-expression network analysis (WGCNA) package in R (Langfelder and Horvath, 2008). A unified set of Cd151KO and Cd9KO sDEGs was used for the estimation of Cd151KO or Cd9KO-specific gene modules. The blockwise-Module function was used with the default parameters and power = 7; deepSplit = 2. The selected genes were further examined for functional enrichment analysis.

### Pathway-Perturbation Analysis

We employed Signaling Pathway Impact Analysis (SPIA) package in R (Tarca et al., 2009) to analyse KEGG signalling pathways in mouse. SPIA combines data from gene expression measurements with the classical pathway enrichment (overrepresentation) analysis and the actual perturbation of a given pathway under specific conditions. SPIA achieves this by estimating a global pathway significance *p*-value (pG) that combines pathway enrichment (pNDE) and perturbation *p*-values (pPERT), thereby providing a robust assessment of the impact of differential gene expression in the biological processes under study.

### Construction of Orthologous sDEGs PPINs

PPI networks for Cd151KO and Cd9KO sDEGs were constructed using TargetMine (see Supplementary Material for details). Network components were visualized with Cytoscape (Cline et al., 2007) and network *hubs* and *bottlenecks* (Chen et al., 2016) were assigned using TargetMine.

## Results

### Cd151KO and Cd9KO Show Distinct Transcriptomic Profiles

Forty three sDEGs were upregulated (28 genes) or downregulated (15 genes) in Cd151KO. Likewise, 133 (72 upregulated and 61 downregulated) sDEGs were identified in Cd9KO (Figure 1B and Supplementary Tables S1A,B). Cd151KO and Cd9KO displayed dissimilar transcriptomic profiles (Figure 1C). Only nine genes (*Adamts15*, *Adipoq*, *BB144871*, *Eln*, *Lox*, *Mfsd2a*, *Sptssb*, *Tbx20*, and *Thrsp*) were upregulated and two genes (*Kap*, *Tef*) were downregulated in both Cd151KO and Cd9KO (Figures 1D,E and Supplementary Table S1).

### GSFE Analysis of Cd151KO and Cd9KO sDEGs

First, we investigated the Cd151KO and Cd9KO sDEGs for enriched biological themes.

#### Cd151KO sDEGs Were Enriched in Circadian Rhythm, ECM, and Collagen Deposition Pathways

**FIGURE 2:**
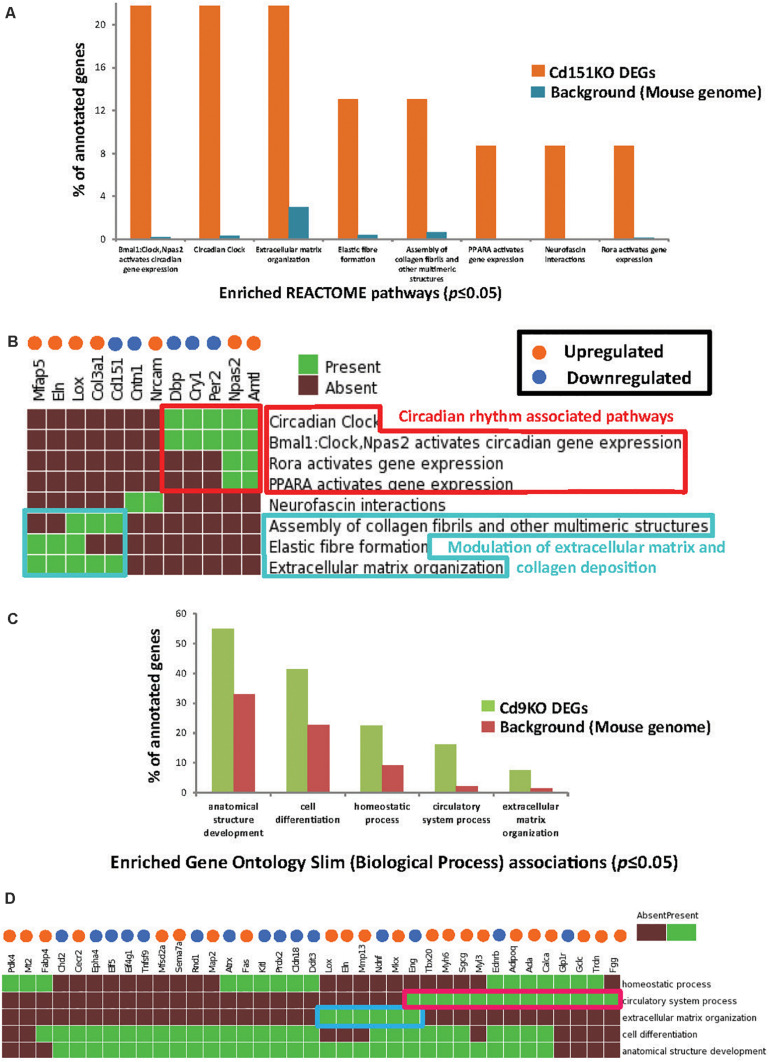
**(A)** Enriched Reactome pathways (*p* ≤ 0.05) identified in Cd151KO 1.5FC sDEGs. For clarity, only select Reactome pathways are displayed. **(B)** Cd151KO sDEGs were distinctly enriched in Reactome pathways associated with circadian rhythm (red box) and extracellular matrix (azure box). **(C)** Enriched GO Slim biological process (BP) terms (*p* ≤ 0.05) identified in Cd9KO 1.5FC sDEGs. **(D)** Cd9KO sDEGs were significantly associated with enriched GO Slim BP terms “circulatory system process” (red box) and “extracellular matrix” (blue box); for clarity, only a subset of the heatmap is highlighted here. In **(B,D)**, the upregulated genes are indicated by orange spheres and downregulated genes are indicated by blue spheres.

GSFE (Figure 2A and Supplementary Table S2) with Cd151KO sDEGs revealed that 12 sDEGs (seven upregulated and five downregulated; Figure 2B) were mapped to pathways associated with circadian rhythm, regulation of ECM and collagen deposition (Figures 2A,B, Supplementary Table S2, and Supplementary Material).

Specifically, circadian clock-associated genes *Cry1* (0.58FC), *Per2* (0.65FC), and *Per3* (0.66FC) were downregulated in Cd151KO, but not in Cd9KO (*Cry1* was marginally upregulated 1.17-fold in Cd9KO). Conversely, circadian regulator *Arntl* (Bmal1) was elevated 2.4-fold in Cd151KO (1.01FC in Cd9KO) (Supplementary Table S1A). Additionally, *Lox* (lysysl oxidase), an ECM modifying enzyme, *Col3a1* (collagen, type III, alpha I), *Mfap5* (microfibrillar associated protein 5) and *Eln* (elastin), an ECM protein and a key component of lung tissues, were upregulated 1. 67-, 1. 56-, 1. 53-, and 1.52-fold, respectively, in Cd151KO (Supplementary Table S1 and Figure 2B).

The WGCNA algorithm (Langfelder and Horvath, 2008) (Supplementary Material) analysis also identified 31 Cd151KO-specific sDEGs that included key “Circadian rhythm” components *Arntl*, *Cry1*, *Npas2*, *Per2*, and *Per3*.

#### Cd9KO sDEGs Were Significantly Associated With ECM

Biological enrichment analysis (Figure 2C and Supplementary Table S2) of the Cd9KO sDEGs revealed that 52 sDEGs (27 upregulated and 25 downregulated; Figure 2D) were mapped to five GO (Slim) Biological Process terms (*p* ≤ 0.05): “homeostasis,” “circulatory system process,” “extracellular matrix organization,” “cell differentiation” and “anatomical structure development” (Supplementary Table S2).

Cd9-deletion results in aberrations in cell matrix (Suzuki et al., 2009). *Lox*, Mkx, *Eln*, and *Mmp13*, a matrix metalloproteinsase, were upregulated 1. 78-, 1. 67-, 1. 62-, and 1.55-fold, respectively; conversely, *Eng* (Endoglin), a glycoprotein, and *Ndnf*, a neutrotrophic factor, were downregulated 0.66- and 0.55-fold, respectively, in Cd9KO (Supplementary Table S1B).

Cd9-deletion is also associated with diminished angiogenetic effects (Iwasaki et al., 2013). Accordingly, many factors dysregulated in Cd9KO were known to be dysregulated in airway obstruction, emphysema and COPD (Miller et al., 2009; Llinas et al., 2011; Brightling, 2013). Among these factors, *Adipoq* (Adiponectin), *FggFgg* (fibrinogen gamma chain), *Gclc* (glutamate-cysteine ligase, catalytic subunit), and *Calca* (Calcitonin) were upregulated 1. 52-, 1. 64-, 1. 63-, and 1.5-fold, respectively, in Cd9KO (Supplementary Table S1B and Figure 2D). Conversely, *Glp1r* (glucagon-like peptide 1 receptor) and *Ednrb* (endothelin receptor type B) were downregulated 0.66- and 0.65-fold, respectively, in Cd9KO (Supplementary Table S1B).

### Signaling Pathway Perturbations in Cd151KO and Cd9KO

Next, we investigated the perturbative effects of Cd151- and Cd9-deletions on signaling pathways using SPIA (Supplementary Material, Figures 3, 4, and Supplementary Figures S1, S3).

**FIGURE 3:**
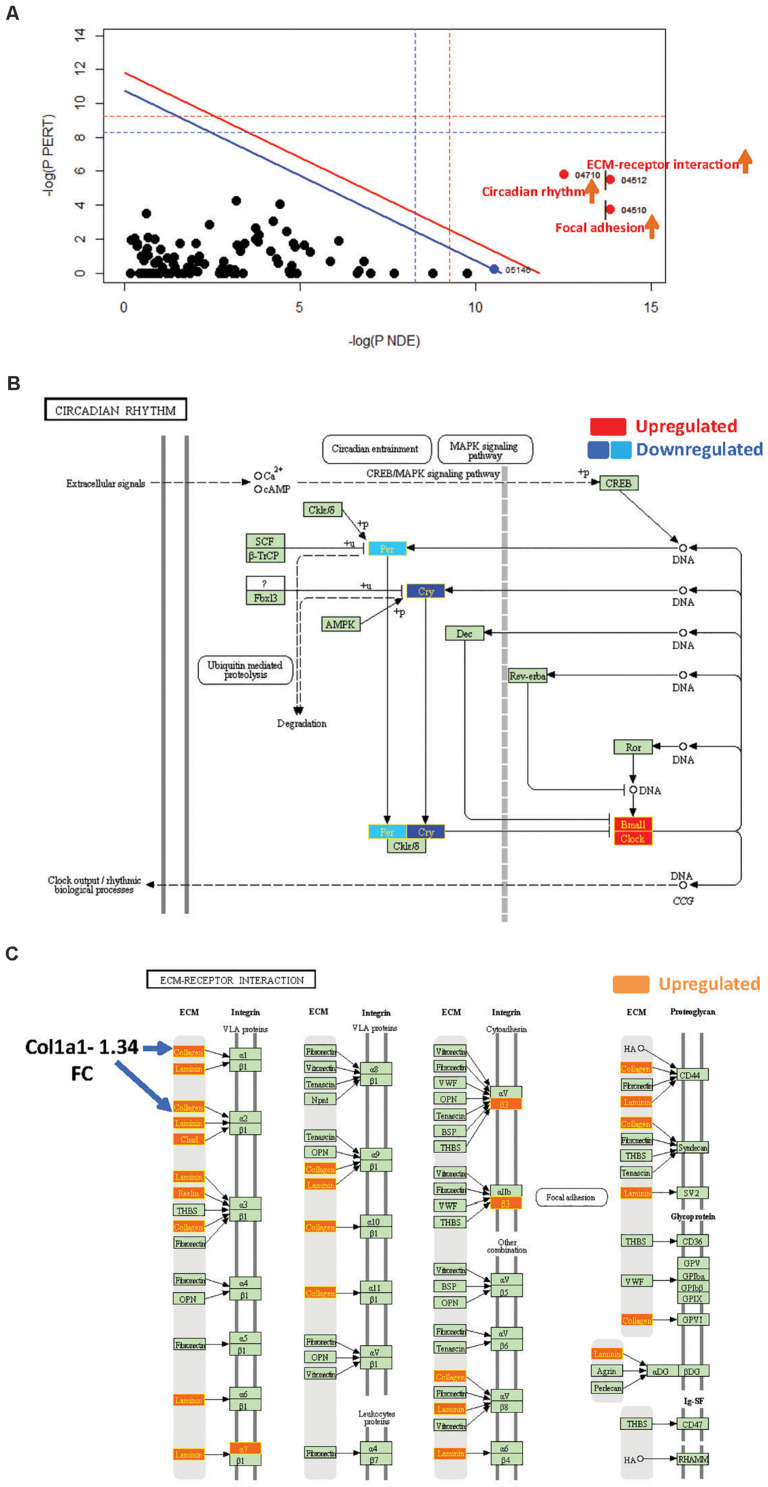
SPIA identified pathway perturbations in Cd151KO. **(A)** SPIA two-way evidence plots for Cd151 1.2FC sDEGs. Significantly perturbed pathways are highlighted in red. “Activated” pathways are tagged with a red upward arrow. **(B)** KEGG pathway hsa04710: “Circadian rhythm” was markedly activated in Cd151KO 1.2FC sDEGs. Upregulated genes are highlighted in red and the downregulated genes are highlighted in dark blue (Cry) and light blue (Per), rectangles, respectively, with gene labels within highlighted in yellow. **(C)** KEGG pathway hsa04512: “ECM-receptor interaction” was activated in Cd151KO Upregulated genes are highlighted in Orange rectangles; their corresponding gene labels within the rectangles are highlighted in yellow.

**FIGURE 4:**
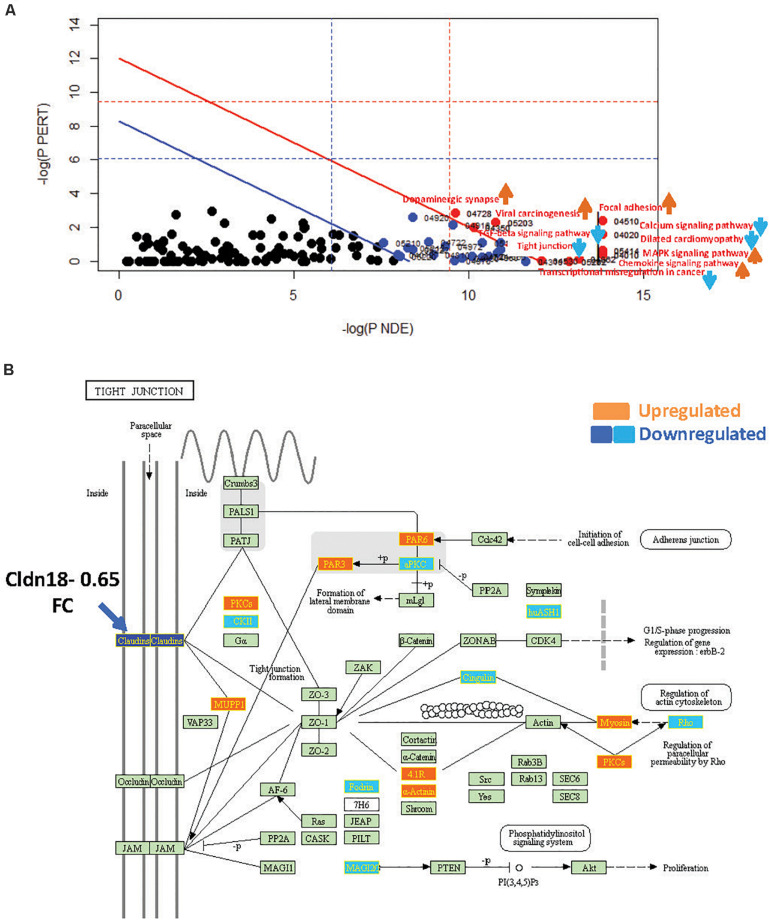
SPIA identified pathway perturbations in Cd9KO. **(A)** SPIA two-way evidence plots for Cd9KO 1.2FC sDEGs. Significantly perturbed pathways are highlighted in red. “Activated” pathways are tagged with a red upward arrow and “Inhibited” pathways are tagged with a blue downward arrow. **(B)** KEGG pathway hsa04530: “Tight junction” was identified to be inhibited in Cd9KO. Upregulated genes are highlighted in orange and the downregulated genes are highlighted in dark blue (Claudins) and light blue (others), rectangles, respectively; their corresponding gene labels within the rectangles are highlighted in yellow.

#### Cd151-Deletion Activated Circadian Rhythm, ECM, and Focal Adhesion Pathways

SPIA revealed that “Circadian rhythm” was “Activated” in Cd151KO (pGFWER = 1.55 × 10^–9^) (Supplementary Figure S3 and Supplementary Table S3). Circadian rhythm is an internal biological clock that modulates the daily physiology and behavior of organisms. This 24-h coordination is achieved by intracellular transcription feedback loops; E-box-driven period (PER) and cryptochrome (CRY) proteins heterodimerize and impede the transcriptional activator complex CLOCK/BMAL1 to repress their own transcription (Albrecht, 2012; Peek et al., 2015). SPIA suggested an activation of “Circadian rhythm” pathway (and consequently increased clock output) due to downregulation of *Cry1* and *Per2* and upregulation of the transcriptional activators *Arntl*1 and *Npas2* (Garavoglia et al., 1993; Haque et al., 2010; Landgraf et al., 2016) following Cd151-deletion (Figure 3B).

SPIA also highlighted that “ECM-receptor interaction” and “Focal adhesion” pathways were “Activated” (Figure 3A and Supplementary Table S3) in Cd151KO (with 1.2FC sDEGs; Supplementary Material). Therefore, Cd151-deletion resulted in increased collagen- and ECM-deposition via upregulation of *Col3a1* (1.56FC) and *Lamc2* (laminin, gamma; 1.23FC). The increased ECM-deposition induced a loosening of cellular adhesions chiefly via upregulation of cell surface proteins including *Intga7* (integrin alpha 7; 1.25FC) and *Intgb3* (integrin beta 3; 1.26FC). The activation of “ECM-receptor interaction” and “Focal adhesion” pathways manifested in the upregulation of cell proliferation factor *Cycd* (cyclin D; 1.23FC) and in downregulation of apoptosis-inhibitor *Xiap* (X-linked inhibitor of apoptosis; 0.8FC) (Figure 3C and Supplementary Tables S1A, S3).

#### Cd9-Deletion Perturbed ECM, Cellular Junction, and Inflammatory Immune Response Pathways

SPIA with Cd9KO 1.2FC sDEGs (Supplementary Figure S1) highlighted ten significantly impacted pathways (five “Activated” and five “Inhibited”; Supplementary Material) in Cd9KO (Figure 4A and Supplementary Table S3).

Cd9-deletion significantly impacted pro-inflammatory signaling, chiefly via the activations of “MAPK signaling pathway” (mmu04010; pGFWER = 0.000834) and “Chemokine signaling pathway” (mmu04062; pGFWER = 0.00315), which are consistent with increased lung inflammation and pro-inflammatory cytokines in Cd9KO (Suzuki et al., 2009).

Cd9-deletion also significantly “Inhibited” “Tight Junction” pathway, chiefly because *Cldn18* (Claudin18), an integral membrane protein, was decreased 0.65-fold (Supplementary Table S1B); Cldn18 was implicated as a key factor in lung barrier and alveolar junction functions and in lung inflammation (Schlingmann et al., 2015; Shimobaba et al., 2016). An examination of the “Tight Junction” pathway map (Figure 4B) suggested that a decrease in Cldn18 function may likely impede tight junction formation, thereby affecting multiple downstream processes including cellular migration and motility (Figure 4B).

“TGF-beta signaling pathway” (mmu04350; pGFWER = 0.009) was shown to be “Inhibited” in Cd9KO (Supplementary Table S3). Furthermore, *Decorin*, a Tgfb antagonist that sequesters Tgfb in the ECM thereby preventing it from interacting with its cell surface receptors (Jarvinen and Prince, 2015), was upregulated 1.35-fold in Cd9KO.

### Network Analysis of Cd151KO and Cd9KO sDEGs

To circumvent the limited repertoire of mouse HCDPs (see Supplementary Methods), we retrieved the human orthologs of Cd151KO and Cd9KO sDEGs using TargetMine and inferred the Cd151KO and Cd9KO orthologous sDEGs networks (hereafter Cd151KO-network and Cd9KO-network, respectively) to examine the transcriptomic changes in a network context (Supplementary Figures S4A,B and Supplementary Material). In total, 38 human orthologs of 43 Cd151KO sDEGs (88.3%) and 116 human orthologs of 133 Cd9KO sDEGs (87.2%) were retrieved in this manner (Supplementary Table S4). The Cd151KO- and Cd9KO-networks were comprised of 110 genes, with 168 interactions between them, and of 347 genes, with 683 interactions between them, respectively (Supplementary Figure S4B and Supplementary Tables S5–S7).

#### Cross-Talks Across Circadian Clock and Cell Cycle, Aeging, and Inflammation-Related Modules in Cd151KO-Network

**FIGURE 5:**
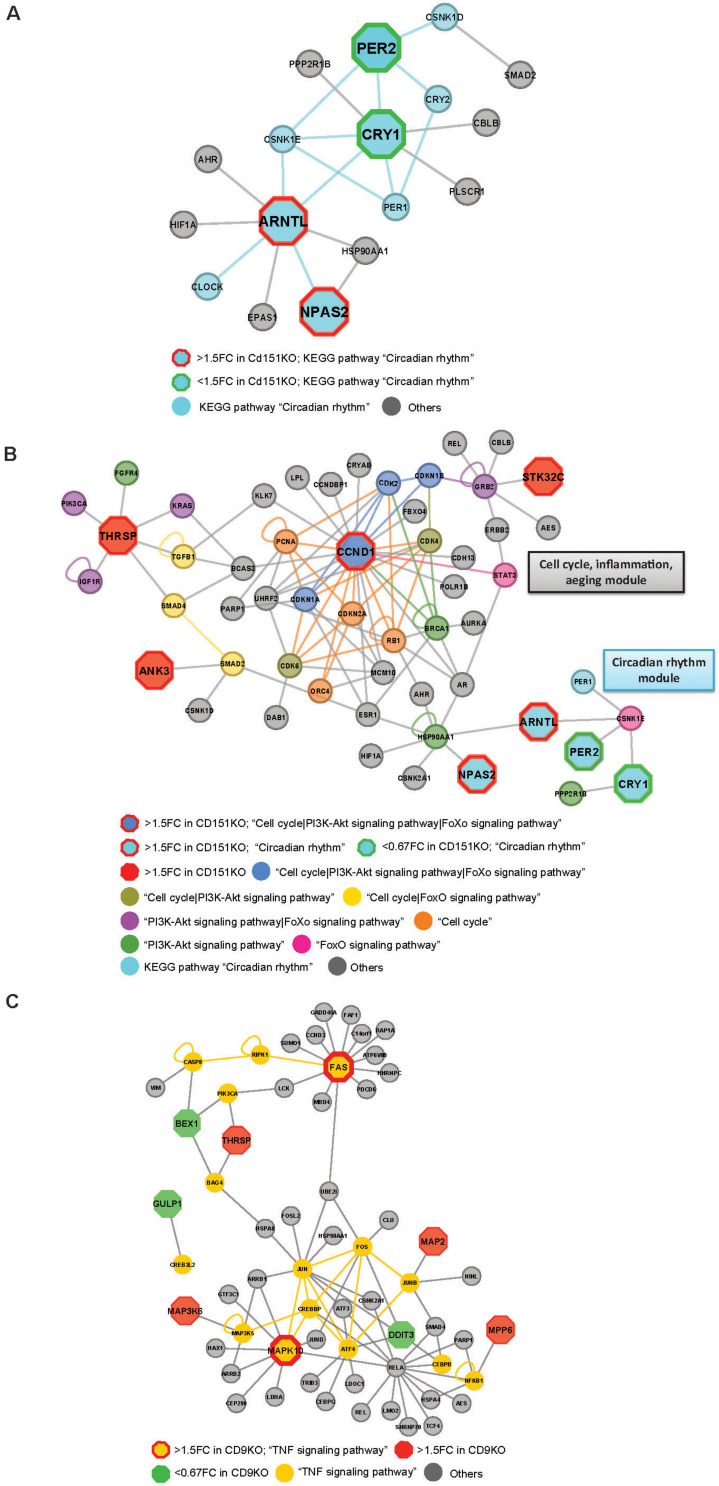
**(A)** Cd151KO 1.5FC sDEGs orthologs and their interacting partners associated with Enriched KEGG pathway hsa04710: “Circadian rhythm.” **(B)** Cd151KO 1.5FC sDEGs orthologs and their interacting partners associated with Enriched KEGG pathways hsa04110: “Cell cycle,” hsa04710: “Circadian rhythm,” hsa04068: “FoxO signaling pathway,” and hsa04151: “PI3K-Akt signaling pathway.” **(C)** Cd9KO 1.5FC sDEGs orthologs and their interacting partners associated with enriched KEGG pathway hsa04668: “TNF signaling pathway.”

Cd151KO-network analysis revealed key interactions of circadian clock factors that were likely to be impacted by Cd151-deletion; PER2, the human ortholog of Per2 (downregulated in Cd151KO) interacted with CRY2 (Figure 5A), the human ortholog of Cry2 (unchanged in Cd151KO).

Network representations also suggested extensive cross-talks between Cell cycle, PI3-Akt signaling and aeging-related FoxO signaling pathways that were likely to be impacted by Cd151-deletion. *Ccnd1* (CyclinD1), a cell cycle regulator was upregulated 1.60-fold in Cd151KO (Supplementary Table S1). Ccnd1 human ortholog, CCND1 and its interactors CDK2, CDKN1A and CDKN1B in the Cd151KO-network were mapped to KEGG pathways “Cell cycle,” “PI3K-Akt signaling pathway” and “FoxO signaling pathway”; two interactors (CDK4, CDK6) were mapped only to “Cell cycle” and “PI3K-Akt signaling pathway”; four (CDKN2A, PCNA, ORC4 and RB1) were mapped only to “Cell cycle”; BRCA1 was mapped to “PI3K-Akt signaling pathway” and STAT3 was mapped to “FoxO signaling pathway.” Thus, CCND1, which was classified as a bottleneck-hub (Supplementary Table S7) appeared to mediate cross-talks across multiple pathways via multiple PPIs (Figure 5B).

*Thrsp* (thyroid hormone responsive), a lipid metabolism regulatory factor, was upregulated 1.92-fold in Cd151KO (Figure 1E and Supplementary Table S1). Although not associated with enriched pathways, THRSP was identified as a hub (Supplementary Table S7); three THRSP interacting partners, IGF1R, KRAS and PIK3CA, were mapped to enriched KEGG pathways “PI3K-Akt signaling pathway” and “FoxO signaling pathway”; two interacting partners, SMAD4 and TGFB1 were mapped to “Cell cycle” and “FoxO signaling pathway” and one interactor FGFR4 was mapped to “Cell cycle” and “PI3K-Akt signaling pathway” (Figure 5B).

HSP90AA1, a cell cycle and transcriptional regulatory chaperone, (identified as a bottleneck) was mapped to the enriched “PI3K-Akt signaling pathway” and was the only factor that interacted with both ARNTL and NPAS2 in the “Circadian rhythm” module (Figures 5A,B). Although *Hsp90aa1* expression was unchanged in Cd151KO vs. WT, HSP90AA1 formed a vital link between the “Cell cycle| PI3-Akt signaling| aeging-related FoxO signaling” module and the “Circadian rhythm” module (Figures 5A,B).

#### PPIN Analysis Highlighted a TNF-Signaling Mediated Pro-Apoptotic Signaling Related Module in Cd9KO

Fas cell surface death receptor (*Fas*) (1.5FC) and *Mapk10* (1.55FC) were elevated in Cd9KO (Supplementary Table S1). In Cd9KO-network, FAS, of the TNF-receptor superfamily that functions in extrinsic apoptosis pathway, was tagged as a bottleneck-hub (Supplementary Table S7), while MAPK10, a pro-apoptotic factor, known to be deleted in small-cell-lung-cancer (Kim et al., 2006), was identified as a bottleneck (Supplementary Table S7). FAS and MAPK10 were mapped to the enriched “TNF signaling pathway” (*p* = 2.8 × 10^–6^) (Supplementary Figure S5B and Supplementary Tables S8A,B) and emerged as key components of the inferred TNF signaling network (Figure 5C).

## Discussion

By analyzing transcriptomic data from transgenic mice deficient in Cd151 (an *in vivo* model of IPF) or Cd9 (an *in vivo* model of emphysema), we obtained deeper insights into their functions in lung pathology. Our observations suggested that Cd151 and Cd9 modulate largely different signaling processes that have probably contributed to their contrasting roles in pulmonary pathophysiology. Specifically, Cd151-deletion resulted in elevated expression of genes involved in collagen deposition and ECM formation and dysregulation of genes associated with the circadian rhythm. Conversely, Cd9-deletion impacted genes associated with vascular remodeling and cell adhesion.

Circadian rhythm emerged as the premier process that was significantly impacted by Cd151-deletion. Circadian clock components *Cry1*, *Per2*, and *Per3* were downregulated and the circadian regulators *Arntl* (Bmal1) and *Npas2* were elevated in Cd151KO that manifested as the “Activation” of the “Circadian rhythm” pathway. Circadian clock dysfunctions are verifiably linked with a decline in lung function in chronic airway diseases, COPD and asthma (Chen et al., 2010; Pekovic-Vaughan et al., 2014; Sundar et al., 2015; Yao et al., 2015). Previously we had noted fibrotic changes in the lung, kidney and liver in Cd151KO, thereby implicating Cd151 in fibrosis in all the three organs (Tsujino et al., 2012). Our current analysis suggested that decreased *Per2* expression following Cd151-deletion may be a prime determinant of the age-related pulmonary and multi-organ fibrosis in Cd151KO.

Cd151KO-network analysis highlighted HSP90AA1 as a probable “bridge” connecting the increased output of the circadian clock with the cell cycle, aeging and inflammation-associated factors. Indeed, HSP90AA1 was previously identified as an important crosslink between PPI-network modules representing cell cycle and signal transduction pathways in lung development (Yu et al., 2016). Thus, our analysis has illuminated a novel interplay between Cd151, circadian clock and cell cycle-related processes in maintaining lung homeostasis, the dysregulation of which may have significantly contributed to progressive multi-organ fibrotic phenotypes in Cd151KO. Circadian clock components, therefore, offer attractive targets for IPF therapy. Recently, the small-molecule modulators of circadian clock components have emerged as promising approaches to restore circadian rhythmicity in diseased conditions (Miller and Hirota, 2020). Specifically, a CLOCK-binding small molecule (CLK8) inhibited the CLOCK-BMAL interaction and dimerization and the subsequent translocation of CLOCK into the nucleus, leading to the enhancement of the circadian rhythm (Doruk et al., 2020); CLK8 thus promises to be a useful means in therapeutic approaches seeking to correct dampened circadian rhythm in diseased conditions. Conversely, future studies that may enable the discovery of novel compounds that dampen the enhanced circadian activities, will have immense potential in the therapeutic interventions against pulmonary fibrosis.

Previously, Cd151-deletion resulted in ECM over-production in lung, in aberrant cell migration and a progressive fibrotic phenotype (Tsujino et al., 2012). Here, Cd151-deletion led to an upregulation of ECM- and collagen assembly-associated genes that are linked with disturbances in lung function. Our results, therefore, suggested that Cd151-deletion directly induced increased collagen deposition and ECM-remodeling that may eventually lead to progressive lung fibrosis.

Cd9-deletion is associated with diminished angiogenetic effects (Iwasaki et al., 2013); our observations suggested that these effects were likely to be mediated by dysregulation of circulatory system-associated genes *Adipoq* (Adiponectin), *Fgg* (fibrinogen gamma chain) *Gclc* (glutamate-cysteine ligase, catalytic subunit), *Calca* (Calcitonin) and *Lox*.

Among other examples, *Glp1r* (glucagon-like peptide 1 receptor), which is decreased in smokers with moderate COPD (Llinas et al., 2011), was decreased in Cd9KO. Conversely, Cd9-deletion led to increased *Mmp13* levels that are also elevated in COPD patients (Lee et al., 2009).

Furthermore, Cd9-deletion “Activated” “MAPK signaling pathway” and “Chemokine signaling pathway,” both of which are consistent with increased lung inflammation and elevated pro-inflammatory cytokines in Cd9KO (Suzuki et al., 2009) and are also consistent with phenotypes in emphysema.

“TGF-beta signaling pathway” (mmu04350; pGFWER = 0.009108) was “Inhibited” in Cd9KO. TGF-β signaling is crucial in lung fibrosis and we had demonstrated that Cd151-deletion augmented TGF-β signaling in the development of lung fibrosis (Tsujino et al., 2012). No studies have linked TGF-β signaling with CD9, in the context of emphysema. However, it was established that the cell surface protein EWI-2 negatively regulated TGF-β signaling in melanoma by sequestering CD9 (and CD81) and thereby impeding complex formation between TGFβ receptor 1 (TβR1) and TGFβ receptor 2 (TβR2), leading to diminished TGF-β signaling (Wang and Hemler, 2015). Our analysis suggested that the Cd9-deletion is likely to marginally enhance Decorin, which in turn may impede Tgfb binding with TβR1 and TβR2. Taken together with the observation that *Eng*, a glycoprotein involved with TGF-β signaling, was also downregulated (0.66FC) in Cd9KO (Supplementary Table S1), our results suggested for the first time that the loss of Cd9 activity may negatively impact TGF-β signaling and the subsequent transitions in Cd9KO lungs. The observed decrease in TGF-β signaling in Cd9KO mice exhibiting an emphysema phenotype in our study is consistent with the previous studies that have discussed a decrease in TGF-β signaling in human COPD (Wu et al., 2004; Chilosi et al., 2012).

Previously, the loss of Cd9 function exacerbated LPS-induced lung inflammation, chiefly via enhanced production of TNF-α and matrix metalloproteinases (Suzuki et al., 2009). Interestingly, in cigarette smoke-induced emphysema, impeding of Fas-mediated apoptosis signaling attenuated smoking-induced lung injury in AECs (Kim et al., 2016). Cd9KO-network analysis suggested that Cd9-deletion may possibly trigger downstream TNF-signaling, chiefly via upregulation of *Fas*, leading to Fas receptor-induced death-inducing signaling complex (DISC) assembly, thereby significantly contributing to injury and cell death in AECs.

Despite considerable differences in Cd151KO and Cd9KO phenotypes, we observed overlaps between Cd151KO and Cd9KO sDEGs. The overlapping sDEGs were broadly mapped to ECM (*Adamts15*, *Eln*, *Lox*), Vascular remodeling (*Adipoq*, *Lox*, and *Mfsd2a*), Lipid and hormone metabolism (*Adipoq*, *Sptssb*, *Thrsp*) and Transcription regulation (*Tbx20*, *Tef*, *Thrsp*) (Figure 1C and Supplementary Table S1). The overlaps were not limited to sDEGs alone; for instance “Focal Adhesion” (mmu04510) was “Activated” in Cd151KO and Cd9KO (Supplementary Table S3). Likewise, “Pathways in cancer,” “MAPK signaling pathway,” “PI3K-Akt signaling pathway” and “Adherens junction” were significantly enriched both in the Cd151KO- and Cd9KO-networks. An attractive explanation would be that these genes and pathways represent a “common core” of signaling cascades underlying both Cd151 and Cd9 functions in lung pathology and that this “common core” may also be potentially related with the elusive pathophysiology of CPFE.

## Conclusion

Our analysis has provided novel insights into the contrasting roles of Cd151 and Cd9 in the pathogenesis of IPF and emphysema, respectively. We have also illuminated the genes and pathways that appeared to commonly underlie the functions of both Cd151 and Cd9 in lung pathology. Potentially the therapeutic targeting of dysregulated Cd151- and Cd9-signaling may contribute to improved and more effective therapy for pulmonary diseases.

## Data Availability Statement

The microarray data associated with this study has been deposited in GEO (accession number: GSE159236).

## Ethics Statement

The animal study was reviewed and approved by Animal Care and Use Committee of Osaka University.

## Author Contributions

LT, MI, YT, and KM designed the study, were responsible for overall data analysis and interpretation, and wrote the manuscript. LT, MI, YT, KT, YK, AK, and KM were responsible for data gathering and validation. LT and MI performed the computational data analysis. All authors read and approved the final version of the manuscript.

## Conflict of Interest

YK was employed by the company Sumitomo Dainippon Pharma Co., Ltd. The remaining authors declare that the research was conducted in the absence of any commercial or financial relationships that could be construed as a potential conflict of interest.

## Footnotes

1 http://targetmine.mizuguchilab.org
