# Supplementary material for: Integrative Analysis Reveals Common and Unique Roles of Tetraspanins in Fibrosis and Emphysema
Source: Front Genet. 2020 Dec 10;11:585998. doi: 10.3389/fgene.2020.585998 (PMC7793877; doi:10.3389/fgene.2020.585998)
Supplement: Supplementary file 1 [file Data_Sheet_1.pdf]

Supplementary Material

1 Supplementary Data

Supplementary Methods

Construction of orthologous sDEGs PPI networks

The human orthologues of Cd151KO and Cd9KO sDEGs were retrieved using TargetMine. PPIs for these human orthologues of Cd151KO and Cd9KO sDEGs were then retrieved from TargetMine and were appended to the human orthologues of Cd151KO and Cd9KO sDEGS with initial sets to construct Cd151KO and Cd9KO orthologous sDEGs networks (hereafter referred to as Cd151KO and Cd151KO network, respectively). To minimize biological noise, only HCDPs (High Confidence Direct Physical protein interactions, a high quality subset of binary PPIs in TargetMine) were included in the orthologous sDEGs networks. Furthermore, only those interacting proteins that were judged to be 'sufficiently' expressed [1] in human lung were included in the Cd151KO and Cd9KO orthologous sDEGs networks using the following criteria; they must be tagged with one or more of the 13 identifiers in TargetMine- "a549_cell_line", "airway_basal_cells", "airway_epithelial_cells", "alveolar_macrophages", "bronchial_epithelia", "bronchial_epithelial_cell", "bronchial_epithelial_cells", "bronchial_epithelium", "bronchus", "endobronchial_epithelial_lining_fluid", "fetal_lung", "ib31_bronchial_epithelial_cells" and "lung" and have an orthologue in the mouse genome. The network components were visualized using Cytoscape [2] and the network topological attributes of individual network components (genes/proteins) such as hubs and bottlenecks were retrieved from TargetMine. Briefly, hubs are proteins with a high node 'degree', i.e., those having a large number of PPIs and the ability to influence multiple biological processes via these interactors; bottlenecks are proteins with high 'betweenness', i.e., those representing central points for the flow of signaling information across an interaction network [1].

The enrichment of specific KEGG and Reactome pathways and GO term associations within Cd151KO and Cd9KO orthologous sDEGs networks was estimated as described above and the annotations/pathways were judged to be significant if the adjusted p-value was 0.005 or less.

Supplementary Results

Biological enrichment analysis of Cd151KO sDEGs

Biological enrichment analysis (Figure 2A, Table S2) with Cd151KO sDEGs revealed that 12 sDEGs (seven upregulated and five downregulated; Figure 2B) were mapped to 11 enriched Reactome pathways and one enriched KEGG pathway ($p<=0.05$; Table S2). The enriched Reactome pathways broadly encompassed circadian rhythm and regulation of ECM and collagen deposition (Figure 2A, B). A significant number of Cd151KO sDEGs associated with at least one Reactome pathway (5 out of 23; 21.73%) were mapped to enriched pathways that are associated with the mammalian circadian clock. These included an enriched KEGG pathway mmu04710 "Circadian rhythm" ($p=5.946\times10^{-7}$; *Arntl*, *Cry1*, *Npas2*, *Per2*, Per3) (Table S2) and enriched Reactome pathways, "Circadian Clock" (R-MMU-400253; $p=1.493\times10^{-6}$; *Arntl*, *Cry1*, *Dbp*, *Npas2*, *Per2*; 5 of

23; 21.73%), “Bmal1:Clock,Npas2 activates circadian gene expression” (R-MMU-1368110; p=2.952×10^{-7}; *Arntl*, *Cry1*, *Dbp*, *Npas2*, *Per2*; 5 of 23; 21.73%), “PPARA activates gene expression” (R-MMU-1989781; p=0.01; *Arntl*, *Npas2*; 2 of 23; 8.69%) and “Rora activates gene expression” (R-MMU-1368092; p=0.02; *Arntl*, *Npas2*; 2 of 23; 8.69%) (Figure 2A, B; Table S2).

Additionally, a significant number of Cd151KO sDEGs (5 of 23; 21.73%) were mapped to Reactome pathways associated with ECM, collagen assembly and Neurofascin interactions- “Extracellular matrix organization” (R-MMU-1474244; p=0.036;*Cd151*, *Col3a1*, *Eln*, *Lox*, *Mfap5*; 5 of 23; 21.73%), “Elastic fibre formation” (R-MMU-1566948; p=0.0179; *Eln*, *Lox*, *Mfap5*; 3 of 23; 13.04%) and “Assembly of collagen fibrils and other multimeric” (R-MMU-2022090; p=0.0368; *Cd151*, *Col3a1*, *Lox*; 3 of 23; 13.04%) (Figure 2A, B, Table S2).

Biological enrichment analysis of Cd9KO sDEGs

Our analysis revealed that six Cd9KO sDEGs were associated with enriched GO Slim (BP) term “extracellular matrix” (GO:0030198; p=0.0255; 6 of 80=7.5%) (Figure 2C, D; Table S2). Furthermore, many Cd9KO sDEGs were mapped to GO Slim (BP) terms that are associated with the circulatory system. These terms included “circulatory system process” (GO:0003013; p=2.8×10^{-6}; 13 of 80; 16.25%) (Figure 2C, D; Table S2) and enriched GO (BP) terms “blood circulation” (GO:0008015; p=0.00019) and “circulatory system process” (GO:0003013; p=0.00019) (Table S2).

Signaling pathway perturbations induced by the deletions of Cd151 and Cd9

SPIA performed with Cd151KO and Cd9KO 1.5FC sDEGs, yielded limited insights (Figure S3; see below).We, therefore, relaxed the threshold for DEG selection to 1.2FC (Figure S1) to include a larger representation of sDEGs (Figures 3, 4).

SPIA with Cd151KO sDEGs

SPIA with Cd151KO 1.2-FC sDEGs (420 sDEGs; 211 Up, 209 Down; Figure S1) highlighted three KEGG pathways that were significantly impacted (in this instance ‘Activated’) in Cd151KO: “ECM-receptor interaction” (mmu04512; pGFWER=3.71×10^{-6}), “Focal Adhesion” (mmu04510; pGFWER=1.96×10^{-5}) and “Circadian rhythm” (mmu04710; pGFWER=2.26×10^{-5}) (Figure 3A; Table S3).

SPIA with Cd9KO sDEGs

No significantly impacted pathways were observed in Cd9KO sDEGs. SPIA with Cd9KO 1.2-FC sDEGs (1408; 754 Up; 654 Down; Figure S1) highlighted ten significantly impacted pathways in Cd9KO (Figure 4A; Table S3). These included five pathways that were significantly ‘Activated’: “Focal Adhesion” (mmu04510; pGFWER=7.34×10^{-6}), “MAPK signaling pathway” (mmu04010; pGFWER=0.000834), “Chemokine signaling pathway” (mmu04062; pGFWER=0.00315), “Viral carcinogenesis” (mmu05203; pGFWER=0.003842) and “Dopaminergic synapse” (mmu04728; pGFWER=0.00665) and five pathways that were significantly ‘Inhibited’;: “Dilated cardiomyopathy” (mmu05414; pGFWER=3.07×10^{-5}) “Calcium signaling pathway” (mmu05202; pGFWER=7.9×10^{-5}), “Transcriptional misregulation in cancer” (mmu04310; pGFWER=0.004547), “TGF-beta signaling pathway” (mmu04350; pGFWER=0.009108) and “Tight junction” (mmu04530; pGFWER=0.009225) in Cd9KO (Table S3).

Network analysis of Cd151KO and Cd9KO sDEGs

To circumvent the limited repertoire of mouse HCDPs, we retrieved the human orthologues of Cd151KO and Cd9KO sDEGs using TargetMine and inferred the Cd151KO and Cd9KO orthologous sDEGs networks. In total, 38 human orthologues of 43 Cd151KO sDEGs (88.3%) and 116 human orthologues of 133 Cd9KO sDEGs (87.2%) were retrieved in this manner (Table S4). The Cd151KO and Cd9KO networks were comprised of 110 genes, with 168 interactions between them, and of 347 genes, with 683 interactions between them, respectively (Figure S4B; Tables S5, S6, S7).

Network analysis of Cd151KO sDEGs

The Cd151KO orthologous sDEGs network was comprised of 110 genes, with 168 interactions between them (Figure S5A; Tables S5, S6, S7) that were mapped to one or more of the 35 enriched KEGG pathways ($p \leq 0.005$) (Figure S5A; Tables S8A, B). Among the prominent associations, 10 genes were mapped to “Circadian rhythm” (Figure 5A)($p=2.538\times10^{-11}$); 14 were mapped to “PI3K-Akt signaling pathway” ($p=1.892\times10^{-5}$); 13 were mapped to “Cell cycle” ($p=1.358\times10^{-5}$) and 13 were mapped to “FoxO signaling pathway” ($p=3.256\times10^{-5}$) (Figure 5B; Figure S5A, B; Tables S8A, B), thereby reiterating that the constituents of these signaling processes are significantly impacted by the deletion of Cd151.

Network analysis of Cd9KO sDEGs

The Cd9KO network was comprised of 347 genes, with 683 interactions between them (Figure S4B; Tables S5, S6, S7). These genes were mapped to one or more of the 39 enriched KEGG pathways ($p \leq 0.005$) (Figure S5B; Tables S8A, B). Among the prominent associations, 28 genes (15.64% of genes that were annotated with at least one KEGG pathway) were mapped to “MAPK signaling pathway” ($p=6.3716\times10^{-9}$), 17 (9.49%) to “Focal Adhesion” ($p=1.8\times10^{-4}$) and 15 (8.37%) to “TNF signaling pathway” ($p=2.8\times10^{-6}$) (Figure S5B; Tables S8A, B).

Supplementary References

[1] Y.A. Chen, L.P. Tripathi, and K. Mizuguchi, An integrative data analysis platform for gene set analysis and knowledge discovery in a data warehouse framework. Database : the journal of biological databases and curation 2016 (2016).

[2] M.S. Cline, M. Smoot, E. Cerami, A. Kuchinsky, N. Landys, C. Workman, R. Christmas, I. Avila-Campilo, M. Creech, B. Gross, K. Hanspers, R. Isserlin, R. Kelley, S. Killcoyne, S. Lotia, S. Maere, J. Morris, K. Ono, V. Pavlovic, A.R. Pico, A. Vailaya, P.L. Wang, A. Adler, B.R. Conklin, L. Hood, M. Kuiper, C. Sander, I. Schmulevich, B. Schwikowski, G.J. Warner, T. Ideker, and G.D. Bader, Integration of biological networks and gene expression data using Cytoscape. Nature protocols 2 (2007) 2366-82.

2 Supplementary Figures and Tables

Figure S1: Volcano plots for Cd151KO and Cd9KO 1.2FC sDEGs. Upregulated probes (genes) are highlighted in red, while downregulated probes (genes) are highlighted in blue.

Figure S2: A. Clustering results of WGCNA modules in Cd151KO-Cd9KO combined sDEG set showing module membership in colours. The y axis represents network distance as determined by 1 - topological overlap (TO), where values closer to 0 indicate greater similarity of probe expression profiles across samples. B. Cluster analysis and heatmap of the brown module containing 31 genes. Red indicates relative higher expression, blue indicates relative lower expression.

Figure S3: SPIA two-way evidence plot for Cd151KO 1.5FC sDEGs. KEGG pathway hsa04710: “Circadian rhythm” was ‘Activated’ in Cd151KO 1.5FC sDEGs.

Figure S4: A. Cd151KO and B. Cd9KO; 1.5FC sDEGs orthologous lung tissue filtered HCDP networks.

Figure S5: Enriched KEGG pathway associations ($p \leq 0.005$) for the constituents of the A. Cd151KO and B. Cd9KO; 1.5FC sDEGs orthologous lung tissue filtered HCDP networks.

Table S1: A list of 1.5FC sDEGs in A. Cd151KO and B. Cd9KO.

Table S2: Cd151KO and Cd9KO sDEGs- Enriched biological themes ($p \leq 0.05$).

Table S3: KEGG pathways significantly perturbed Cd151KO and Cd9KO as identified by SPIA.

Table S4: Human orthologues of Cd151KO and Cd9KO sDEGs.

Table S5: Genes in Cd151KO and Cd9KO human orthologous 1.5FC sDEGs Lung Tissue filtered HCDP networks.

Table S6: Cd151KO and Cd9KO human orthologous 1.5FC sDEGs Lung Tissue filtered HCDP networks.

Table S7: Human orthologues of Mouse sDEGs that were identified as Bottlenecks and Hubs in Cd151KO and Cd9KO human orthologous 1.5FC sDEGs Lung Tissue filtered HCDP networks.

Table S8: A. Cd151KO and Cd9KO human orthologous 1.5FC sDEGs Lung Tissue filtered HCDP networks- Enriched KEGG pathway associations ($p \leq 0.005$) B. Cd151KO and Cd9KO human orthologous 1.5FC sDEGs Lung Tissue filtered HCDP networks- List of Gene- Enriched KEGG pathway associations ($p \leq 0.005$).

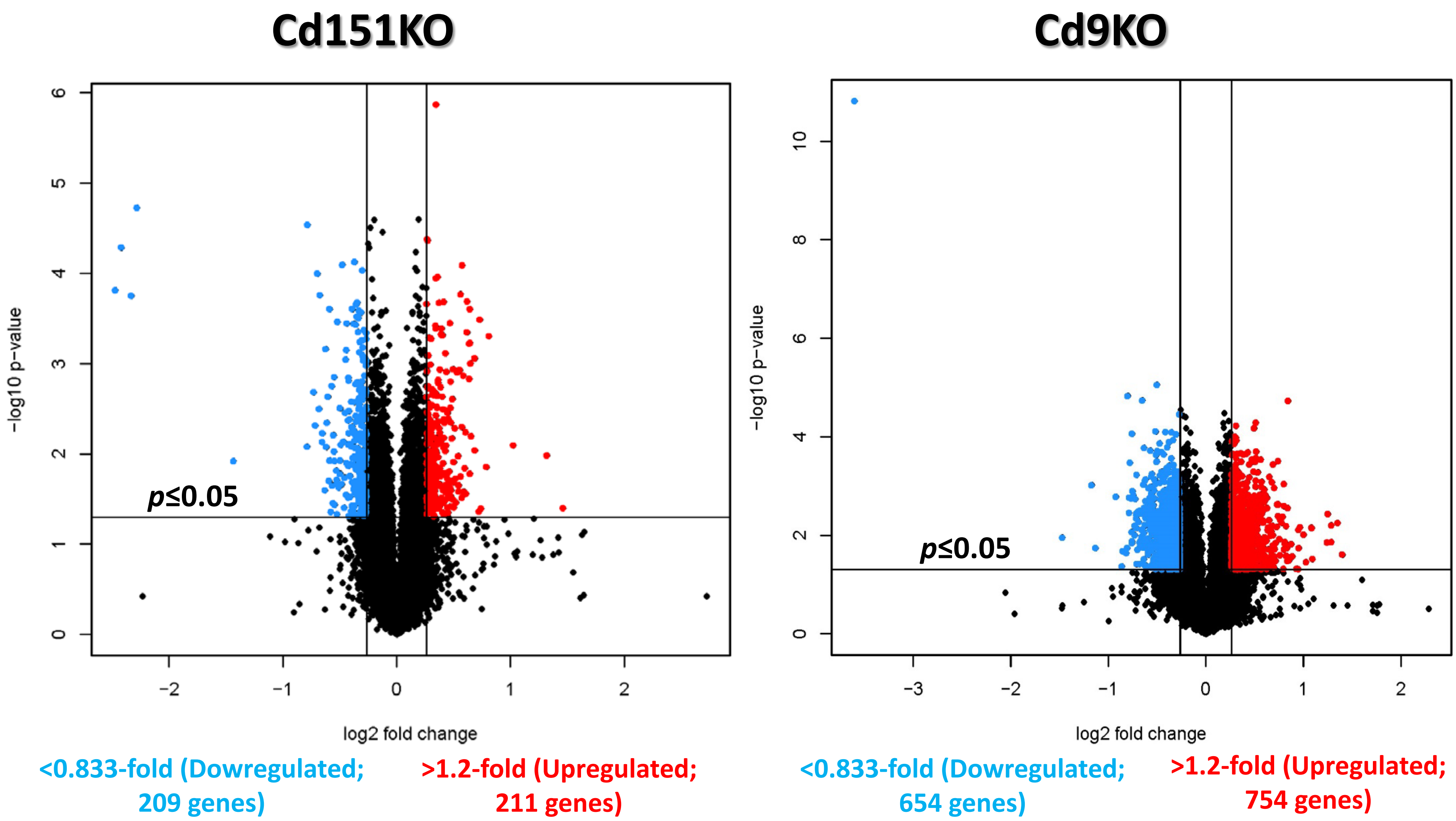

Figure S1: Volcano plots for Cd151KO and Cd9KO 1.2FC sDEGs. Upregulated probes (genes) are highlighted in red, while downregulated probes (genes) are highlighted in blue.

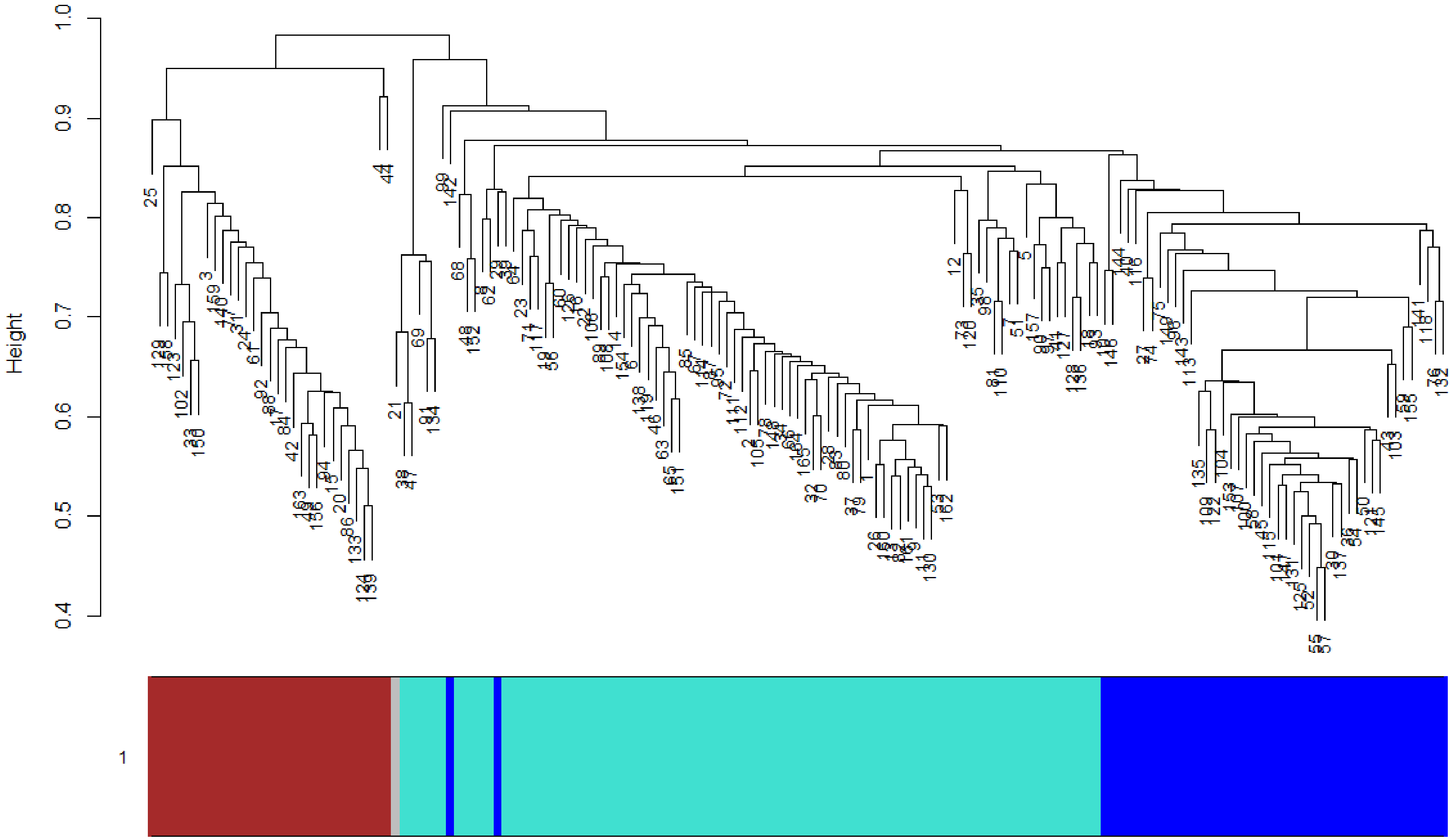

Figure S2A:. Clustering results of WGCNA modules in Cd151KO-Cd9KO combined sDEG set showing module membership in colours. The y axis represents network distance as determined by 1 - topological overlap (TO), where values closer to 0 indicate greater similarity of probe expression profiles across samples.

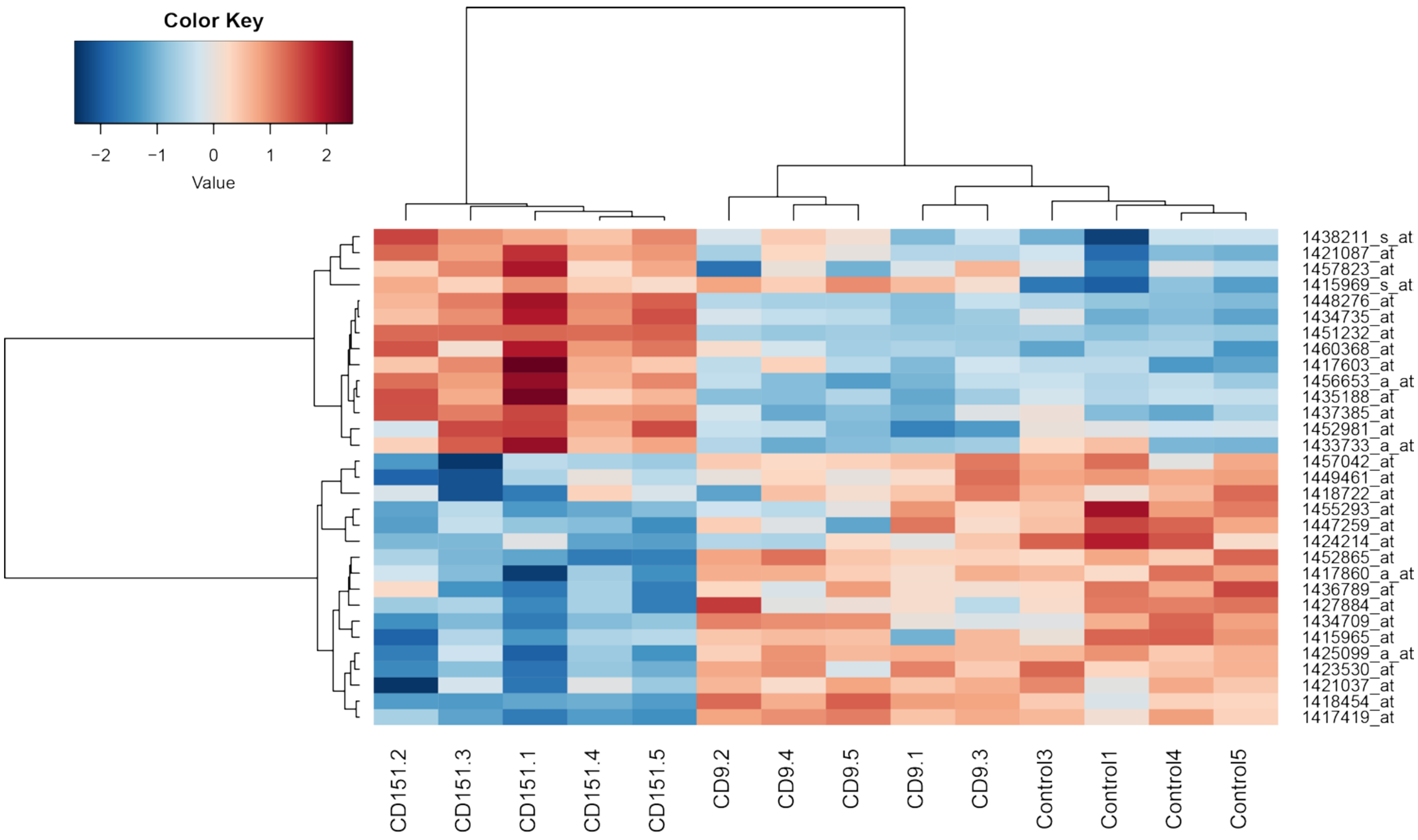

Brown module (31 genes)

Figure S2B: Cluster analysis and heatmap of the brown module containing 31 genes. Red indicates relative higher expression, blue indicates relative lower expression.

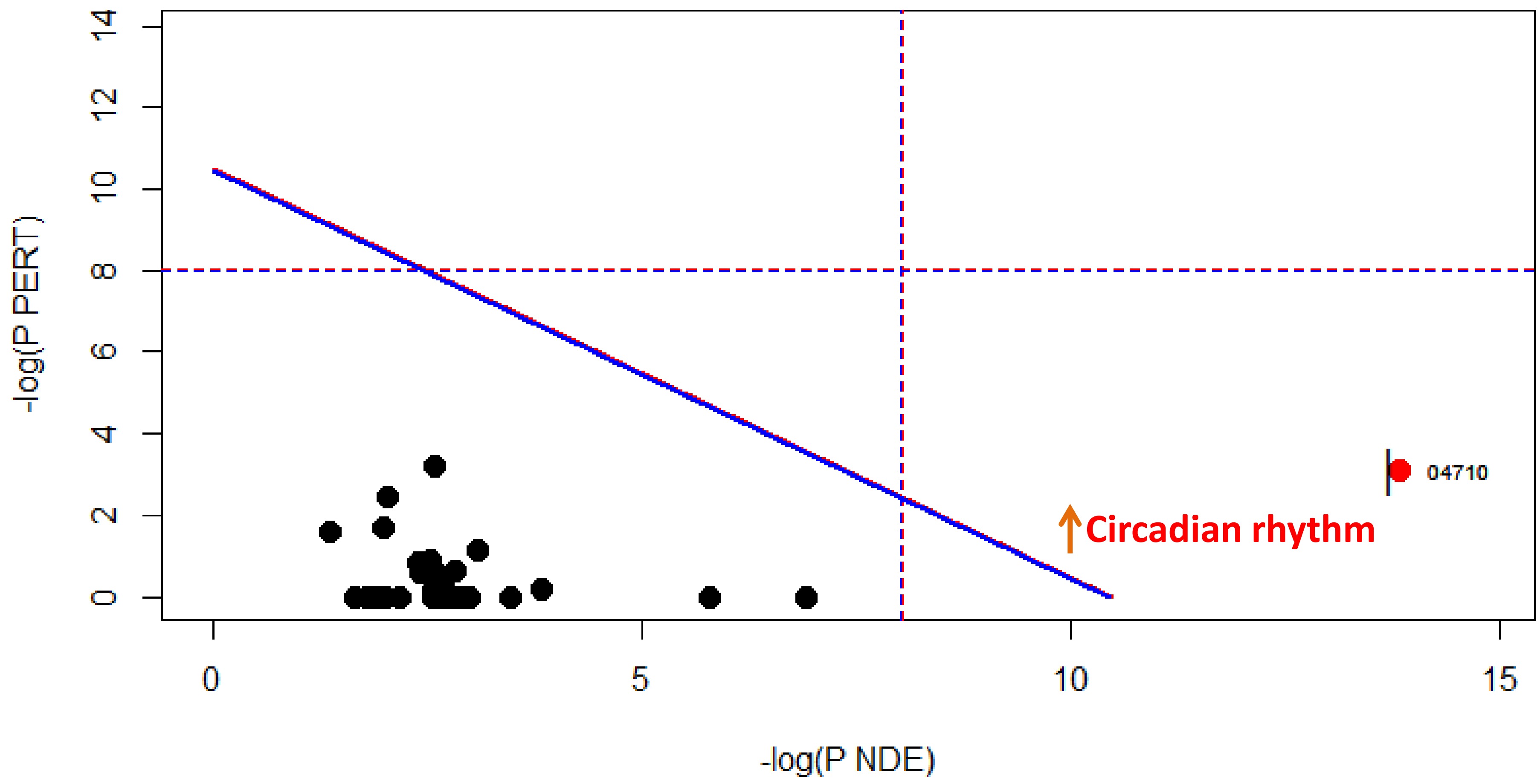

Cd151KO-1.5-fold-DEGs (43; 28 Up; 15 Down)

Figure S3: SPIA two-way evidence plot for Cd151KO 1.5FC sDEGs. KEGG pathway hsa04710: "Circadian rhythm" was 'Activated' in Cd151KO 1.5FC sDEGs.

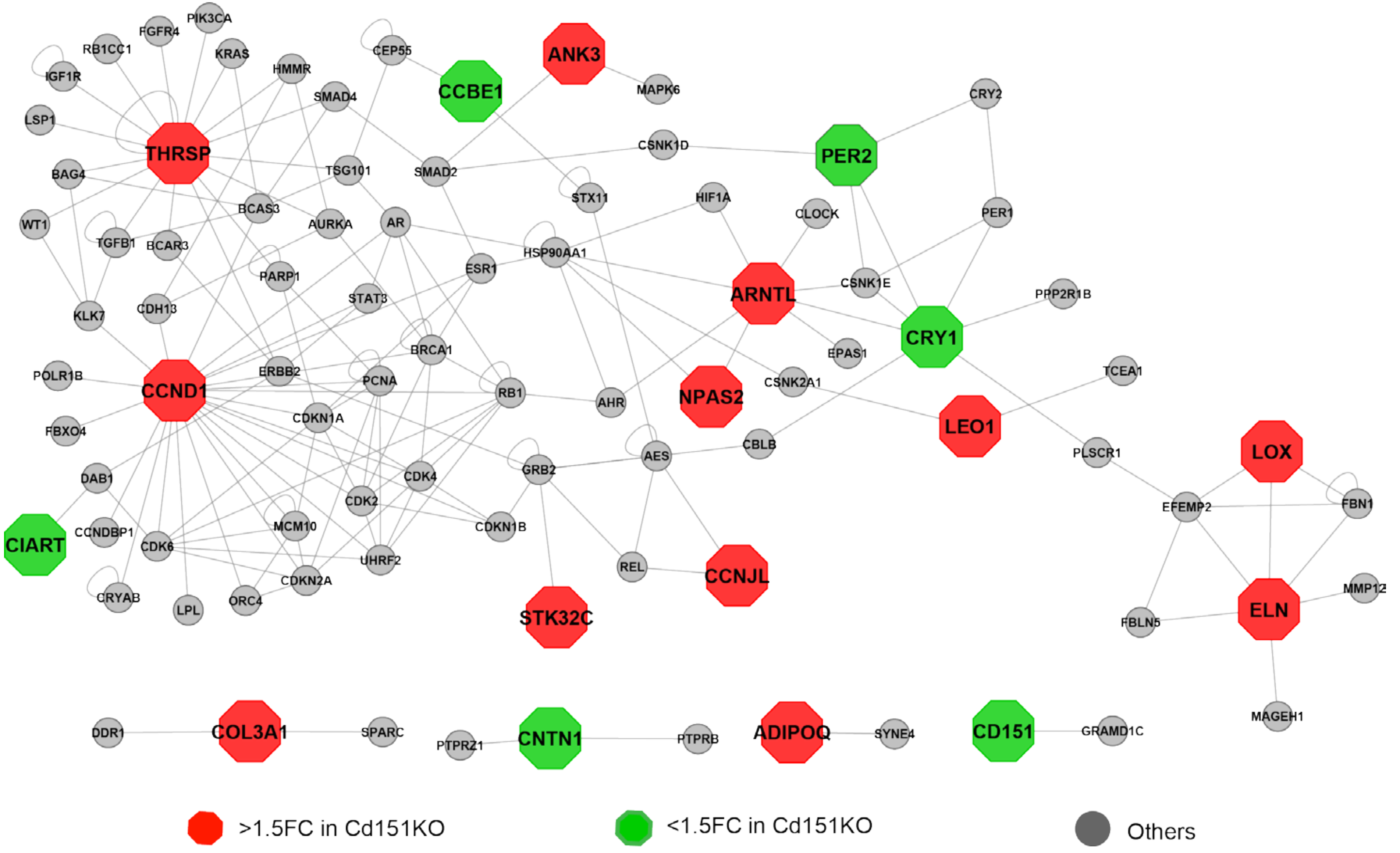

Figure S4A: Cd151KO 1.5FC sDEGs orthologous lung tissue filtered HCDP network.

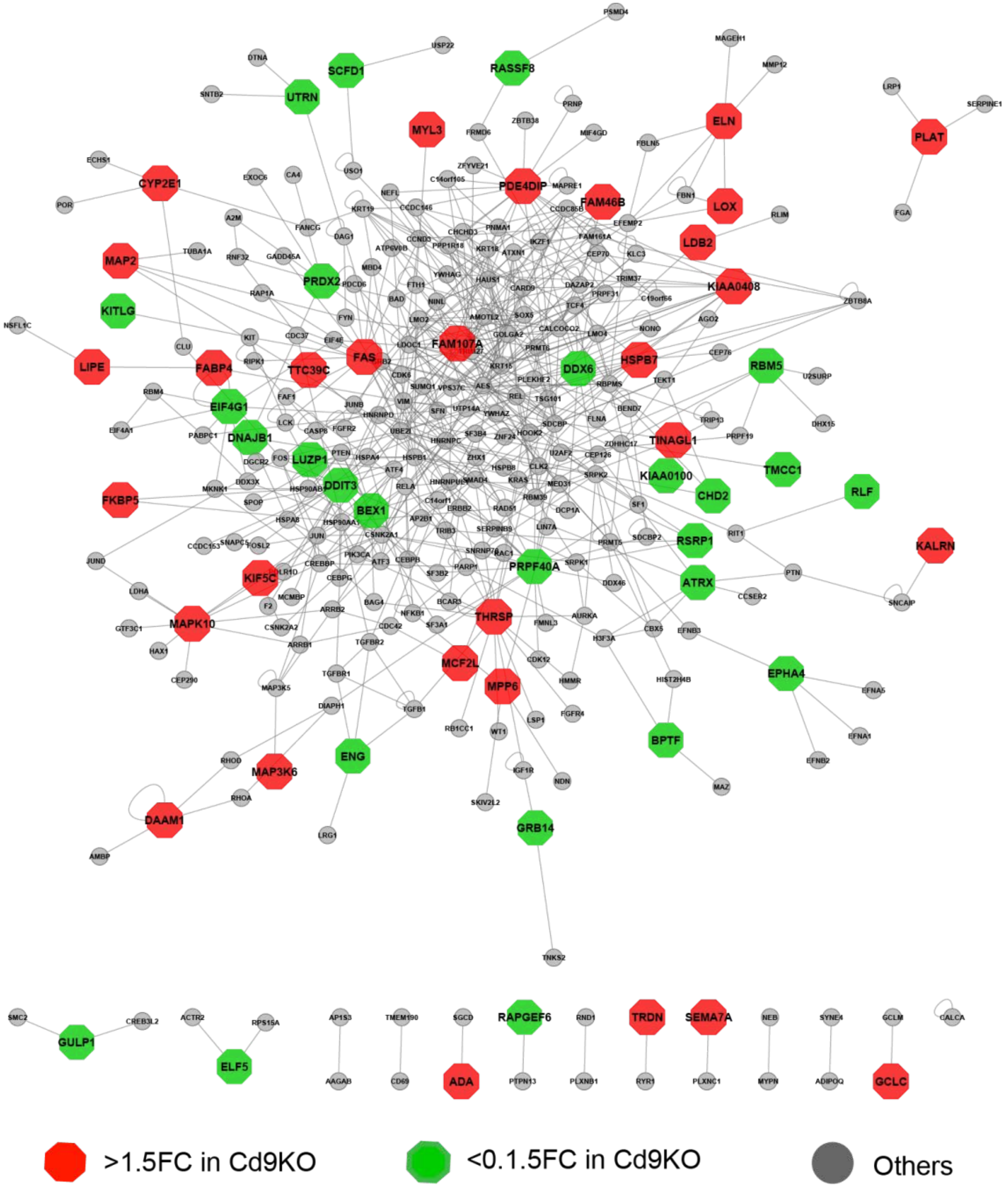

Figure S4B: Cd9KO 1.5FC sDEGs orthologous lung tissue filtered HCDP network.

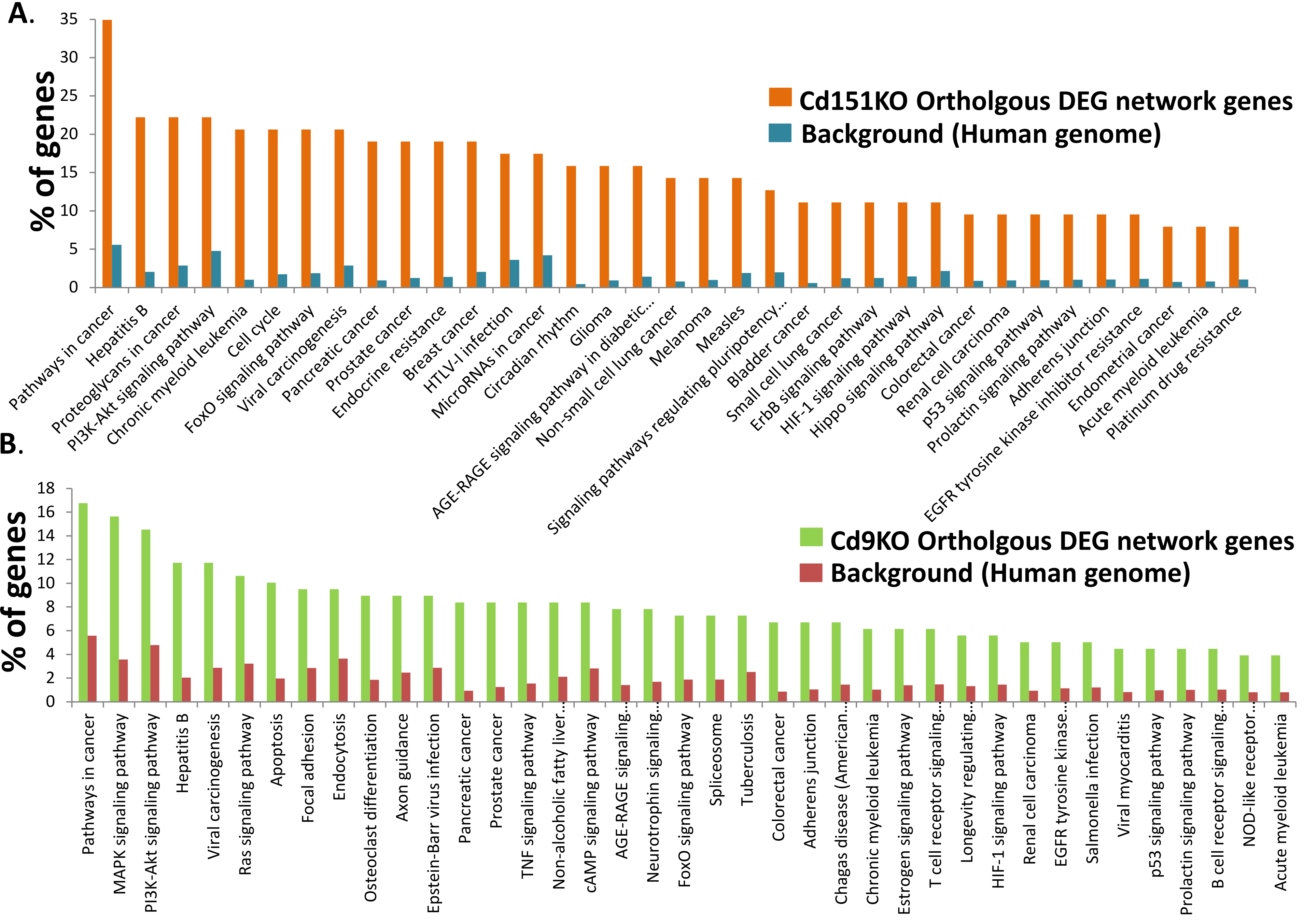

Figure S5: Enriched KEGG pathway associations ($p \leq 0.005$) for the constituents of the A. Cd151KO and B. Cd9KO; 1.5FC sDEGs orthologous lung tissue filtered HCDP networks.

Supplementary Table S1A: List of significantly differentially expressed genes (sDEGs) in Cd151KO vs WT

Upregulated genes

Probe ID	Gene ID	Gene Symbol	Name	Fold change	p-value
1449461_at	63954	Rbp7	retinol binding protein 7, cellular	2.753068644	0.011809862
1425099_a_at	11865	Arntl	aryl hydrocarbon receptor nuclear translocator-like	2.493443118	0.00208667
1415965_at	20249	Scd1	stearoyl-Coenzyme A desaturase 1	2.032649907	0.003133513
1424737_at	21835	Thrsp	thyroid hormone responsive	1.927859456	0.024854275
1423530_at	57740	Stk32c	serine/threonine kinase 32C	1.754062277	0.00035814
1417860_a_at	100689	Spon2	spondin 2, extracellular matrix protein	1.72589082	0.003467361
1430567_at	72432	Spink5	serine peptidase inhibitor, Kazal type 5	1.700085635	0.038003912
1416121_at	16948	Lox	lysyl oxidase	1.670810994	0.01619691
1417419_at	12443	Ccnd1	cyclin D1	1.658267095	0.000163649
1421037_at	18143	Npas2	neuronal PAS domain protein 2	1.649698932	0.020221381
1453351_at	57246	Tbx20	T-box 20	1.649670004	0.025319432
1417867_at	11537	Cfd	complement factor D (adipsin)	1.609174473	0.003286849
1434709_at	319504	Nrcam	neuronal cell adhesion molecule	1.607122508	0.003175798
1427056_at	235130	Adamts15	a disintegrin-like and metallopeptidase (reprolysin type) with thrombospondin type 1 motif, 15	1.592328518	0.048336618
1436789_at	380694	Ccnjl	cyclin J-like	1.573495279	0.004077163
1427884_at	12825	Col3a1	collagen, type III, alpha 1	1.56738354	0.000633731
1447259_at	11735	Ank3	ankyrin 3, epithelial	1.562048134	0.000554436
1422651_at	11450	Adipoq	adiponectin, C1Q and collagen domain containing	1.561764894	0.009119891
1424214_at	231440	Parm1	prostate androgen-regulated mucin-like protein 1	1.55700487	0.003983266
1455293_at	235497	Leo1	Leo1, Paf1/RNA polymerase II complex component	1.555914098	0.003459309
1418454_at	50530	Mfap5	microfibrillar associated protein 5	1.536831541	0.000852465
1452865_at	76612	Lrrc27	leucine rich repeat containing 27	1.53314922	0.000694214
1436453_at	101918	BB144871	expressed sequence BB144871	1.526558048	0.040265024
1418722_at	18054	Ngp	neutrophilic granule protein	1.52599091	0.03912642
1420855_at	13717	Eln	elastin	1.522211876	0.003443711
1457042_at	58871	AI256396	EST AI256396	1.513596906	0.005667979
1428223_at	76574	Mfsd2a	major facilitator superfamily domain containing 2A	1.507399865	0.04067124
1417803_at	66183	Sptssb	serine palmitoyltransferase, small subunit B	1.50366407	0.043601957

Dowregulated genes

Probe ID	Gene ID	Gene Symbol	Name	Fold change	p-value
1451232_at	12476	Cd151	CD151 antigen	0.180248341	4.02E-06
1415969_s_at	16483	Kap	kidney androgen regulated protein	0.370243283	0.001162413
1457823_at	16007	Cyr61	cysteine rich protein 61	0.461226537	0.019687919
1438211_s_at	13170	Dbp	D site albumin promoter binding protein	0.535223639	0.017625563
1448276_at	64540	Tspan4	tetraspanin 4	0.580890111	0.000361035
1433733_a_at	12952	Cry1	cryptochrome 1 (photolyase-like)	0.582548971	0.041875027
1434735_at	217082	Hlf	hepatic leukemia factor	0.603118806	0.00071917
1438033_at	21685	Tef	thyrotroph embryonic factor	0.617127023	0.00012679
1437385_at	320924	Ccbe1	collagen and calcium binding EGF domains 1	0.636097025	0.001921162
1452981_at	12805	Cntn1	contactin 1	0.645885482	0.027900171
1460368_at	227157	Mpp4	membrane protein, palmitoylated 4 (MAGUK p55 subfamily member 4)	0.648749062	0.000910796
1421087_at	18628	Per3	period circadian clock 3	0.650812854	0.002386822
1435188_at	229599	Ciart	circadian associated repressor of transcription	0.653872782	0.013282382
1417603_at	18627	Per2	period circadian clock 2	0.657313423	0.004496313
1456653_a_at	270685	Mthfd1l	methylenetetrahydrofolate dehydrogenase (NADP+ dependent) 1-like	0.66437632	0.001502657

Supplementary Table S1B: List of significantly differentially expressed genes (sDEGs) in Cd9KO vs WT

Upregulated genes

Probe ID	Gene ID	Gene Symbol	Name	Fold change	*p* -value
1455735_at	252903	Ap1s3	adaptor-related protein complex AP-1, sigma 3	3.030834562	0.049071511
1424701_at	219257	Pcdh20	protocadherin 20	2.635954162	0.005146317
1434202_a_at	268709	Fam107a	family with sequence similarity 107, member A	2.55198849	0.027565189
1434194_at	17756	Map2	microtubule-associated protein 2	2.437746888	0.004003786
1416125_at	14229	Fkbp5	FK506 binding protein 5	2.376620856	0.040391969
1424749_at	69368	Wdfy1	WD repeat and FYVE domain containing 1	2.132061705	0.014347374
1434639_at	208439	Klhl29	kelch-like 29	2.117178392	0.003255585
1460601_at	245049	Myrip	myosin VIIA and Rab interacting protein	2.001518106	0.001827825
1427056_at	235130	Adamts15	a disintegrin-like and metallopeptidase (reprolysin type) with thrombospondin type 1 motif, 15	1.930829713	0.004276544
1423439_at	18534	Pck1	phosphoenolpyruvate carboxykinase 1, cytosolic	1.930275484	0.048245578
1436453_at	101918	BB144871	expressed sequence BB144871	1.903804544	0.030978325
1431035_at	208846	Daam1	dishevelled associated activator of morphogenesis 1	1.847453167	0.027009784
1428223_at	76574	Mfsd2a	major facilitator superfamily domain containing 2A	1.792199829	7.71E-06
1416121_at	16948	Lox	lysyl oxidase	1.788609495	0.000571077
1421078_at	69852	Tcf23	transcription factor 23	1.785694976	0.024235372
1430584_s_at	12350	Car3	carbonic anhydrase 3	1.778298684	0.008641935
1424737_at	21835	Thrsp	thyroid hormone responsive	1.770595421	0.011174327
1451801_at	76757	Trdn	triadin	1.770171076	0.001463912
1449901_a_at	53608	Map3k6	mitogen-activated protein kinase kinase kinase 6	1.753798815	0.049085196
1451263_a_at	11770	Fabp4	fatty acid binding protein 4, adipocyte	1.743366192	0.010634292
1453351_at	57246	Tbx20	T-box 20	1.74187999	0.000890714
1419144_at	93671	Cd163	CD163 antigen	1.741859317	0.00061453
1437983_at	58198	Sall1	sal-like 1 (Drosophila)	1.735816903	0.033420862
1455338_at	239559	A4galt	alpha 1,4-galactosyltransferase	1.727620467	0.000869447
1417109_at	94242	Tinagl1	tubulointerstitial nephritis antigen-like 1	1.712883328	0.021025166
1417273_at	27273	Pdk4	pyruvate dehydrogenase kinase, isoenzyme 4	1.695477597	0.01746347
1459903_at	20361	Sema7a	sema domain, immunoglobulin domain (Ig), and GPI membrane anchor, (semaphorin) 7A	1.68654962	0.046910173
1437492_at	210719	Mkx	mohawk homeobox	1.673543534	0.000534895

1438752_at	320270	A230058F20Rik	RIKEN cDNA A230058F20 gene	1.670709286	0.000294984
1415806_at	18791	Plat	plasminogen activator, tissue	1.670201987	0.016960584
1448826_at	17888	Myh6	myosin, heavy polypeptide 6, cardiac muscle, alpha	1.65966242	0.002484537
1442341_at	545156	Kalrn	kalirin, RhoGEF kinase	1.655626764	0.002061015
1416025_at	99571	Fgg	fibrinogen gamma chain	1.641363955	0.005403825
1455959_s_at	14629	Gclc	glutamate-cysteine ligase, catalytic subunit	1.633053896	0.022416768
1420855_at	13717	Eln	elastin	1.626248061	0.000908583
1460371_at	72630	Hspa12b	heat shock protein 12B	1.622604361	0.04362918
1439263_at	14210	LOC14210	hypothetical LOC14210 [Mus musculus (house mouse)]	1.621644634	0.022050498
1455266_at	16574	Kif5c	kinesin family member 5C	1.620079921	0.016996146
1421289_at	29818	Hspb7	heat shock protein family, member 7 (cardiovascular)	1.613086761	0.002150225
1458379_at	212448	9330159F19Rik	RIKEN cDNA 9330159F19 gene	1.611483103	0.040348357
1429274_at	71897	Lypd6b	LY6/PLAUR domain containing 6B	1.608282866	0.00849519
1417600_at	57738	Slc15a2	solute carrier family 15 (H+/peptide transporter), member 2	1.606875554	0.040746485
1431014_at	330409	Cecr2	cat eye syndrome chromosome region, candidate 2	1.60420783	0.027333501
1435813_at	68802	Mypn	myopalladin	1.599292208	0.001907786
1417976_at	11486	Ada	adenosine deaminase	1.592059101	0.028928885
1441693_at	330119	Adamts3	a disintegrin-like and metallopeptidase (reprolysin type) with thrombospondin type 1 motif, 3	1.590692803	0.049639816
1437523_s_at	24053	Sgcg	sarcoglycan, gamma (dystrophin-associated glycoprotein)	1.571849704	0.016606917
1417988_at	19711	Resp18	regulated endocrine-specific protein 18	1.5710283	0.00014047
1456786_at	16826	Ldb2	LIM domain binding 2	1.566959609	0.003121982
1437195_x_at	26414	Mapk10	mitogen-activated protein kinase 10	1.558980221	0.013219847
1427768_s_at	17897	Myl3	myosin, light polypeptide 3	1.557599738	0.004382667
1428942_at	17750	Mt2	metallothionein 2	1.554327236	0.036732622
1417256_at	17386	Mmp13	matrix metallopeptidase 13	1.552725479	0.003526673
1434728_at	53623	Gria3	glutamate receptor, ionotropic, AMPA3 (alpha 3)	1.548187008	0.004168762
1453395_at	76675	5330403D14Rik	RIKEN cDNA 5330403D14 gene	1.545402568	0.03062905
1428352_at	70807	Arrdc2	arrestin domain containing 2	1.543142244	0.000654184
1425917_at	15061	Ifi44l	interferon-induced protein 44 like	1.542382865	0.008969657
1417803_at	66183	Sptssb	serine palmitoyltransferase, small subunit B	1.541466319	0.043734467
1433877_at	100342	Fam46b	family with sequence similarity 46, member B	1.533957319	0.009011349
1422651_at	11450	Adipoq	adiponectin, C1Q and collagen domain containing	1.529442072	0.047843475

1417626_at	83679	Pde4dip	phosphodiesterase 4D interacting protein (myomegalin)	1.525575535	0.001061385
1452565_x_at	641050	LOC641050		1.524678459	0.012973328
1426223_at	72747	Ttc39c	tetratricopeptide repeat domain 39C	1.520112429	0.013228397
1453523_at	77914	Krtap17-1	keratin associated protein 17-1	1.518686666	0.019153721
1422820_at	16890	Lipe	lipase, hormone sensitive	1.516111807	0.000477274
1435116_at	74393	Map10	microtubule-associated protein 10	1.511775275	0.044941963
1434140_at	17207	Mcf2l	mcf.2 transforming sequence-like	1.50972709	0.000572349
1444974_at	101769	AU023617	expressed sequence AU023617	1.508517663	0.019819127
1427355_at	12310	Calca	calcitonin/calcitonin-related polypeptide, alpha	1.506754604	0.008430772
1460251_at	14102	Fas	Fas (TNF receptor superfamily member 6)	1.504566374	0.003904877
1415994_at	13106	Cyp2e1	cytochrome P450, family 2, subfamily e, polypeptide 1	1.504021718	0.003423785
1449071_at	17898	Myl7	myosin, light polypeptide 7, regulatory	1.501503728	0.008848852

Downregulated genes

**Probe ID**	**Gene ID**	**Gene Symbol**	**Name**	**Fold change**	***p*-value**
1415916_a_at	12527	Cd9	CD9 antigen	0.082399255	1.47E-07
1415969_s_at	16483	Kap	kidney androgen regulated protein	0.360568152	0.000908465
1416066_at	319996	Casc4	cancer susceptibility candidate 4	0.443779703	0.001395071
1416755_at	72289	Malat1	metastasis associated lung adenocarcinoma transcript 1 (non-coding RNA)	0.52715332	0.005550731
1417388_at	68169	Ndnf	neuron-derived neurotrophic factor	0.550164584	0.017755531
1419356_at	12069	Bex2	brain expressed X-linked 2	0.55346977	0.003953698
1419555_at	21672	Prdx2	peroxiredoxin 2	0.57961536	0.004760092
1420946_at	109263	Rlf	rearranged L-myc fusion sequence	0.580072835	0.001091695
1421168_at	27981	Rsrp1	arginine/serine rich protein 1	0.590466701	0.001786357
1421955_a_at	330401	Tmcc1	transmembrane and coiled coil domains 1	0.59120956	0.000128941
1422330_at	108156	Mthfd1	methylenetetrahydrofolate dehydrogenase (NADP+ dependent), methenyltetrahydrofolate cyclohydrolase, formyltetrahydrofolate synthase	0.592073248	0.033240575
1422924_at	22288	Utrn	utrophin	0.603255449	0.015952387
1423594_a_at	72153	2610020P09Rik	RIKEN cDNA 2610020P09 gene	0.60326305	0.008454289
1424598_at	70297	Gcc2	GRIP and coiled-coil domain containing 2	0.605111937	0.001198923
1425445_a_at	13209	Ddx6	DEAD (Asp-Glu-Ala-Asp) box polypeptide 6	0.609610181	0.010111066

1426152_a_at	17311	Kitl	kit ligand	0.609801494	0.010552636
1426892_at	99946	6720422M22Rik	RIKEN cDNA 6720422M22 gene	0.609839701	0.011393557
1427037_at	81489	Dnajb1	DnaJ heat shock protein family (Hsp40) member B1	0.610217041	0.04184665
1427285_s_at	70676	Gulp1	GULP, engulfment adaptor PTB domain containing 1	0.610328374	0.000862581
1427311_at	70545	5730433N10Rik	RIKEN cDNA 5730433N10 gene	0.610614876	0.04310588
1428735_at	22589	Atrx	alpha thalassemia/mental retardation syndrome X-linked	0.611242729	0.009582188
1429679_at	76983	Scfd1	Sec1 family domain containing 1	0.611387171	6.31E-05
1430642_at	76950	2900001G08Rik	RIKEN cDNA 2900001G08 gene	0.615573824	0.005396758
1430979_a_at	207165	Bptf	bromodomain PHD finger transcription factor	0.617900205	0.007044957
1431686_a_at	18703	Pigr	polymeric immunoglobulin receptor	0.6234868	0.02018545
1432092_a_at	319341	C230007H23Rik	RIKEN cDNA C230007H23 gene	0.624977705	0.008613194
1432746_at	30785	Cttnbp2	cortactin binding protein 2	0.625928022	0.006875355
1433988_s_at	77070	6030442H21Rik	RIKEN cDNA 6030442H21 gene	0.628953086	0.004372328
1435435_at	73107	2900092N22Rik	RIKEN cDNA 2900092N22 gene	0.630367489	0.021463917
1437403_at	13838	Epha4	Eph receptor A4	0.632515726	0.002709529
1437798_at	212285	Arap2	ArfGAP with RhoGAP domain, ankyrin repeat and PH domain 2	0.63354219	0.006065759
1438033_at	57748	Jmy	junction-mediating and regulatory protein	0.633744931	0.015874542
1438069_a_at	399614	C130009A20Rik	RIKEN cDNA C130009A20 gene	0.636509344	6.49E-06
1438713_at	74511	Lrrc17	leucine rich repeat containing 17	0.637154342	0.009972009
1438732_at	52175	DXErtd242e	DNA segment, Chr X, ERATO Doi 242, expressed	0.637912678	0.004096726
1439555_at	269593	Luzp1	leucine zipper protein 1	0.639843189	0.010271795
1439757_s_at	21950	Tnfsf9	tumor necrosis factor (ligand) superfamily, member 9	0.641006015	0.022259369
1440248_at	50915	Grb14	growth factor receptor bound protein 14	0.641845922	0.00170258
1441370_at	208643	Eif4g1	eukaryotic translation initiation factor 4, gamma 1	0.642384567	0.02362717
1443589_at	63985	Gmfb	glia maturation factor, beta	0.642530415	0.005874986
1443682_at	320825	Samd5	sterile alpha motif domain containing 5	0.642917177	0.008766321
1443877_a_at	13711	Elf5	E74-like factor 5	0.642968835	0.030578171
1443897_at	13198	Ddit3	DNA-damage inducible transcript 3	0.644578312	0.000192067
1444109_at	192786	Rapgef6	Rap guanine nucleotide exchange factor (GEF) 6	0.645251353	0.00574795
1444845_at	102153	C230098O21Rik	RIKEN cDNA C230098O21 gene	0.645259998	0.004916913
1444982_at	72503	2610507B11Rik	RIKEN cDNA 2610507B11 gene	0.646443643	0.00841854
1445843_at	83486	Rbm5	RNA binding motif protein 5	0.646770293	0.007334822

1445881_at	56194	Prpf40a	pre-mRNA processing factor 40A	0.647829843	0.01249293
1448352_at	100042480	Nhsl2	NHS-like 2	0.649733471	0.009064386
1448538_a_at	12515	Cd69	CD69 antigen	0.650304608	0.030531658
1450035_a_at	27405	Abcg3	ATP-binding cassette, sub-family G (WHITE), member 3	0.650953573	0.026689441
1450060_at	244059	Chd2	chromodomain helicase DNA binding protein 2	0.651144197	0.00950274
1451800_at	215113	Slc43a2	solute carrier family 43, member 2	0.651322332	0.016025951
1451878_a_at	56492	Cldn18	claudin 18	0.651677904	0.0022487
1453225_at	223881	Rnd1	Rho family GTPase 1	0.653071746	0.036344177
1453627_at	13618	Ednrb	endothelin receptor type B	0.653453584	0.008876693
1454460_at	93691	Klf7	Kruppel-like factor 7 (ubiquitous)	0.661668089	0.01591224
1454472_at	14652	Glp1r	glucagon-like peptide 1 receptor	0.662448045	0.00763503
1455197_at	13805	Eng	endoglin	0.663429935	0.010245376
1455905_at	21685	Tef	thyrotroph embryonic factor	0.66425	0.033039385
1456337_at	71323	Rassf8	Ras association (RalGDS/AF-6) domain family (N-terminal) member 8	0.665614528	0.002737786

Supplementary Table S2: Cd151KO and Cd9KO sDEGs- Enriched biological themes (*p* ≤0.05)

Cd151KO

Enriched KEGG Pathways

Identifier	Name	Associated genes	*p* -value
mmu04710	Circadian rhythm	Arntl,Cry1,Npas2,Per2,Per3	5.95E-07

Enriched Reactome Pathways

Identifier	Name	Associated genes	*p* -value
R-MMU-1368108	BMAL1:CLOCK,NPAS2 activates circadian gene expression	Arntl,Cry1,Dbp,Npas2,Per2	2.95E-07
R-MMU-1368110	Bmal1:Clock,Npas2 activates circadian gene expression	Arntl,Cry1,Dbp,Npas2,Per2	2.95E-07
R-MMU-400253	Circadian Clock	Arntl,Cry1,Dbp,Npas2,Per2	1.4937E-06
R-MMU-508751	Circadian Clock	Arntl,Cry1,Dbp,Npas2,Per2	2.01356E-06
R-MMU-1989781	PPARA activates gene expression	Arntl,Npas2	0.010064828
R-MMU-1566948	Elastic fibre formation	Eln,Lox,Mfap5	0.017921022
R-MMU-447043	Neurofascin interactions	Cntn1,Nrcam	0.017921022
R-MMU-1368082	RORA activates gene expression	Arntl,Npas2	0.021921545
R-MMU-5334727	Mus musculus biological processes	Arntl,Cry1,Dbp,Npas2,Per2	0.023799003
R-MMU-1368092	Rora activates gene expression	Arntl,Npas2	0.029977063
R-MMU-1474244	Extracellular matrix organization	Cd151,Col3a1,Eln,Lox,Mfap5	0.036040324
R-MMU-2022090	Assembly of collagen fibrils and other multimeric structures	Cd151,Col3a1,Lox	0.036814617

Cd9KO

Enriched Gene Ontology [GO] (Slim)- Biological Process term associations

Identifier	Name	Associated genes	*p* -value
GO:0003013	circulatory system process	11450, 11486, 12310, 13618, 13805, 14629, 14652, 17888, 17897, 24053, 57246, 76757, 99571	0.0000028

GO:0048856	anatomical structure development	11450, 11486, 12310, 12527, 13198, 13209, 13618, 13711, 13717, 13805, 13838, 14102, 16574, 16826, 16948, 17311, 17386, 17756, 17888, 17897, 19711, 20361, 21672, 21950, 22288, 22589, 24053, 56194, 56492, 57246, 68169, 68802, 74511, 76574, 76983, 93691, 108156, 208643, 210719, 223881, 244059, 269593, 330409, 545156	0.0072332
GO:0042592	homeostatic process	11450, 11486, 11770, 12310, 13198, 13618, 14102, 14629, 14652, 16826, 17311, 17750, 21672, 22589, 27273, 56492, 76757, 108156	0.009772
GO:0030154	cell differentiation	11450, 11486, 11770, 12310, 13198, 13209, 13618, 13711, 13805, 13838, 14102, 16574, 17311, 17756, 17888, 20361, 21672, 21950, 22589, 24053, 56492, 57246, 68169, 68802, 74511, 76574, 93691, 208643, 210719, 223881, 244059, 330409, 545156	0.009772
GO:0030198	extracellular matrix organization	13717, 13805, 16948, 17386, 68169, 210719	0.0255129

Enriched Gene Ontology [GO]- Biological Process term associations

**Identifier**	**Name**	**Associated genes**	***p*-value**
GO:0008015	blood circulation	11450, 11486, 12310, 13618, 13805, 14629, 14652, 17888, 17897, 24053, 57246, 76757, 99571	0.0001939
GO:0003013	circulatory system process	11450, 11486, 12310, 13618, 13805, 14629, 14652, 17888, 17897, 24053, 57246, 76757, 99571	0.0001939

Supplementary Table S3: KEGG pathways significantly perturbed in Cd151KO and Cd9KO as identified by SPIA

Cd151KO- 1.5FC

Name	ID	pSize	NDE	pNDE	tA	pPERT	pG	pGFdr	pGFWER	Status
Circadian rhythm - mammal	4710	22	5	3.88E-11	4.513228	0.046	5.00E-11	1.55E-09	1.55E-09	Activated

Cd151KO- 1.2FC

Name	ID	pSize	NDE	pNDE	tA	pPERT	pG	pGFdr	pGFWER	Status
ECM-receptor interaction	4512	84	9	4.17E-07	2.767403	0.004	3.53E-08	3.71E-06	3.71E-06	Activated
Focal adhesion	4510	199	13	4.16E-07	6.70457	0.023	1.86E-07	7.54E-06	1.96E-05	Activated
Circadian rhythm - mammal	4710	22	5	3.72E-06	3.877921	0.003	2.15E-07	7.54E-06	2.26E-05	Activated

Cd9KO- 1.2FC

Name	ID	pSize	NDE	pNDE	tA	pPERT	pG	pGFdr	pGFWER	Status
Focal adhesion	4510	199	26	3.05E-08	7.278188	0.091	5.74E-08	7.34E-06	7.34E-06	Activated
Dilated cardiomyopathy	5414	88	17	2.32E-08	-0.40675	0.539	2.40E-07	1.54E-05	3.07E-05	Inhibited
Calcium signaling pathway	4020	175	23	1.68E-07	-2.716603	0.202	6.17E-07	2.63E-05	7.90E-05	Inhibited
MAPK signaling pathway	4010	261	28	6.05E-07	0.745982	0.687	6.52E-06	0.000209	0.000834	Activated
Chemokine signaling pathway	4062	172	21	1.92E-06	0.486672	0.896	2.46E-05	0.00063	0.00315	Activated

Viral carcinogenesis	5203	185	20	2.09E-05	0.458835	0.102	3.00E-05	0.00064	0.003842	Activated
Transcriptional misregulation in cancer	5202	161	20	2.56E-06	0	1	3.55E-05	0.00065	0.004547	Inhibited
Dopaminergic synapse	4728	125	15	6.65E-05	1.808221	0.058	5.19E-05	0.000831	0.00665	Activated
TGF-beta signaling pathway	4350	80	12	3.93E-05	-3.215364	0.138	7.12E-05	0.000923	0.009108	Inhibited
Tight junction	4530	128	17	5.73E-06	-0.012675	0.959	7.21E-05	0.000923	0.009225	Inhibited

KEGGLINK

http://www.genome.jp/dbget-bin/show_pathway?mmu04710+12952+11865+18143+18627+18628

KEGGLINK

http://www.genome.jp/dbget-bin/show_pathway?mmu04512+16782+12825+12832+12842+53867+16416+16404+12643+19699

http://www.genome.jp/dbget-bin/show_pathway?mmu04510+16416+16404+19877+12643+12825+12832+12842+16782+19699+53867+11798+12443+16000

http://www.genome.jp/dbget-bin/show_pathway?mmu04710+12952+11865+18143+18626+18628

KEGGLINK

http://www.genome.jp/dbget-bin/show_pathway?mmu04510+16403+68794+17898+19877+18607+170736+16202+18710+11472+11855+18750+14360+11848+12826+12832+12843+16775+11798+109880+14254+14205+54635+26414+214230+57257+56637

http://www.genome.jp/dbget-bin/show_pathway?mmu05414+12293+11938+16403+24053+21954+21924+21956+22003+17897+22138+104110+11514+11515+18821+17888+21803+21808

http://www.genome.jp/dbget-bin/show_pathway?mmu04020+320404+11514+18127+108058+18750+12494+14676+20698+16439+20192+18795+12425+13618+15559+54140+57260+58861+21924+104110+11515+11938+18821+67972

http://www.genome.jp/dbget-bin/show_pathway?mmu04010+19418+63953+17762+109880+18750+19395+240168+14183+12293+14167+14168+18205+12064+13198+216965+381921+53608+26412+23882+21812+14102+21803+21808+26405+26414+216869+68794+225028

http://www.genome.jp/dbget-bin/show_pathway?mmu04062+12765+20292+20305+15162+11848+18710+18795+14706+14773+216869+19877+57257+104110+11514+11515+19395+93742+18762+109880+18035+56637

http://www.genome.jp/dbget-bin/show_pathway?mmu05203+54131+19645+19651+107932+22029+16451+18710+14534+252870+22627+12914+242341+12576+208727+232232+11848+11472+18035+19106+68024

http://www.genome.jp/dbget-bin/show_pathway?mmu05202+14030+13876+14009+13198+14254+56312+22029+12048+16909+16599+22289+12576+18647+17132+17283+20474+15446+53623+15078+18791

http://www.genome.jp/dbget-bin/show_pathway?mmu04728+18795+16439+18750+14681+14706+56637+26414+108058+216869+235542+26931+16574+14802+53623+14812

http://www.genome.jp/dbget-bin/show_pathway?mmu04350+12914+19651+72508+19877+11848+21812+21803+21808+13179+110542+12168+12161

http://www.genome.jp/dbget-bin/show_pathway?mmu04530+18750+56492+58220+93742+269587+17475+12995+20742+17880+17888+17898+11848+192195+18762+70737+11472+99470

Supplementary Table S4: Human orthologues of Cd151KO and Cd9KO sDEGs

Cd151KO

sDEG ID (Mouse)	sDEG Symbol (Mouse)	sDEG name (Mouse)	Orthologous Gene ID (Human)	Orthologous Gene Symbol (Human)	Orthologous Gene name (Human)
100689	Spon2	spondin 2, extracellular matrix protein	10417	SPON2	spondin 2
72432	Spink5	serine peptidase inhibitor, Kazal type 5	11005	SPINK5	serine peptidase inhibitor, Kazal type 5
63954	Rbp7	retinol binding protein 7, cellular	116362	RBP7	retinol binding protein 7
235497	Leo1	Leo1, Paf1/RNA polymerase II complex component	123169	LEO1	LEO1 homolog, Paf1/RNA polymerase II complex component
12805	Cntn1	contactin 1	1272	CNTN1	contactin 1
12825	Col3a1	collagen, type III, alpha 1	1281	COL3A1	collagen type III alpha 1 chain
12952	Cry1	cryptochrome 1 (photolyase-like)	1407	CRY1	cryptochrome circadian clock 1
320924	Ccbe1	collagen and calcium binding EGF domains 1	147372	CCBE1	collagen and calcium binding EGF domains 1
229599	Ciart	circadian associated repressor of transcription	148523	CIART	circadian associated repressor of transcription
13170	Dbp	D site albumin promoter binding protein	1628	DBP	D-box binding PAR bZIP transcription factor
66183	Sptssb	serine palmitoyltransferase, small subunit B	165679	SPTSSB	serine palmitoyltransferase small subunit B
11537	Cfd	complement factor D (adipsin)	1675	CFD	complement factor D
235130	Adamts15	a disintegrin-like and metallopeptidase (reprolysin type) with thrombospondin type 1 motif, 15	170689	ADAMTS15	ADAM metallopeptidase with thrombospondin type 1 motif 15
13717	Eln	elastin	2006	ELN	elastin
231440	Parm1	prostate androgen-regulated mucin-like protein 1	25849	PARM1	prostate androgen-regulated mucin-like protein 1
270685	Mthfd1l	methylenetetrahydrofolate dehydrogenase (NADP+ dependent) 1-like	25902	MTHFD1L	methylenetetrahydrofolate dehydrogenase (NADP+ dependent) 1-like
57740	Stk32c	serine/threonine kinase 32C	282974	STK32C	serine/threonine kinase 32C
11735	Ank3	ankyrin 3, epithelial	288	ANK3	ankyrin 3
217082	Hlf	hepatic leukemia factor	3131	HLF	HLF, PAR bZIP transcription factor
16007	Cyr61	cysteine rich protein 61	3491	CYR61	cysteine rich angiogenic inducer 61
16948	Lox	lysyl oxidase	4015	LOX	lysyl oxidase

11865	Arntl	aryl hydrocarbon receptor nuclear translocator-like	406	ARNTL	aryl hydrocarbon receptor nuclear translocator like
18143	Npas2	neuronal PAS domain protein 2	4862	NPAS2	neuronal PAS domain protein 2
319504	Nrcam	neuronal cell adhesion molecule	4897	NRCAM	neuronal cell adhesion molecule
57246	Tbx20	T-box 20	57057	TBX20	T-box 20
227157	Mpp4	membrane protein, palmitoylated 4 (MAGUK p55 subfamily member 4)	58538	MPP4	membrane palmitoylated protein 4
12443	Ccnd1	cyclin D1	595	CCND1	cyclin D1
21685	Tef	thyrotroph embryonic factor	7008	TEF	TEF, PAR bZIP transcription factor
21835	Thrsp	thyroid hormone responsive	7069	THRSP	thyroid hormone responsive
64540	Tspan4	tetraspanin 4	7106	TSPAN4	tetraspanin 4
380694	Ccnjl	cyclin J-like	79616	CCNJL	cyclin J like
76612	Lrrc27	leucine rich repeat containing 27	80313	LRRC27	leucine rich repeat containing 27
50530	Mfap5	microfibrillar associated protein 5	8076	MFAP5	microfibrillar associated protein 5
76574	Mfsd2a	major facilitator superfamily domain containing 2A	84879	MFSD2A	major facilitator superfamily domain containing 2A
18628	Per3	period circadian clock 3	8863	PER3	period circadian clock 3
18627	Per2	period circadian clock 2	8864	PER2	period circadian clock 2
11450	Adipoq	adiponectin, C1Q and collagen domain containing	9370	ADIPOQ	adiponectin, C1Q and collagen domain containing
12476	Cd151	CD151 antigen	977	CD151	CD151 molecule (Raph blood group)

Cd9KO

**sDEG ID (Mouse)**	**sDEG Symbol (Mouse)**	**sDEG name (Mouse)**	**Orthologous Gene ID (Human)**	**Orthologous Gene Symbol (Human)**	**Orthologous Gene name (Human)**
11486	Ada	adenosine deaminase	100	ADA	adenosine deaminase
83486	Rbm5	RNA binding motif protein 5	10181	RBM5	RNA binding motif protein 5
74511	Lrrc17	leucine rich repeat containing 17	10234	LRRC17	leucine rich repeat containing 17
76757	Trdn	triadin	10345	TRDN	triadin
15061	Ifi44l	interferon-induced protein 44 like	10964	IFI44L	interferon induced protein 44 like
244059	Chd2	chromodomain helicase DNA binding protein 2	1106	CHD2	chromodomain helicase DNA binding protein 2

268709	Fam107a	family with sequence similarity 107, member A	11170	FAM107A	family with sequence similarity 107 member A
71323	Rassf8	Ras association (RalGDS/AF-6) domain family (N-terminal) member 8	11228	RASSF8	Ras association domain family member 8
319996	Casc4	cancer susceptibility candidate 4	113201	CASC4	cancer susceptibility candidate 4
208439	Klhl29	kelch-like 29	114818	KLHL29	kelch like family member 29
100342	Fam46b	family with sequence similarity 46, member B	115572	FAM46B	family with sequence similarity 46 member B
72630	Hspa12b	heat shock protein 12B	116835	HSPA12B	heat shock protein family A (Hsp70) member 12B
212285	Arap2	ArfGAP with RhoGAP domain, ankyrin repeat and PH domain 2	116984	ARAP2	ArfGAP with RhoGAP domain, ankyrin repeat and PH domain 2
215113	Slc43a2	solute carrier family 43, member 2	124935	SLC43A2	solute carrier family 43 member 2
72747	Ttc39c	tetratricopeptide repeat domain 39C	125488	TTC39C	tetratricopeptide repeat domain 39C
252903	Ap1s3	adaptor-related protein complex AP-1, sigma 3	130340	AP1S3	adaptor related protein complex 1 sigma 3 subunit
71897	Lypd6b	LY6/PLAUR domain containing 6B	130576	LYPD6B	LY6/PLAUR domain containing 6B
57748	Jmy	junction-mediating and regulatory protein	133746	JMY	junction mediating and regulatory protein, p53 cofactor
69852	Tcf23	transcription factor 23	150921	TCF23	transcription factor 23
13106	Cyp2e1	cytochrome P450, family 2, subfamily e, polypeptide 1	1571	CYP2E1	cytochrome P450 family 2 subfamily E member 1
13198	Ddit3	DNA-damage inducible transcript 3	1649	DDIT3	DNA damage inducible transcript 3
13209	Ddx6	DEAD (Asp-Glu-Ala-Asp) box polypeptide 6	1656	DDX6	DEAD-box helicase 6
66183	Sptssb	serine palmitoyltransferase, small subunit B	165679	SPTSSB	serine palmitoyltransferase small subunit B
235130	Adamts15	a disintegrin-like and metallopeptidase (reprolysin type) with thrombospondin type 1 motif, 15	170689	ADAMTS15	ADAM metallopeptidase with thrombospondin type 1 motif 15
13618	Ednrb	endothelin receptor type B	1910	EDNRB	endothelin receptor type B
208643	Eif4g1	eukaryotic translation initiation factor 4, gamma 1	1981	EIF4G1	eukaryotic translation initiation factor 4 gamma 1
13711	Elf5	E74-like factor 5	2001	ELF5	E74 like ETS transcription factor 5

13717	Eln	elastin	2006	ELN	elastin
13805	Eng	endoglin	2022	ENG	endoglin
13838	Epha4	Eph receptor A4	2043	EPHA4	EPH receptor A4
11770	Fabp4	fatty acid binding protein 4, adipocyte	2167	FABP4	fatty acid binding protein 4
207165	Bptf	bromodomain PHD finger transcription factor	2186	BPTF	bromodomain PHD finger transcription factor
99571	Fgg	fibrinogen gamma chain	2266	FGG	fibrinogen gamma chain
14229	Fkbp5	FK506 binding protein 5	2289	FKBP5	FK506 binding protein 5
208846	Daam1	dishevelled associated activator of morphogenesis 1	23002	DAAM1	dishevelled associated activator of morphogenesis 1
330401	Tmcc1	transmembrane and coiled coil domains 1	23023	TMCC1	transmembrane and coiled-coil domain family 1
76983	Scfd1	Sec1 family domain containing 1	23256	SCFD1	sec1 family domain containing 1
17207	Mcf2l	mcf.2 transforming sequence-like	23263	MCF2L	MCF.2 cell line derived transforming sequence like
245049	Myrip	myosin VIIA and Rab interacting protein	25924	MYRIP	myosin VIIA and Rab interacting protein
70807	Arrdc2	arrestin domain containing 2	27106	ARRDC2	arrestin domain containing 2
29818	Hspb7	heat shock protein family, member 7 (cardiovascular)	27129	HSPB7	heat shock protein family B (small) member 7
223881	Rnd1	Rho family GTPase 1	27289	RND1	Rho family GTPase 1
14629	Gclc	glutamate-cysteine ligase, catalytic subunit	2729	GCLC	glutamate-cysteine ligase catalytic subunit
14652	Glp1r	glucagon-like peptide 1 receptor	2740	GLP1R	glucagon like peptide 1 receptor
330409	Cecr2	cat eye syndrome chromosome region, candidate 2	27443	CECR2	CECR2, histone acetyl-lysine reader
63985	Gmfb	glia maturation factor, beta	2764	GMFB	glia maturation factor beta
210719	Mkx	mohawk homeobox	283078	MKX	mohawk homeobox
50915	Grb14	growth factor receptor bound protein 14	2888	GRB14	growth factor receptor bound protein 14
53623	Gria3	glutamate receptor, ionotropic, AMPA3 (alpha 3)	2892	GRIA3	glutamate ionotropic receptor AMPA type subunit 3
81489	Dnajb1	DnaJ heat shock protein family (Hsp40) member B1	3337	DNAJB1	DnaJ heat shock protein family (Hsp40) member B1
100042480	Nhsl2	NHS-like 2	340527	NHSL2	NHS like 2
14102	Fas	Fas (TNF receptor superfamily member 6)	355	FAS	Fas cell surface death receptor

16574	Kif5c	kinesin family member 5C	3800	KIF5C	kinesin family member 5C
19711	Resp18	regulated endocrine-specific protein 18	389075	RESP18	regulated endocrine specific protein 18
320825	Samd5	sterile alpha motif domain containing 5	389432	SAMD5	sterile alpha motif domain containing 5
16890	Lipe	lipase, hormone sensitive	3991	LIPE	lipase E, hormone sensitive type
16948	Lox	lysyl oxidase	4015	LOX	lysyl oxidase
17756	Map2	microtubule-associated protein 2	4133	MAP2	microtubule associated protein 2
17311	Kitl	kit ligand	4254	KITLG	KIT ligand
17386	Mmp13	matrix metallopeptidase 13	4322	MMP13	matrix metallopeptidase 13
17750	Mt2	metallothionein 2	4493	MT1E	metallothionein 1E
108156	Mthfd1	methylenetetrahydrofolate dehydrogenase (NADP+ dependent), methenyltetrahydrofolate cyclohydrolase, formyltetrahydrofolate synthase	4522	MTHFD1	methylenetetrahydrofolate dehydrogenase, cyclohydrolase and formyltetrahydrofolate synthetase 1
17888	Myh6	myosin, heavy polypeptide 6, cardiac muscle, alpha	4624	MYH6	myosin heavy chain 6
17897	Myl3	myosin, light polypeptide 3	4634	MYL3	myosin light chain 3
18534	Pck1	phosphoenolpyruvate carboxykinase 1, cytosolic	5105	PCK1	phosphoenolpyruvate carboxykinase 1
56492	Cldn18	claudin 18	51208	CLDN18	claudin 18
70676	Gulp1	GULP, engulfment adaptor PTB domain containing 1	51454	GULP1	GULP, engulfment adaptor PTB domain containing 1
27273	Pdk4	pyruvate dehydrogenase kinase, isoenzyme 4	5166	PDK4	pyruvate dehydrogenase kinase 4
56524	Mpp6	membrane protein, palmitoylated 6 (MAGUK p55 subfamily member 6)	51678	MPP6	membrane palmitoylated protein 6
192786	Rapgef6	Rap guanine nucleotide exchange factor (GEF) 6	51735	RAPGEF6	Rap guanine nucleotide exchange factor 6
18703	Pigr	polymeric immunoglobulin receptor	5284	PIGR	polymeric immunoglobulin receptor
18791	Plat	plasminogen activator, tissue	5327	PLAT	plasminogen activator, tissue type
239559	A4galt	alpha 1,4-galactosyltransferase	53947	A4GALT	alpha 1,4-galactosyltransferase
22589	Atrx	alpha thalassemia/mental retardation syndrome X-linked	546	ATRX	ATRX, chromatin remodeler
74393	Map10	microtubule-associated protein 10	54627	MAP10	microtubule associated protein 10

56194	Prpf40a	pre-mRNA processing factor 40A	55660	PRPF40A	pre-mRNA processing factor 40 homolog A
12069	Bex2	brain expressed X-linked 2	55859	BEX1	brain expressed X-linked 1
26414	Mapk10	mitogen-activated protein kinase 10	5602	MAPK10	mitogen-activated protein kinase 10
27981	Rsrp1	arginine/serine rich protein 1	57035	RSRP1	arginine and serine rich protein 1
57246	Tbx20	T-box 20	57057	TBX20	T-box 20
69368	Wdfy1	WD repeat and FYVE domain containing 1	57590	WDFY1	WD repeat and FYVE domain containing 1
17898	Myl7	myosin, light polypeptide 7, regulatory	58498	MYL7	myosin light chain 7
109263	Rlf	rearranged L-myc fusion sequence	6018	RLF	rearranged L-myc fusion
58198	Sall1	sal-like 1 (Drosophila)	6299	SALL1	spalt like transcription factor 1
94242	Tinagl1	tubulointerstitial nephritis antigen-like 1	64129	TINAGL1	tubulointerstitial nephritis antigen like 1
24053	Sgcg	sarcoglycan, gamma (dystrophin-associated glycoprotein)	6445	SGCG	sarcoglycan gamma
219257	Pcdh20	protocadherin 20	64881	PCDH20	protocadherin 20
57738	Slc15a2	solute carrier family 15 (H+/peptide transporter), member 2	6565	SLC15A2	solute carrier family 15 member 2
21672	Prdx2	peroxiredoxin 2	7001	PRDX2	peroxiredoxin 2
21685	Tef	thyrotroph embryonic factor	7008	TEF	TEF, PAR bZIP transcription factor
21835	Thrsp	thyroid hormone responsive	7069	THRSP	thyroid hormone responsive
22288	Utrn	utrophin	7402	UTRN	utrophin
12350	Car3	carbonic anhydrase 3	761	CA3	carbonic anhydrase 3
269593	Luzp1	leucine zipper protein 1	7798	LUZP1	leucine zipper protein 1
12310	Calca	calcitonin/calcitonin-related polypeptide, alpha	796	CALCA	calcitonin related polypeptide alpha
68169	Ndnf	neuron-derived neurotrophic factor	79625	NDNF	neuron derived neurotrophic factor
12310	Calca	calcitonin/calcitonin-related polypeptide, alpha	797	CALCB	calcitonin related polypeptide beta
77914	Krtap17-1	keratin associated protein 17-1	83902	KRTAP17-1	keratin associated protein 17-1
30785	Cttnbp2	cortactin binding protein 2	83992	CTTNBP2	cortactin binding protein 2
68802	Mypn	myopalladin	84665	MYPN	myopalladin
20361	Sema7a	sema domain, immunoglobulin domain (Ig), and GPI membrane anchor, (semaphorin) 7A	8482	SEMA7A	semaphorin 7A (John Milton Hagen blood group)

76574	Mfsd2a	major facilitator superfamily domain containing 2A	84879	MFSD2A	major facilitator superfamily domain containing 2A
93691	Klf7	Kruppel-like factor 7 (ubiquitous)	8609	KLF7	Kruppel like factor 7
21950	Tnfsf9	tumor necrosis factor (ligand) superfamily, member 9	8744	TNFSF9	tumor necrosis factor superfamily member 9
545156	Kalrn	kalirin, RhoGEF kinase	8997	KALRN	kalirin, RhoGEF kinase
53608	Map3k6	mitogen-activated protein kinase kinase kinase 6	9064	MAP3K6	mitogen-activated protein kinase kinase kinase 6
16826	Ldb2	LIM domain binding 2	9079	LDB2	LIM domain binding 2
12527	Cd9	CD9 antigen	928	CD9	CD9 molecule
93671	Cd163	CD163 antigen	9332	CD163	CD163 molecule
11450	Adipoq	adiponectin, C1Q and collagen domain containing	9370	ADIPOQ	adiponectin, C1Q and collagen domain containing
330119	Adamts3	a disintegrin-like and metallopeptidase (reprolysin type) with thrombospondin type 1 motif, 3	9508	ADAMTS3	ADAM metallopeptidase with thrombospondin type 1 motif 3
70297	Gcc2	GRIP and coiled-coil domain containing 2	9648	GCC2	GRIP and coiled-coil domain containing 2
83679	Pde4dip	phosphodiesterase 4D interacting protein (myomegalin)	9659	PDE4DIP	phosphodiesterase 4D interacting protein
12515	Cd69	CD69 antigen	969	CD69	CD69 molecule
72503	2610507B11Rik	RIKEN cDNA 2610507B11 gene	9703	KIAA0100	KIAA0100
212448	9330159F19Rik	RIKEN cDNA 9330159F19 gene	9729	KIAA0408	KIAA0408

Supplementary Table S5: Genes in Cd151KO and Cd9KO human orthologous sDEGs Lung Tissue filtered HCDP networks

Cd151KO Gene ID	Gene Symbol	Name
1012	CDH13	cadherin 13
1017	CDK2	cyclin dependent kinase 2
1019	CDK4	cyclin dependent kinase 4
1021	CDK6	cyclin dependent kinase 6
1026	CDKN1A	cyclin dependent kinase inhibitor 1A
1027	CDKN1B	cyclin dependent kinase inhibitor 1B
1029	CDKN2A	cyclin dependent kinase inhibitor 2A
10417	SPON2	spondin 2
10516	FBLN5	fibulin 5
11005	SPINK5	serine peptidase inhibitor, Kazal type 5
115426	UHRF2	ubiquitin like with PHD and ring finger domains 2
116362	RBP7	retinol binding protein 7
123169	LEO1	LEO1 homolog, Paf1/RNA polymerase II complex component
1272	CNTN1	contactin 1
1281	COL3A1	collagen type III alpha 1 chain
1407	CRY1	cryptochrome circadian clock 1
1408	CRY2	cryptochrome circadian clock 2
1410	CRYAB	crystallin alpha B
142	PARP1	poly(ADP-ribose) polymerase 1
1453	CSNK1D	casein kinase 1 delta
1454	CSNK1E	casein kinase 1 epsilon
1457	CSNK2A1	casein kinase 2 alpha 1
147372	CCBE1	collagen and calcium binding EGF domains 1
148523	CIART	circadian associated repressor of transcription
1600	DAB1	DAB1, reelin adaptor protein
1628	DBP	D-box binding PAR bZIP transcription factor

163183	SYNE4	spectrin repeat containing nuclear envelope family member 4
165679	SPTSSB	serine palmitoyltransferase small subunit B
166	AES	amino-terminal enhancer of split
1675	CFD	complement factor D
170689	ADAMTS15	ADAM metallopeptidase with thrombospondin type 1 motif 15
196	AHR	aryl hydrocarbon receptor
2006	ELN	elastin
2034	EPAS1	endothelial PAS domain protein 1
2064	ERBB2	erb-b2 receptor tyrosine kinase 2
2099	ESR1	estrogen receptor 1
2200	FBN1	fibrillin 1
2264	FGFR4	fibroblast growth factor receptor 4
23582	CCNDBP1	cyclin D1 binding protein 1
25849	PARM1	prostate androgen-regulated mucin-like protein 1
25902	MTHFD1L	methylenetetrahydrofolate dehydrogenase (NADP+ dependent) 1-like
26272	FBXO4	F-box protein 4
282974	STK32C	serine/threonine kinase 32C
288	ANK3	ankyrin 3
2885	GRB2	growth factor receptor bound protein 2
28986	MAGEH1	MAGE family member H1
30008	EFEMP2	EGF containing fibulin like extracellular matrix protein 2
3091	HIF1A	hypoxia inducible factor 1 alpha subunit
3131	HLF	HLF, PAR bZIP transcription factor
3161	HMMR	hyaluronan mediated motility receptor
3320	HSP90AA1	heat shock protein 90 alpha family class A member 1
3480	IGF1R	insulin like growth factor 1 receptor
3491	CYR61	cysteine rich angiogenic inducer 61
367	AR	androgen receptor

3845	KRAS	KRAS proto-oncogene, GTPase
4015	LOX	lysyl oxidase
4023	LPL	lipoprotein lipase
4046	LSP1	lymphocyte-specific protein 1
406	ARNTL	aryl hydrocarbon receptor nuclear translocator like
4087	SMAD2	SMAD family member 2
4089	SMAD4	SMAD family member 4
4321	MMP12	matrix metallopeptidase 12
4862	NPAS2	neuronal PAS domain protein 2
4897	NRCAM	neuronal cell adhesion molecule
5000	ORC4	origin recognition complex subunit 4
5111	PCNA	proliferating cell nuclear antigen
5187	PER1	period circadian clock 1
5290	PIK3CA	phosphatidylinositol-4,5-bisphosphate 3-kinase catalytic subunit alpha
5359	PLSCR1	phospholipid scramblase 1
54762	GRAMD1C	GRAM domain containing 1C
54828	BCAS3	BCAS3, microtubule associated cell migration factor
55165	CEP55	centrosomal protein 55
5519	PPP2R1B	protein phosphatase 2 scaffold subunit Abeta
55388	MCM10	minichromosome maintenance 10 replication initiation factor
5597	MAPK6	mitogen-activated protein kinase 6
5650	KLK7	kallikrein related peptidase 7
57057	TBX20	T-box 20
5787	PTPRB	protein tyrosine phosphatase, receptor type B
5803	PTPRZ1	protein tyrosine phosphatase, receptor type Z1
58538	MPP4	membrane palmitoylated protein 4
5925	RB1	RB transcriptional corepressor 1
595	CCND1	cyclin D1
5966	REL	REL proto-oncogene, NF-kB subunit

6678	SPARC	secreted protein acidic and cysteine rich
672	BRCA1	BRCA1, DNA repair associated
6774	STAT3	signal transducer and activator of transcription 3
6790	AURKA	aurora kinase A
6917	TCEA1	transcription elongation factor A1
7008	TEF	TEF, PAR bZIP transcription factor
7040	TGFB1	transforming growth factor beta 1
7069	THRSP	thyroid hormone responsive
7106	TSPAN4	tetraspanin 4
7251	TSG101	tumor susceptibility 101
7490	WT1	Wilms tumor 1
780	DDR1	discoidin domain receptor tyrosine kinase 1
79616	CCNJL	cyclin J like
80313	LRRC27	leucine rich repeat containing 27
8076	MFAP5	microfibrillar associated protein 5
8412	BCAR3	breast cancer anti-estrogen resistance 3
84172	POLR1B	RNA polymerase I subunit B
84879	MFSD2A	major facilitator superfamily domain containing 2A
8676	STX11	syntaxin 11
868	CBLB	Cbl proto-oncogene B
8863	PER3	period circadian clock 3
8864	PER2	period circadian clock 2
9370	ADIPOQ	adiponectin, C1Q and collagen domain containing
9530	BAG4	BCL2 associated athanogene 4
9575	CLOCK	clock circadian regulator
977	CD151	CD151 molecule (Raph blood group)
9821	RB1CC1	RB1 inducible coiled-coil 1

Cd9KO

Gene ID	Gene Symbol	Name
100	ADA	adenosine deaminase
10016	PDCD6	programmed cell death 6

10097	ACTR2	ARP2 actin related protein 2 homolog
10154	PLXNC1	plexin C1
10181	RBM5	RNA binding motif protein 5
1021	CDK6	cyclin dependent kinase 6
10234	LRRC17	leucine rich repeat containing 17
10241	CALCOCO2	calcium binding and coiled-coil domain 2
10262	SF3B4	splicing factor 3b subunit 4
10291	SF3A1	splicing factor 3a subunit 1
10302	SNAPC5	small nuclear RNA activating complex polypeptide 5
10320	IKZF1	IKAROS family zinc finger 1
10345	TRDN	triadin
10419	PRMT5	protein arginine methyltransferase 5
10456	HAX1	HCLS1 associated protein X-1
1051	CEBPB	CCAAT/enhancer binding protein beta
10516	FBLN5	fibulin 5
1054	CEBPG	CCAAT/enhancer binding protein gamma
10592	SMC2	structural maintenance of chromosomes 2
10813	UTP14A	UTP14A small subunit processome component
10964	IFI44L	interferon induced protein 44 like
10992	SF3B2	splicing factor 3b subunit 2
11007	CCDC85B	coiled-coil domain containing 85B
11030	RBPMS	RNA binding protein with multiple splicing
1106	CHD2	chromodomain helicase DNA binding protein 2
11100	HNRNPUL1	heterogeneous nuclear ribonucleoprotein U like 1
11124	FAF1	Fas associated factor 1
11140	CDC37	cell division cycle 37
11161	C14orf1	chromosome 14 open reading frame 1
11170	FAM107A	family with sequence similarity 107 member A
11228	RASSF8	Ras association domain family member 8
11244	ZHX1	zinc fingers and homeoboxes 1
113201	CASC4	cancer susceptibility candidate 4
11338	U2AF2	U2 small nuclear RNA auxiliary factor 2

114818	KLHL29	kelch like family member 29
115106	HAUS1	HAUS augmin like complex subunit 1
115572	FAM46B	family with sequence similarity 46 member B
116835	HSPA12B	heat shock protein family A (Hsp70) member 12B
116844	LRG1	leucine rich alpha-2-glycoprotein 1
116984	ARAP2	ArfGAP with RhoGAP domain, ankyrin repeat and PH domain 2
1191	CLU	clusterin
1196	CLK2	CDC like kinase 2
122786	FRMD6	FERM domain containing 6
124935	SLC43A2	solute carrier family 43 member 2
125488	TTC39C	tetratricopeptide repeat domain 39C
130340	AP1S3	adaptor related protein complex 1 sigma 3 subunit
130576	LYPD6B	LY6/PLAUR domain containing 6B
133746	JMY	junction mediating and regulatory protein, p53 cofactor
1387	CREBBP	CREB binding protein
140545	RNF32	ring finger protein 32
142	PARP1	poly(ADP-ribose) polymerase 1
1457	CSNK2A1	casein kinase 2 alpha 1
1459	CSNK2A2	casein kinase 2 alpha 2
147700	KLC3	kinesin light chain 3
147744	TMEM190	transmembrane protein 190
150921	TCF23	transcription factor 23
1571	CYP2E1	cytochrome P450 family 2 subfamily E member 1
1605	DAG1	dystroglycan 1
163	AP2B1	adaptor related protein complex 2 beta 1 subunit
163183	SYNE4	spectrin repeat containing nuclear envelope family member 4
1647	GADD45A	growth arrest and DNA damage inducible alpha

1649	DDIT3	DNA damage inducible transcript 3
1654	DDX3X	DEAD-box helicase 3, X-linked
1656	DDX6	DEAD-box helicase 6
165679	SPTSSB	serine palmitoyltransferase small subunit B
166	AES	amino-terminal enhancer of split
1665	DHX15	DEAH-box helicase 15
170689	ADAMTS15	ADAM metallopeptidase with thrombospondin type 1 motif 15
170954	PPP1R18	protein phosphatase 1 regulatory subunit 18
1729	DIAPH1	diaphanous related formin 1
1837	DTNA	dystrobrevin alpha
1892	ECHS1	enoyl-CoA hydratase, short chain 1
1910	EDNRB	endothelin receptor type B
1942	EFNA1	ephrin A1
1946	EFNA5	ephrin A5
1948	EFNB2	ephrin B2
1949	EFNB3	ephrin B3
1973	EIF4A1	eukaryotic translation initiation factor 4A1
1977	EIF4E	eukaryotic translation initiation factor 4E
1981	EIF4G1	eukaryotic translation initiation factor 4 gamma 1
2	A2M	alpha-2-macroglobulin
2001	ELF5	E74 like ETS transcription factor 5
2006	ELN	elastin
2022	ENG	endoglin
2043	EPHA4	EPH receptor A4
2064	ERBB2	erb-b2 receptor tyrosine kinase 2
2147	F2	coagulation factor II, thrombin
2167	FABP4	fatty acid binding protein 4
2186	BPTF	bromodomain PHD finger transcription factor
2189	FANCG	Fanconi anemia complementation group G
2200	FBN1	fibrillin 1
222389	BEND7	BEN domain containing 7
2243	FGA	fibrinogen alpha chain

2263	FGFR2	fibroblast growth factor receptor 2
2264	FGFR4	fibroblast growth factor receptor 4
2266	FGG	fibrinogen gamma chain
2289	FKBP5	FK506 binding protein 5
22919	MAPRE1	microtubule associated protein RP/EB family member 1
22981	NINL	ninein like
23002	DAAM1	dishevelled associated activator of morphogenesis 1
23023	TMCC1	transmembrane and coiled-coil domain family 1
2316	FLNA	filamin A
23256	SCFD1	sec1 family domain containing 1
23263	MCF2L	MCF.2 cell line derived transforming sequence like
23326	USP22	ubiquitin specific peptidase 22
23350	U2SURP	U2 snRNP associated SURP domain containing
23390	ZDHHC17	zinc finger DHHC-type containing 17
23468	CBX5	chromobox 5
23517	SKIV2L2	Ski2 like RNA helicase 2
2353	FOS	Fos proto-oncogene, AP-1 transcription factor subunit
2355	FOSL2	FOS like 2, AP-1 transcription factor subunit
23641	LDOC1	leucine zipper down-regulated in cancer 1
2495	FTH1	ferritin heavy chain 1
2534	FYN	FYN proto-oncogene, Src family tyrosine kinase
253461	ZBTB38	zinc finger and BTB domain containing 38
259	AMBP	alpha-1-microglobulin/bikunin precursor
25924	MYRIP	myosin VIIA and Rab interacting protein
26121	PRPF31	pre-mRNA processing factor 31
26353	HSPB8	heat shock protein family B (small) member 8
26986	PABPC1	poly(A) binding protein cytoplasmic 1
27106	ARRDC2	arrestin domain containing 2
27111	SDCBP2	syndecan binding protein 2

27129	HSPB7	heat shock protein family B (small) member 7
27161	AGO2	argonaute 2, RISC catalytic component
27289	RND1	Rho family GTPase 1
2729	GCLC	glutamate-cysteine ligase catalytic subunit
2730	GCLM	glutamate-cysteine ligase modifier subunit
27339	PRPF19	pre-mRNA processing factor 19
2740	GLP1R	glucagon like peptide 1 receptor
27443	CECR2	CECR2, histone acetyl-lysine reader
2764	GMFB	glia maturation factor beta
2801	GOLGA2	golgin A2
2810	SFN	stratifin
283078	MKX	mohawk homeobox
283152	CCDC153	coiled-coil domain containing 153
2885	GRB2	growth factor receptor bound protein 2
2888	GRB14	growth factor receptor bound protein 14
2892	GRIA3	glutamate ionotropic receptor AMPA type subunit 3
28986	MAGEH1	MAGE family member H1
2975	GTF3C1	general transcription factor IIIC subunit 1
29911	HOOK2	hook microtubule tethering protein 2
29984	RHOD	ras homolog family member D
30008	EFEMP2	EGF containing fibulin like extracellular matrix protein 2
3020	H3F3A	H3 histone family member 3A
3161	HMMR	hyaluronan mediated motility receptor
3183	HNRNPC	heterogeneous nuclear ribonucleoprotein C (C1/C2)
3184	HNRNPD	heterogeneous nuclear ribonucleoprotein D
3308	HSPA4	heat shock protein family A (Hsp70) member 4
3312	HSPA8	heat shock protein family A (Hsp70) member 8
3315	HSPB1	heat shock protein family B (small) member 1
3320	HSP90AA1	heat shock protein 90 alpha family class A member 1

3326	HSP90AB1	heat shock protein 90 alpha family class B member 1
3337	DNAJB1	DnaJ heat shock protein family (Hsp40) member B1
340527	NHSL2	NHS like 2
3480	IGF1R	insulin like growth factor 1 receptor
355	FAS	Fas cell surface death receptor
3725	JUN	Jun proto-oncogene, AP-1 transcription factor subunit
3726	JUNB	JunB proto-oncogene, AP-1 transcription factor subunit
3727	JUND	JunD proto-oncogene, AP-1 transcription factor subunit
3800	KIF5C	kinesin family member 5C
3815	KIT	KIT proto-oncogene receptor tyrosine kinase
3845	KRAS	KRAS proto-oncogene, GTPase
3866	KRT15	keratin 15
387	RHOA	ras homolog family member A
3875	KRT18	keratin 18
3880	KRT19	keratin 19
389075	RESP18	regulated endocrine specific protein 18
389432	SAMD5	sterile alpha motif domain containing 5
3932	LCK	LCK proto-oncogene, Src family tyrosine kinase
3939	LDHA	lactate dehydrogenase A
3991	LIPE	lipase E, hormone sensitive type
4005	LMO2	LIM domain only 2
4015	LOX	lysyl oxidase
4035	LRP1	LDL receptor related protein 1
4046	LSP1	lymphocyte-specific protein 1
408	ARRB1	arrestin beta 1
4089	SMAD4	SMAD family member 4
409	ARRB2	arrestin beta 2
4133	MAP2	microtubule associated protein 2

4150	MAZ	MYC associated zinc finger protein
4217	MAP3K5	mitogen-activated protein kinase kinase kinase 5
4254	KITLG	KIT ligand
4321	MMP12	matrix metallopeptidase 12
4322	MMP13	matrix metallopeptidase 13
4493	MT1E	metallothionein 1E
4522	MTHFD1	methylenetetrahydrofolate dehydrogenase, cyclohydrolase and formyltetrahydrofolate synthetase 1
4591	TRIM37	tripartite motif containing 37
4624	MYH6	myosin heavy chain 6
4634	MYL3	myosin light chain 3
467	ATF3	activating transcription factor 3
468	ATF4	activating transcription factor 4
4692	NDN	necdin, MAGE family member
4703	NEB	nebulin
4747	NEFL	neurofilament, light polypeptide
4790	NFKB1	nuclear factor kappa B subunit 1
4841	NONO	non-POU domain containing, octamer-binding
5054	SERPINE1	serpin family E member 1
51003	MED31	mediator complex subunit 31
5105	PCK1	phosphoenolpyruvate carboxykinase 1
51082	POLR1D	RNA polymerase I subunit D
51132	RLIM	ring finger protein, LIM domain interacting
51208	CLDN18	claudin 18
51421	AMOTL2	angiomotin like 2
51454	GULP1	GULP, engulfment adaptor PTB domain containing 1
5166	PDK4	pyruvate dehydrogenase kinase 4
51678	MPP6	membrane palmitoylated protein 6
51735	RAPGEF6	Rap guanine nucleotide exchange factor 6
51755	CDK12	cyclin dependent kinase 12
5272	SERPINB9	serpin family B member 9

5284	PIGR	polymeric immunoglobulin receptor
5290	PIK3CA	phosphatidylinositol-4,5-bisphosphate 3-kinase catalytic subunit alpha
5327	PLAT	plasminogen activator, tissue type
533	ATP6V0B	ATPase H+ transporting V0 subunit b
5364	PLXNB1	plexin B1
53947	A4GALT	alpha 1,4-galactosyltransferase
54462	CCSER2	coiled-coil serine rich protein 2
5447	POR	cytochrome p450 oxidoreductase
54536	EXOC6	exocyst complex component 6
546	ATRX	ATRX, chromatin remodeler
54627	MAP10	microtubule associated protein 10
54927	CHCHD3	coiled-coil-helix-coiled-coil-helix domain containing 3
55048	VPS37C	VPS37C, ESCRT-I subunit
55170	PRMT6	protein arginine methyltransferase 6
55195	C14orf105	chromosome 14 open reading frame 105
55337	C19orf66	chromosome 19 open reading frame 66
554313	HIST2H4B	histone cluster 2 H4 family member b
55660	PRPF40A	pre-mRNA processing factor 40 homolog A
55802	DCP1A	decapping mRNA 1A
55859	BEX1	brain expressed X-linked 1
55968	NSFL1C	NSFL1 cofactor
5602	MAPK10	mitogen-activated protein kinase 10
5621	PRNP	prion protein
57035	RSRP1	arginine and serine rich protein 1
57057	TBX20	T-box 20
5710	PSMD4	proteasome 26S subunit, non-ATPase 4
572	BAD	BCL2 associated agonist of cell death
5728	PTEN	phosphatase and tensin homolog
57409	MIF4GD	MIF4G domain containing
57562	CEP126	centrosomal protein 126
57590	WDFY1	WD repeat and FYVE domain containing 1

57639	CCDC146	coiled-coil domain containing 146
5764	PTN	pleiotrophin
57761	TRIB3	tribbles pseudokinase 3
5783	PTPN13	protein tyrosine phosphatase, non-receptor type 13
58498	MYL7	myosin light chain 7
5879	RAC1	ras-related C3 botulinum toxin substrate 1 (rho family, small GTP binding protein Rac1)
5888	RAD51	RAD51 recombinase
5906	RAP1A	RAP1A, member of RAS oncogene family
5936	RBM4	RNA binding motif protein 4
5966	REL	REL proto-oncogene, NF-kB subunit
5970	RELA	RELA proto-oncogene, NF-kB subunit
5987	TRIM27	tripartite motif containing 27
6016	RIT1	Ras like without CAAX 1
6018	RLF	rearranged L-myc fusion
6210	RPS15A	ribosomal protein S15a
6261	RYR1	ryanodine receptor 1
6299	SALL1	spalt like transcription factor 1
6310	ATXN1	ataxin 1
6386	SDCBP	syndecan binding protein
64129	TINAGL1	tubulointerstitial nephritis antigen like 1
64170	CARD9	caspase recruitment domain family member 9
6444	SGCD	sarcoglycan delta
6445	SGCG	sarcoglycan gamma
64764	CREB3L2	cAMP responsive element binding protein 3 like 2
64881	PCDH20	protocadherin 20
653121	ZBTB8A	zinc finger and BTB domain containing 8A
6565	SLC15A2	solute carrier family 15 member 2
6625	SNRNP70	small nuclear ribonucleoprotein U1 subunit 70
6645	SNTB2	syntrophin beta 2
6660	SOX5	SRY-box 5

6732	SRPK1	SRSF protein kinase 1
6733	SRPK2	SRSF protein kinase 2
6790	AURKA	aurora kinase A
6925	TCF4	transcription factor 4
7001	PRDX2	peroxiredoxin 2
7008	TEF	TEF, PAR bZIP transcription factor
7040	TGFB1	transforming growth factor beta 1
7046	TGFBR1	transforming growth factor beta receptor 1
7048	TGFBR2	transforming growth factor beta receptor 2
7069	THRSP	thyroid hormone responsive
7251	TSG101	tumor susceptibility 101
7329	UBE2I	ubiquitin conjugating enzyme E2 I
7341	SUMO1	small ubiquitin-like modifier 1
7402	UTRN	utrophin
7431	VIM	vimentin
7490	WT1	Wilms tumor 1
7532	YWHAG	tyrosine 3-monooxygenase/tryptophan 5-monooxygenase activation protein gamma
7534	YWHAZ	tyrosine 3-monooxygenase/tryptophan 5-monooxygenase activation protein zeta
7536	SF1	splicing factor 1
7572	ZNF24	zinc finger protein 24
761	CA3	carbonic anhydrase 3
762	CA4	carbonic anhydrase 4
7798	LUZP1	leucine zipper protein 1
7846	TUBA1A	tubulin alpha 1a
79038	ZFYVE21	zinc finger FYVE-type containing 21
796	CALCA	calcitonin related polypeptide alpha
79625	NDNF	neuron derived neurotrophic factor
79666	PLEKHF2	pleckstrin homology and FYVE domain containing 2
797	CALCB	calcitonin related polypeptide beta
79719	AAGAB	alpha- and gamma-adaptin binding protein

79892	MCMBP	minichromosome maintenance complex binding protein
79959	CEP76	centrosomal protein 76
80184	CEP290	centrosomal protein 290
80321	CEP70	centrosomal protein 70
80351	TNKS2	tankyrase 2
83659	TEKT1	tektin 1
83902	KRTAP17-1	keratin associated protein 17-1
83992	CTTNBP2	cortactin binding protein 2
8405	SPOP	speckle type BTB/POZ protein
841	CASP8	caspase 8
8412	BCAR3	breast cancer anti-estrogen resistance 3
84140	FAM161A	family with sequence similarity 161 member A
84665	MYPN	myopalladin
8482	SEMA7A	semaphorin 7A (John Milton Hagen blood group)
84879	MFSD2A	major facilitator superfamily domain containing 2A
8543	LMO4	LIM domain only 4
8569	MKNK1	MAP kinase interacting serine/threonine kinase 1
8609	KLF7	Kruppel like factor 7
8615	USO1	USO1 vesicle transport factor
8737	RIPK1	receptor interacting serine/threonine kinase 1
8744	TNFSF9	tumor necrosis factor superfamily member 9
8825	LIN7A	lin-7 homolog A, crumbs cell polarity complex component
8930	MBD4	methyl-CpG binding domain 4, DNA glycosylase
896	CCND3	cyclin D3
8997	KALRN	kalirin, RhoGEF kinase
9064	MAP3K6	mitogen-activated protein kinase kinase kinase 6
9079	LDB2	LIM domain binding 2
91010	FMNL3	formin like 3
9240	PNMA1	paraneoplastic Ma antigen 1
928	CD9	CD9 molecule

9319	TRIP13	thyroid hormone receptor interactor 13
9332	CD163	CD163 molecule
9370	ADIPOQ	adiponectin, C1Q and collagen domain containing
9508	ADAMTS3	ADAM metallopeptidase with thrombospondin type 1 motif 3
9530	BAG4	BCL2 associated athanogene 4
9584	RBM39	RNA binding motif protein 39
9627	SNCAIP	synuclein alpha interacting protein
9648	GCC2	GRIP and coiled-coil domain containing 2
9659	PDE4DIP	phosphodiesterase 4D interacting protein
969	CD69	CD69 molecule
9703	KIAA0100	KIAA0100
9729	KIAA0408	KIAA0408
9802	DAZAP2	DAZ associated protein 2
9821	RB1CC1	RB1 inducible coiled-coil 1
9879	DDX46	DEAD-box helicase 46
998	CDC42	cell division cycle 42
9993	DGCR2	DiGeorge syndrome critical region gene 2

Supplementary Table S6: Cd151KO and Cd9KO human orthologous sDEGs Lung Tissue filtered HCDP networks

Cd151KO

Gene 1	Gene 2
LSP1	THRSP
CDK6	UHRF2
CCND1	CDKN1B
CNTN1	PTPRZ1
CDK6	RB1
AURKA	THRSP
CDKN2A	MCM10
AR	BRCA1
CSNK2A1	LEO1
HIF1A	HSP90AA1
FBN1	LOX
PCNA	PCNA
CCND1	FBXO4
CEP55	TSG101
ERBB2	THRSP
CDK4	UHRF2
CRY2	PER2
CDH13	HMMR
CCND1	MCM10
CRY1	PLSCR1
RB1	RB1
BRCA1	ESR1
THRSP	TSG101
CCND1	LPL
ARNTL	NPAS2
CDKN1A	ESR1
AR	STAT3
BCAS3	SMAD4

CCND1	STAT3
AR	HSP90AA1
CRY1	PER2
CDK6	DAB1
CCBE1	CEP55
CRY1	PPP2R1B
AES	STX11
SMAD4	THRSP
AR	RB1
CBLB	CRY1
CDK2	PCNA
AR	TSG101
CDK2	RB1
CNTN1	PTPRB
CCND1	KLK7
CD151	GRAMD1C
MCM10	MCM10
CDK4	CDKN1B
ESR1	SMAD2
ELN	MMP12
CRY1	PER1
BCAS3	TGFB1
BRCA1	CDK4
COL3A1	SPARC
BCAS3	CCND1
BCAR3	ERBB2
ARNTL	CRY1
GRB2	REL
CDKN1A	PCNA
ELN	FBN1
CCND1	RB1
DAB1	ERBB2
CSNK2A1	HSP90AA1

FGFR4	THRSP
AES	CCNJL
THRSP	WT1
AURKA	HMMR
AURKA	BRCA1
CDK6	CDKN1A
EFEMP2	LOX
GRB2	GRB2
SMAD2	SMAD4
ERBB2	STAT3
CCND1	CRYAB
CEP55	CEP55
AES	REL
IGF1R	IGF1R
BCAS3	KRAS
CDKN1B	GRB2
CDK2	UHRF2
ERBB2	GRB2
ARNTL	HIF1A
CIART	DAB1
CSNK1D	SMAD2
PARP1	PCNA
KRAS	THRSP
BCAR3	THRSP
CDK4	CDKN2A
CDK4	RB1
RB1	UHRF2
ELN	FBLN5
CDK6	MCM10
EFEMP2	PLSCR1
PARP1	THRSP
LEO1	TCEA1
AHR	ARNTL

BAG4	THRSP
CBLB	GRB2
AR	CCND1
BRCA1	BRCA1
HMMR	THRSP
CCND1	ORC4
AES	GRB2
ARNTL	CSNK1E
ARNTL	HSP90AA1
CDK2	CDKN1A
CDKN2A	PCNA
ANK3	SMAD2
CCND1	CCNDBP1
CDKN1A	MCM10
BAG4	KLK7
CCND1	CDKN1A
CDK6	CDKN2A
EFEMP2	ELN
ESR1	HSP90AA1
HSP90AA1	NPAS2
CCND1	CDK2
KLK7	TGFB1
CCND1	POLR1B
CDK2	CDKN1B
CRY2	PER1
BRCA1	CDK2
EFEMP2	FBLN5
ADIPOQ	SYNE4
RB1CC1	THRSP
AES	AES
CDKN1A	PARP1
TGFB1	TGFB1
STX11	STX11

ARNTL	CLOCK
BCAS3	TSG101
AHR	HSP90AA1
PIK3CA	THRSP
CSNK1E	PER2
ELN	MAGEH1
ELN	LOX
PARP1	PARP1
GRB2	STK32C
TGFB1	THRSP
CRY1	CSNK1E
CSNK1D	PER2
COL3A1	DDR1
AURKA	CDH13
CDK4	CDKN1A
CCND1	CDK6
CCND1	ESR1
ANK3	MAPK6
THRSP	THRSP
IGF1R	THRSP
CDKN2A	ORC4
HSP90AA1	HSP90AA1
CCND1	CDKN2A
CCND1	CDH13
MCM10	ORC4
FBN1	FBN1
BRCA1	RB1
KLK7	WT1
ARNTL	EPAS1
CSNK1E	PER1
PCNA	UHRF2
EFEMP2	FBN1
BRCA1	CCND1

CCND1	UHRF2
BAG4	BCAS3
CRYAB	CRYAB
CCND1	CDK4
CCND1	PCNA
CCBE1	STX11
AHR	RB1
CCNJL	REL

Cd9KO

**Gene 1**	**Gene 2**
CALCOCO2	LMO4
PRMT5	YWHAZ
NONO	PLEKHF2
ATF4	DDIT3
HOOK2	UTP14A
AURKA	THRSP
KRT15	LDOC1
CSNK2A1	HSP90AB1
DDX6	NONO
RBPMS	VPS37C
GOLGA2	PPP1R18
PARP1	RELA
HAUS1	KRT15
CLK2	CLK2
CARD9	CARD9
PRMT5	ZDHHC17
CEP70	FAM161A
HSPB7	SF3B4
ERBB2	THRSP
JUNB	MAP2
KIAA0100	REL
EIF4A1	RBM4

PRNP	PRNP
HNRNPD	PABPC1
DDX3X	PABPC1
UTP14A	VIM
FAM107A	KRT19
FTH1	HSPB1
RAD51	ZDHHC17
PDE4DIP	PPP1R18
MED31	SERPINB9
DDIT3	FOSL2
JUN	MAPK10
CCDC146	PDE4DIP
CCDC146	KRT19
SDCBP	TRIM27
RELA	REL
HAUS1	PDE4DIP
HSPB1	KRT18
BPTF	MAZ
DCP1A	DCP1A
PNMA1	SDCBP
TRIM27	UBE2I
ATRX	CBX5
KRAS	RBPMS
FAS	PDCD6
DDIT3	FOS
CEP70	CEP70
VIM	ZHX1
C14orf1	HNRNPUL1
FAS	HNRNPC
C14orf105	PNMA1
CEP76	HSPB7
AURKA	SRPK2
GOLGA2	HAUS1

LMO2	ZNF24
CSNK2A1	CSNK2A2
C19orf66	TCF4
BEND7	DDX6
ARRB2	CDC42
FTH1	SDCBP
GRB2	REL
PLEKHF2	SFN
KRT15	TSG101
KIAA0408	YWHAG
JUNB	NINL
FMNL3	PRPF40A
CYP2E1	ECHS1
FOS	JUN
RELA	SNRNP70
MAP3K5	MAP3K5
CREBBP	CSNK2A1
FAM107A	NINL
CCDC85B	LDOC1
AMBP	DAAM1
SNTB2	UTRN
DDIT3	F2
CDC37	CDK6
IGF1R	IGF1R
EFEMP2	FAM107A
ATF4	JUN
KIAA0408	SFN
ERBB2	SRPK1
AES	VPS37C
DAZAP2	VPS37C
CHCHD3	TRIM27
HSPB7	REL
LCK	LCK

FOSL2	JUN
SCFD1	USO1
HSP90AA1	TGFBR1
CLK2	SNRNP70
ATXN1	YWHAZ
KRT15	PRPF31
DDX6	IKZF1
DAAM1	DAAM1
AES	U2AF2
CHD2	TEKT1
BEX1	CASP8
PRMT6	RAC1
CREBBP	MAP3K5
BAG4	THRSP
GOLGA2	USO1
PRPF40A	SF1
HMMR	THRSP
AMOTL2	LMO4
AES	GRB2
AAGAB	AP1S3
AP2B1	ARRB2
DDIT3	TRIB3
CALCOCO2	FAM107A
EIF4G1	MKNK1
CARD9	SUMO1
ATF4	FOS
GRB2	KIT
CDC42	MCF2L
NINL	UTP14A
ARRB1	MAP3K5
HSP90AB1	HSP90AB1
EFEMP2	ELN
CSNK2A1	PRPF40A

CEP70	HAUS1
PRPF19	U2AF2
HNRNPD	HSPA4
MCF2L	RAC1
DAG1	UTRN
RELA	TCF4
RB1CC1	THRSP
LDHA	MAPK10
HOOK2	KIAA0408
FAM46B	SOX5
KALRN	SNCAIP
KLC3	PDE4DIP
MAP2	TUBA1A
HNRNPD	HSPB1
BEND7	SDCBP2
PIK3CA	THRSP
GOLGA2	LMO2
CEP70	PRPF31
AES	SOX5
A2M	RNF32
ATF3	DDIT3
CEBPB	DDIT3
DNAJB1	HSPA8
HNRNPC	SRPK2
ELN	LOX
DAZAP2	RBPMS
PARP1	PARP1
TGFB1	THRSP
CREBBP	MKNK1
ATRX	PTN
CCDC85B	CEP70
CDC37	PRDX2
JUNB	SMAD4

SDCBP2	SDCBP2
TRIM27	TRIM27
LMO2	SOX5
AES	PRPF31
HSPB1	PRPF40A
LDB2	RLIM
FKBP5	HSP90AB1
RAD51	VIM
ATF3	ATF4
SNRNP70	SRPK2
HSP90AA1	JUN
CHCHD3	GOLGA2
DIAPH1	PRPF40A
FGFR2	FGFR2
CCDC85B	PLEKHF2
DAG1	GRB2
CLK2	TRIM27
FTH1	MYL3
TSG101	VPS37C
AES	LMO2
EFEMP2	RBPMS
CEP70	UTP14A
VIM	YWHAZ
EFNB2	EPHA4
CSNK2A1	ZHX1
HSP90AB1	MKNK1
LSP1	THRSP
HSPB1	RBM39
GOLGA2	KRT18
CCDC85B	FAM107A
JUND	MAPK10
FAM107A	VIM
AMOTL2	AMOTL2

TGFB1	TGFBR1
FBN1	LOX
CSNK2A1	HNRNPC
RBPMS	TRIP13
LMO2	RELA
AES	KRT15
CSNK2A1	SF3B2
KRT18	YWHAG
DAZAP2	LMO2
MAP3K5	MAP3K6
AES	CARD9
KRT15	NEFL
ATRX	CEP126
HSPB1	MED31
AMOTL2	KRT19
DDX46	SRPK2
ENG	TGFBR1
CD69	TMEM190
PRPF40A	SDCBP2
SMAD4	THRSP
ATF4	LDOC1
FAS	UBE2I
CARD9	KRT19
PDE4DIP	ZFYVE21
CSNK2A2	KIF5C
PDE4DIP	PRPF31
MAPRE1	PDE4DIP
CCDC85B	CHCHD3
EFNA1	EPHA4
HNRNPC	UBE2I
CALCOCO2	LMO2
ELN	MMP12
RBPMS	RBPMS

EFNA5	EPHA4
DDX6	ZNF24
GTF3C1	MAPK10
GOLGA2	ZDHHC17
ADA	SGCD
C14orf1	CDC42
SNRNP70	SRPK1
CCDC85B	KIAA0408
HSPA4	NFKB1
HSP90AA1	HSPB1
HAUS1	KRT18
KIAA0408	TRIM37
FAM161A	PDE4DIP
AES	TRIM27
GOLGA2	RBM39
NEFL	PPP1R18
KIT	LCK
AP2B1	TGFBR2
CCND3	CDK6
KIT	KITLG
FTH1	SOX5
CHD2	SRPK2
THRSP	WT1
EFEMP2	LOX
PRMT5	PRMT5
FAM107A	LDOC1
HSPB1	LUZP1
MPP6	SKIV2L2
LDOC1	SDCBP
AES	REL
HSPA8	PTEN
CHCHD3	PDE4DIP
FAS	SUMO1

LMO4	TCF4
FGA	PLAT
KRAS	THRSP
BCAR3	THRSP
LMO4	SMAD4
FAM107A	PNMA1
TRIP13	TRIP13
PDCD6	VPS37C
CCDC85B	UTP14A
CBX5	SRPK1
CYP2E1	POR
FAM161A	FAM161A
C19orf66	C19orf66
DAAM1	RHOA
KRT19	PPP1R18
ATF3	ATF3
HNRNPUL1	HNRNPUL1
GOLGA2	LIN7A
GULP1	SMC2
AES	LMO4
PTN	SNCAIP
KRT15	KRT19
RIT1	RLF
KRAS	ZDHHC17
FLNA	HSPB7
CEP290	MAPK10
PARP1	UBE2I
PRPF40A	SNRNP70
MBD4	TRIM27
BAD	YWHAZ
CREBBP	RELA
FAS	GADD45A
FAF1	FAS

CEP70	DDX6
HSPA4	RELA
SMAD4	UBE2I
CALCOCO2	SDCBP
FKBP5	HSP90AA1
FAS	LCK
KRT15	KRT18
PSMD4	RASSF8
ENG	TGFB1
PTPN13	RAPGEF6
KRT19	UBE2I
ELN	MAGEH1
DDX6	GOLGA2
LMO4	NONO
NEFL	PRMT6
TGFBR1	TGFBR2
SCFD1	USP22
HSPB7	TCF4
BEX1	PTEN
C14orf105	GOLGA2
ATXN1	FAM46B
NFKB1	PARP1
AES	GOLGA2
FAM107A	KRT15
CLU	CYP2E1
FOS	JUND
IGF1R	THRSP
LMO2	REL
DIAPH1	RHOA
AURKA	SRPK1
DDIT3	MCMBP
CALCOCO2	PPP1R18
FABP4	LIPE

SDCBP	ZBTB8A
BEX1	PIK3CA
ATXN1	ATXN1
FAM161A	ZBTB8A
PLXNB1	RND1
GRB2	LMO2
BPTF	H3F3A
CALCOCO2	DDX6
ZBTB8A	ZBTB8A
ATF3	RELA
CCND3	EFEMP2
REL	SDCBP
CALCOCO2	PRPF31
DHX15	RBM5
C14orf1	ZNF24
CDK6	SOX5
DDIT3	SNAPC5
EIF4G1	PABPC1
SF3B2	SF3B4
GRB2	VIM
CBX5	SRPK2
CREBBP	FOS
CSNK2A1	SNRNP70
LCK	PIK3CA
HNRNPC	SDCBP
GOLGA2	LMO4
UBE2I	ZBTB8A
GOLGA2	PRPF31
ATF4	TRIB3
CSNK2A1	DDX3X
GRB2	TRIM27
CARD9	FAM161A
CDK12	PRPF40A

PRPF40A	SDCBP
FAM161A	NINL
CBX5	H3F3A
DDX6	TRIM37
RAD51	SUMO1
CCND3	FAS
TINAGL1	UTP14A
DDX6	TRIM27
ATRX	RAD51
JUN	UBE2I
C19orf66	PDE4DIP
FABP4	VIM
BEX1	FGFR2
PRPF31	TRIM27
BCAR3	BEX1
HSP90AA1	TGFBR2
HNRNPC	LMO2
SDCBP	TEKT1
FGFR2	GRB2
SRPK2	SRPK2
UBE2I	ZHX1
CSNK2A1	PTEN
EIF4E	TRIM27
DDX6	ZBTB8A
CSNK2A1	KIF5C
USO1	USO1
FOS	UBE2I
CCDC146	GOLGA2
DAG1	FYN
KIAA0408	PRMT6
AES	LDOC1
KRT15	LMO4
PRMT5	RSRP1

BAG4	BEX1
AES	RBPMS
RYR1	TRDN
BAD	PRDX2
KIAA0408	ZDHHC17
RAD51	RAD51
DCP1A	SMAD4
AMOTL2	DDIT3
GRB2	SF3B4
MAPK10	RELA
DAZAP2	DAZAP2
IKZF1	SDCBP
PLXNC1	SEMA7A
FGFR4	THRSP
RSRP1	SRPK1
AURKA	HMMR
FOS	JUNB
FAS	MBD4
CALCOCO2	FAM161A
HSPB1	VIM
CCDC85B	HNRNPC
HNRNPD	SFN
A2M	PRDX2
TINAGL1	TRIP13
SRPK2	U2AF2
CEP126	ZNF24
CSNK2A1	JUN
PLAT	SERPINE1
HAUS1	LDOC1
CSNK2A1	UTP14A
HNRNPD	UBE2I
FAM161A	TRIM37
KRT18	PDE4DIP

CARD9	PPP1R18
HSPB7	PLEKHF2
EIF4E	EIF4G1
SF1	U2AF2
PRDX2	YWHAZ
RBM39	SF3B4
ATXN1	REL
HNRNPC	KRAS
KLC3	KLC3
HAUS1	HAUS1
CA4	PRDX2
MAPRE1	MAPRE1
CALCOCO2	CHCHD3
CEBPG	DDIT3
CCDC146	NINL
EXOC6	PRDX2
CSNK2A1	RELA
PDCD6	PDCD6
ARRB1	CDC42
FAM161A	IKZF1
FRMD6	IKZF1
GRB2	MAP2
RBM39	SDCBP
AMOTL2	CARD9
TGFB1	TGFBR2
PRPF19	RBM5
ATF4	CEBPG
PPP1R18	TCF4
RSRP1	SRPK2
VIM	VIM
PDE4DIP	PRNP
GOLGA2	VPS37C
MPP6	NFKB1

RBM5	U2AF2
BPTF	HIST2H4B
AGO2	DDX6
PRPF40A	U2AF2
HAX1	MAPK10
CARD9	KIAA0408
FLNA	REL
HSP90AB1	HSPB1
DAZAP2	FAM46B
EFEMP2	FBLN5
PRPF40A	SF3A1
BEND7	BEND7
ENG	TGFBR2
AES	AES
HAUS1	NINL
DCP1A	DDX6
PRDX2	RNF32
FANCG	KRT19
TGFB1	TGFB1
ACTR2	ELF5
FLNA	TCF4
HNRNPC	HNRNPC
SUMO1	UBE2I
HAUS1	KRT19
GOLGA2	UBE2I
CDK6	DDIT3
LRP1	PLAT
KIAA0408	KRT15
ATF4	CREBBP
ATP6V0B	YWHAG
ATXN1	CDK6
FRMD6	RASSF8
BEND7	PLEKHF2

CLK2	SDCBP
SOX5	SOX5
RBPMS	TINAGL1
HSPA4	HSPA8
RBM39	REL
ERBB2	FYN
FAF1	HSPA4
AES	TCF4
CALCA	CALCA
AES	CALCOCO2
ARRB1	JUN
DDX46	PRPF40A
AES	RELA
AES	DDX6
CEBPB	RELA
KLC3	KRT15
SNCAIP	SNCAIP
RELA	SMAD4
FAM107A	TRIM37
GRB14	TNKS2
AP2B1	KIAA0408
CALCOCO2	DAZAP2
BEX1	SMAD4
ATXN1	U2AF2
HIST2H4B	PRMT5
PTEN	UTP14A
EIF4G1	HNRNPD
CLK2	UBE2I
RBM39	SRPK2
RAD51	UBE2I
C14orf1	FAS
EFNB3	EPHA4
SDCBP	SDCBP

LDOC1	PLEKHF2
DDIT3	LMO2
CASP8	CASP8
IKZF1	IKZF1
LMO4	RBPMS
HNRNPUL1	VPS37C
PRPF40A	SERPINB9
DDX6	TCF4
CCDC153	DDIT3
THRSP	TSG101
HAUS1	TRIM27
FTH1	FTH1
HSP90AA1	RAD51
ATF3	JUN
ATF4	JUNB
CYP2E1	FANCG
EIF4G1	EIF4G1
DDIT3	SPOP
ENG	LRG1
PRPF40A	SF3B2
MYPN	NEB
HSPB1	YWHAZ
RBM5	U2SURP
NFKB1	NFKB1
KRT15	LMO2
RIPK1	RIPK1
PPP1R18	TRIM27
ARRB1	MAPK10
HNRNPUL1	PRPF40A
FAM107A	HOOK2
TMCC1	ZDHHC17
ELF5	RPS15A
HNRNPC	SUMO1

FAS	RAP1A
CLK2	RSRP1
CARD9	CCDC85B
DDX6	SRPK2
AMOTL2	GOLGA2
DDX3X	EIF4G1
SERPINB9	ZHX1
EIF4A1	EIF4G1
AMOTL2	TRIM27
LDOC1	PRPF31
CARD9	CEP70
KRT19	KRT19
FYN	PRMT6
NFKB1	RELA
CEP70	ZBTB8A
KIAA0408	VIM
BEND7	U2AF2
CREBBP	MAPK10
NONO	NONO
MPP6	NDN
AP2B1	HSPB1
CCDC146	PNMA1
BCAR3	ERBB2
KLC3	TCF4
TRIM27	ZFYVE21
LMO2	UBE2I
LDOC1	UTP14A
PTN	RIT1
RBM39	U2AF2
NEFL	PDE4DIP
ELN	FBN1
HSPA4	HSPB1
CLK2	SRPK2

ATRX	H3F3A
AMOTL2	KRT15
CSNK2A1	HSP90AA1
BAD	YWHAG
HAUS1	TSG101
FYN	MAP2
GRB2	GRB2
PABPC1	RBM4
HNRNPUL1	MED31
CDC37	DNAJB1
CCDC146	KRT18
ARRB2	MAP3K5
CASP8	RIPK1
BEND7	CHD2
ATXN1	ZHX1
C14orf105	PDE4DIP
DDIT3	DGCR2
DDIT3	VPS37C
ERBB2	GRB2
HNRNPUL1	SF3B4
ATRX	CCSER2
CSNK2A1	SF3A1
PRPF40A	ZHX1
CDC37	HSP90AA1
RBM39	SRPK1
CREB3L2	GULP1
GRB14	IGF1R
NEFL	VIM
C14orf1	SERPINB9
ELN	FBLN5
PARP1	THRSP
MED31	TINAGL1
AES	MED31

ATXN1	DAZAP2
PDE4DIP	ZBTB38
ATXN1	RBPMS
HSPA4	REL
AES	SDCBP
HNRNPD	U2AF2
IKZF1	UBE2I
RBM5	ZDHHC17
EFNB3	YWHAZ
LMO2	MAPRE1
CREBBP	PARP1
ARRB2	MAPK10
HSP90AB1	PTEN
HNRNPC	HNRNPD
LMO4	PDE4DIP
PRMT5	TRIB3
CASP8	VIM
UTP14A	ZNF24
DAG1	DAG1
PDE4DIP	SDCBP
CEP126	YWHAZ
DAAM1	RHOD
CEBPB	SMAD4
ADIPOQ	SYNE4
BAG4	HSPA8
BAD	SFN
CLK2	RBM39
RIT1	SRPK2
LDB2	LMO4
RBPMS	SF1
PNMA1	PRPF31
C14orf105	TRIM27
LDOC1	LDOC1

CDC37	RNF32
KRT18	KRT18
CLU	FOS
MIF4GD	PDE4DIP
PRPF40A	RBM39
ATP6V0B	FAS
C19orf66	IKZF1
THRSP	THRSP
ARRB2	CSNK2A1
GCLC	GCLM
HSPB7	HSPB8
AES	SRPK2
CCDC85B	KRT18
HSP90AA1	HSP90AA1
HSPB1	HSPB8
H3F3A	PARP1
FBN1	FBN1
ATXN1	EFEMP2
DDIT3	JUN
EFEMP2	FBN1
PTEN	SPOP
DNAJB1	HSPA4
KRT15	PPP1R18
FAS	RIPK1
LIPE	NSFL1C
HSPA8	JUN
KRT18	LDOC1
CREBBP	JUN
LIN7A	MPP6
DDIT3	POLR1D
EIF4G1	RBM4
HSPB1	TTC39C
DIAPH1	RHOD

DTNA UTRN

Supplementary Table S7: Human orthologoues of Mouse sDEGs that were identified as Bottlenecks and Hubs in Cd151KO and Cd9KO human orthologous sDEGs Lung Tissue filtered HCDP networks

Cd151KO

Gene ID	Gene Symbol	Name	Bottleneck	Hub
595	CCND1	cyclin D1	Yes	Yes
7069	THRSP	thyroid hormone responsive	No	Yes

Cd9KO

Gene ID	Gene Symbol	Name	Bottleneck	Hub
11170	FAM107A	family with sequence similarity 107 member A	No	Yes
1649	DDIT3	DNA damage inducible transcript 3	Yes	Yes
1656	DDX6	DEAD-box helicase 6	Yes	Yes
355	FAS	Fas cell surface death receptor	Yes	Yes
55660	PRPF40A	pre-mRNA processing factor 40 homolog A	Yes	Yes
5602	MAPK10	mitogen-activated protein kinase 10	Yes	No
7069	THRSP	thyroid hormone responsive	No	Yes
9659	PDE4DIP	phosphodiesterase 4D interacting protein	Yes	Yes
9729	KIAA0408	KIAA0408	Yes	Yes

Supplementary Table S8A: Cd151KO and Cd9KO human orthologous DEG Lung Tissue filtered HCDP networks- Enriched KEGG pathway associations (*p* ≤0.005)

Cd151KO

Identifier	Name	Foreground (in the list)	FG%	Background (whole genome)	BG%	p-value	Associated gene ids
hsa05200	Pathways in cancer	22	34.92063	397	5.56802244	7.03E-11	367, 595, 868, 1017, 1019, 1021, 1026, 1027, 1029, 2034, 2064, 2885, 3091, 3320, 3480, 3845, 4087, 4089, 5290, 5925, 6774
hsa05161	Hepatitis B	14	22.22222	146	2.047685835	9.85E-10	595, 1017, 1019, 1021, 1026, 1027, 2885, [illegible], 4089, 5111, 5290, 5925, 6774, 7040
hsa05205	Proteoglycans in cancer	14	22.22222	205	2.875175316	4.68E-08	288, 595, 868, 1026, 2064, 2099, 2885, [illegible], 3480, 3845, [illegible], 5290, 6774, 7040
hsa04151	PI3K-Akt signaling pathway	14	22.22222	341	4.782608696	1.89E-05	595, 672, 1017, 1019, 1021, 1026, 1027, [illegible], 2885, 3320, 3480, 3845, 5290, [illegible]
hsa05220	Chronic myeloid leukemia	13	20.63492	73	1.023842917	8.11E-12	595, 868, 1019, 1021, 1026, 1027, 1029, 2885, 3845, 4089, 5290, 5925, 7040
hsa04110	Cell cycle	13	20.63492	124	1.739130435	1.36E-09	595, 1017, 1019, 1021, 1026, 1027, 1029, [illegible], 4089, [illegible], 5111, 5925, 7040
hsa04068	FoxO signaling pathway	13	20.63492	134	1.879382889	3.26E-09	595, 1017, 1026, 1027, 1454, 2885, 3480, 3845, 4087, 4089, 5290, 6774, 7040
hsa05203	Viral carcinogenesis	13	20.63492	205	2.875175316	3.55E-07	595, 1017, 1019, 1021, 1026, 1027, 1029, 2885, 3845, 5290, 5925, 5966, 6774
hsa05212	Pancreatic cancer	12	19.04762	66	0.925666199	2.63E-11	595, 1019, 1021, 1029, 2064, 3845, 4087, 4089, 5290, 5925, 6774, 7040
hsa05215	Prostate cancer	12	19.04762	89	1.248246844	6.52E-10	367, 595, 1017, 1026, 1027, 2064, 2885, 3320, 3480, 3845, 5290, 5925
hsa01522	Endocrine resistance	12	19.04762	98	1.374474053	1.36E-09	595, 1019, 1026, 1027, 1029, 2064, 2099, 2885, 3480, 3845, 5290, 5925
hsa05224	Breast cancer	12	19.04762	146	2.047685835	8.88E-08	595, 672, 1019, 1021, 1026, 2064, 2099, 2885, 3480, 3845, 5290, 5925
hsa05166	HTLV-I infection	11	17.46032	258	3.618513324	0.000182	595, 1019, 1026, 1029, 3845, 4087, 4089, 5111, 5290, 5925, 7040
hsa05206	MicroRNAs in cancer	11	17.46032	299	4.193548387	0.000526	595, 672, 1021, 1026, 1027, 1029, 2064, 2885, 3845, 5290, 6774
hsa04710	Circadian rhythm	10	15.87302	31	0.434782609	8.11E-12	406, 1407, 1408, [illegible], 1454, 4862, 5187, 8863, 8864, [illegible]
hsa05214	Glioma	10	15.87302	66	0.925666199	6.08E-09	595, 1019, 1021, 1026, 1029, 2885, 3480, 3845, 5290, 5925
hsa04933	AGE-RAGE signaling pathway in diabetic complications	10	15.87302	101	1.41654979	2.91E-07	595, 1019, 1027, 1281, 3845, 4087, 4089, 5290, 6774, 7040
hsa05223	Non-small cell lung cancer	9	14.28571	56	0.785413745	2.78E-08	595, 1019, 1021, 1029, 2064, 2885, 3845, 5290, 5925
hsa05218	Melanoma	9	14.28571	71	0.995792426	1.93E-07	595, 1019, 1021, 1026, 1029, 3480, 3845, 5290, 5925
hsa05162	Measles	9	14.28571	136	1.90743338	0.000041	595, 868, 1017, 1019, 1021, 1027, [illegible], 5290, 6774
hsa04550	Signaling pathways regulating pluripotency of stem cells	8	12.69841	142	1.991584853	0.000355	2264, 2885, 3480, 3845, 4087, 4089, 5290, 6774
hsa05219	Bladder cancer	7	11.11111	41	0.575035063	9.59E-07	595, 1019, 1026, 1029, 2064, 3845, 5925
hsa05222	Small cell lung cancer	7	11.11111	86	1.206171108	0.000143	595, 1017, 1019, 1021, 1027, 5290, 5925

hsa04012	ErbB signaling pathway	7	11.11111	88	1.234221599	0.000158	868, 1026, 1027, 2064, 2885, 3845, 5290
hsa04066	HIF-1 signaling pathway	7	11.11111	103	1.444600281	0.000355	1026, 1027, 2064, 3091, 3480, 5290, 6774
hsa04390	Hippo signaling pathway	7	11.11111	154	2.159887798	0.003467	595, 1453, 1454, 4087, 4089, 5519, 7040
hsa05210	Colorectal cancer	6	9.52381	62	0.869565217	0.000208	595, 3845, 4087, 4089, 5290, 7040
hsa05211	Renal cell carcinoma	6	9.52381	67	0.939691445	0.000313	2034, 2885, 3091, 3845, 5290, 7040
hsa04115	p53 signaling pathway	6	9.52381	69	0.967741935	0.000355	595, 1017, 1019, 1021, 1026, 1029
hsa04917	Prolactin signaling pathway	6	9.52381	72	1.009817672	0.000406	595, 2099, 2885, 3845, 5290, 6774
hsa04520	Adherens junction	6	9.52381	74	1.037868163	0.000459	1457, 2064, 3480, 4087, 4089, 5787
hsa01521	EGFR tyrosine kinase inhibitor resistance	6	9.52381	81	1.136044881	0.000716	2064, 2885, 3480, 3845, 5290, 6774
hsa05213	Endometrial cancer	5	7.936508	52	0.729312763	0.000831	595, 2064, 2885, 3845, 5290
hsa05221	Acute myeloid leukemia	5	7.936508	57	0.79943899	0.001254	595, 2885, 3845, 5290, 6774
hsa01524	Platinum drug resistance	5	7.936508	75	1.051893408	0.004315	672, 1026, 1029, 2064, 5290

7B- Cd9KO

Identifier	Name	Foreground (in the list)	FG%	Background (whole genome)	BG%	p-value	Associated gene ids
hsa05200	Pathways in cancer	30	16.75978	397	5.56802244	1.6E-06	355, 387, 572, 841, 998, 1021, 1387, 1910, 2064, 2263, 2353, 2885, 3320, 3326, 3480, 3725, 3815, 3845, 4089, 4254, 4790, 5290, 5602, 5728, 5879, 5888, 5970, 7040, 7046, 7048
hsa04010	MAPK signaling pathway	28	15.64246	255	3.576437588	6.37E-09	355, 408, 409, 468, 998, 1647, 1649, 2263, 2264, 2316, 2353, 2885, 3312, 3315, 3725, 3727, 3845, 4217, 4790, 5602, 5879, 5906, 5970, 7040, 7046, 7048, 8569, 9064
hsa04151	PI3K-Akt signaling pathway	26	14.52514	341	4.782608696	7.3E-06	468, 572, 896, 1021, 1942, 1946, 1977, 2263, 2264, 2885, 3320, 3326, 3480, 3815, 3845, 4254, 4790, 5105, 5290, 5728, 5879, 5970, 7532, 7534, 11140, 64764

hsa05161	Hepatitis B	21	11.73184	146	2.047685835	6.37E-09	355, 468, 572, 841, 1021, 1387, 1654, 2353, 2885, 3725, 3845, 4089, 4790, 5290, 5602, 5728, 5970, 7040, 7046, 7534, 64764
hsa05203	Viral carcinogenesis	21	11.73184	205	2.875175316	1.5E-06	387, 468, 572, 841, 896, 998, 1021, 1387, 1654, 2885, 3725, 3845, 4790, 5290, 5879, 5966, 5970, 7532, 7534, 64764, 554313
hsa04014	Ras signaling pathway	19	10.61453	229	3.211781206	0.000078	387, 572, 998, 1942, 1946, 2263, 2264, 2885, 3480, 3815, 3845, 4254, 4790, 5290, 5602, 5879, 5906, 5966, 5970
hsa04210	Apoptosis	18	10.05587	140	1.963534362	6.08E-07	142, 355, 468, 572, 841, 1647, 1649, 2353, 3725, 3845, 4217, 4790, 5290, 5602, 5783, 5970, 7846, 8737
hsa04510	Focal adhesion	17	9.497207	203	2.847124825	0.000183	387, 572, 896, 998, 1729, 2064, 2316, 2534, 2885, 3480, 3725, 5290, 5602, 5728, 5879, 5906, 58498
hsa04144	Endocytosis	17	9.497207	260	3.646563815	0.002562	163, 387, 408, 409, 998, 2263, 2264, 3312, 3480, 3800, 3815, 7040, 7046, 7048, 7251, 55048, 116984
hsa04380	Osteoclast differentiation	16	8.938547	132	1.851332398	4.9E-06	2353, 2355, 2534, 2885, 3725, 3726, 3727, 3932, 4790, 5290, 5602, 5879, 5970, 7040, 7046, 7048
hsa04360	Axon guidance	16	8.938547	176	2.468443198	0.000131	387, 998, 1942, 1946, 1948, 1949, 2043, 2534, 3845, 5290, 5364, 5879, 8482, 10154, 27289, 29984
hsa05169	Epstein-Barr virus infection	16	8.938547	204	2.86115007	0.000599	1387, 1457, 1459, 3312, 3315, 3725, 4790, 5290, 5602, 5710, 5970, 7431, 7532, 7534, 8737, 51082
hsa05212	Pancreatic cancer	15	8.379888	66	0.925666199	6.37E-09	572, 998, 1021, 2064, 3845, 4089, 4790, 5290, 5602, 5879, 5888, 5970, 7040, 7046, 7048

hsa05215	Prostate cancer	15	8.379888	89	1.248246844	3.20E-07	468, 572, 1387, 2064, 2263, 2885, 3320, 3326, 3480, 3845, 4790, 5290, 5728, 5970, 64764
hsa04668	TNF signaling pathway	15	8.379888	110	1.542776999	2.8E-06	355, 468, 841, 1051, 2353, 3725, 3726, 4217, 4790, 5290, 5602, 5970, 8737, 9530, 64764
hsa04932	Non-alcoholic fatty liver disease (NAFLD)	15	8.379888	151	2.117812062	9.13E-05	355, 468, 841, 998, 1571, 1649, 3725, 4217, 4790, 5290, 5602, 5879, 5970, 7040, 9370
hsa04024	cAMP signaling pathway	15	8.379888	200	2.805049088	0.001483	387, 572, 1387, 2353, 2740, 2892, 3725, 3991, 4790, 5290, 5602, 5879, 5906, 5970, 64764
hsa04933	AGE-RAGE signaling pathway in diabetic complications	14	7.821229	101	1.41654979	5.1E-06	998, 1729, 3725, 3845, 4089, 4790, 5054, 5290, 5602, 5879, 5970, 7040, 7046, 7048
hsa04722	Neurotrophin signaling pathway	14	7.821229	121	1.697054698	3.53E-05	387, 468, 572, 998, 2885, 3725, 3845, 4217, 4790, 5290, 5602, 5879, 5906, 5970
hsa04068	FoxO signaling pathway	13	7.26257	134	1.879382889	0.000397	1387, 1647, 2885, 3480, 3845, 4089, 5105, 5290, 5602, 5728, 7040, 7046, 7048
hsa03040	Spliceosome	13	7.26257	134	1.879382889	0.000397	1665, 3183, 3312, 6625, 9879, 10262, 10291, 10992, 11338, 23350, 26121, 27339, 55660
hsa05152	Tuberculosis	13	7.26257	179	2.510518934	0.004217	387, 533, 572, 841, 1051, 1054, 1387, 4046, 4790, 5602, 5970, 7040, 64170
hsa05210	Colorectal cancer	12	6.703911	62	0.869565217	1.5E-06	387, 572, 2353, 3725, 3845, 4089, 5290, 5602, 5879, 7040, 7046, 7048
hsa04520	Adherens junction	12	6.703911	74	1.037868163	6.3E-06	387, 998, 1387, 1457, 1459, 2064, 2534, 3480, 4089, 5879, 7046, 7048
hsa05142	Chagas disease (American trypanosomiasis)	12	6.703911	104	1.458625526	0.000162	355, 841, 2353, 3725, 4790, 5054, 5290, 5602, 5970, 7040, 7046, 7048
hsa05220	Chronic myeloid leukemia	11	6.145251	73	1.023842917	3.53E-05	572, 1021, 2885, 3845, 4089, 4790, 5290, 5970, 7040, 7046, 7048
hsa04915	Estrogen signaling pathway	11	6.145251	100	1.402524544	0.000498	468, 2289, 2353, 2885, 3312, 3320, 3326, 3725, 3845, 5290, 64764

hsa04660	T cell receptor signaling pathway	11	6.145251	105	1.472650771	0.000695	387, 998, 2353, 2534, 2885, 3725, 3845, 3932, 4790, 5290, 5970
hsa04211	Longevity regulating pathway	10	5.586592	94	1.318373072	0.001236	468, 1977, 3480, 3845, 4790, 5290, 5970, 9370, 9821, 64764
hsa04066	HIF-1 signaling pathway	10	5.586592	103	1.444600281	0.002398	1387, 1977, 2064, 3480, 3939, 4790, 5054, 5290, 5970, 8569
hsa05211	Renal cell carcinoma	9	5.027933	67	0.939691445	0.000506	998, 1387, 2885, 3725, 3845, 5290, 5879, 5906, 7040
hsa01521	EGFR tyrosine kinase inhibitor resistance	9	5.027933	81	1.136044881	0.001826	572, 1977, 2064, 2263, 2885, 3480, 3845, 5290, 5728
hsa05132	Salmonella infection	9	5.027933	86	1.206171108	0.002627	998, 2316, 2353, 3725, 4790, 5602, 5879, 5970, 147700
hsa05416	Viral myocarditis	8	4.469274	59	0.827489481	0.00114	841, 1605, 1981, 2534, 4624, 5879, 6444, 6445
hsa04115	p53 signaling pathway	8	4.469274	69	0.967741935	0.002756	355, 841, 896, 1021, 1647, 2810, 5054, 5728
hsa04917	Prolactin signaling pathway	8	4.469274	72	1.009817672	0.003595	2001, 2353, 2885, 3845, 4790, 5290, 5602, 5970
hsa04662	B cell receptor signaling pathway	8	4.469274	73	1.023842917	0.003842	2353, 2885, 3725, 3845, 4790, 5290, 5879, 5970
hsa04621	NOD-like receptor signaling pathway	7	3.910615	57	0.79943899	0.004137	841, 3320, 3326, 4790, 5602, 5970, 64170
hsa05221	Acute myeloid leukemia	7	3.910615	57	0.79943899	0.004137	572, 2885, 3815, 3845, 4790, 5290, 5970

Supplementary Table S8B: Cd151KO and Cd9KO human orthologous sDEGs Lung Tissue filtered HCDP networks- List of Gene-Enriched KEGG pathway associations (*p* ≤0.005)

Cd151KO

Gene ID	Gene Symbol	Pathway ID	Pathway Name	KEGG Pathway sub-class
1017	CDK2	hsa04068	FoxO signaling pathway	Signal transduction
1017	CDK2	hsa04110	Cell cycle	Cell growth and death
1017	CDK2	hsa04115	p53 signaling pathway	Cell growth and death
1017	CDK2	hsa04151	PI3K-Akt signaling pathway	Signal transduction
1017	CDK2	hsa05161	Hepatitis B	Infectious diseases: Viral
1017	CDK2	hsa05162	Measles	Infectious diseases: Viral
1017	CDK2	hsa05200	Pathways in cancer	Cancers: Overview
1017	CDK2	hsa05203	Viral carcinogenesis	Cancers: Overview
1017	CDK2	hsa05215	Prostate cancer	Cancers: Specific types
1017	CDK2	hsa05222	Small cell lung cancer	Cancers: Specific types
1019	CDK4	hsa01522	Endocrine resistance	Drug resistance: Antineoplastic
1019	CDK4	hsa04110	Cell cycle	Cell growth and death
1019	CDK4	hsa04115	p53 signaling pathway	Cell growth and death
1019	CDK4	hsa04151	PI3K-Akt signaling pathway	Signal transduction
1019	CDK4	hsa04933	AGE-RAGE signaling pathway in diabetic complications	Endocrine and metabolic diseases
1019	CDK4	hsa05161	Hepatitis B	Infectious diseases: Viral
1019	CDK4	hsa05162	Measles	Infectious diseases: Viral
1019	CDK4	hsa05166	HTLV-I infection	Infectious diseases: Viral
1019	CDK4	hsa05200	Pathways in cancer	Cancers: Overview
1019	CDK4	hsa05203	Viral carcinogenesis	Cancers: Overview
1019	CDK4	hsa05212	Pancreatic cancer	Cancers: Specific types
1019	CDK4	hsa05214	Glioma	Cancers: Specific types
1019	CDK4	hsa05218	Melanoma	Cancers: Specific types
1019	CDK4	hsa05219	Bladder cancer	Cancers: Specific types
1019	CDK4	hsa05220	Chronic myeloid leukemia	Cancers: Specific types
1019	CDK4	hsa05222	Small cell lung cancer	Cancers: Specific types
1019	CDK4	hsa05223	Non-small cell lung cancer	Cancers: Specific types

1019	CDK4	hsa05224	Breast cancer	Cancers: Specific types
1021	CDK6	hsa04110	Cell cycle	Cell growth and death
1021	CDK6	hsa04115	p53 signaling pathway	Cell growth and death
1021	CDK6	hsa04151	PI3K-Akt signaling pathway	Signal transduction
1021	CDK6	hsa05161	Hepatitis B	Infectious diseases: Viral
1021	CDK6	hsa05162	Measles	Infectious diseases: Viral
1021	CDK6	hsa05200	Pathways in cancer	Cancers: Overview
1021	CDK6	hsa05203	Viral carcinogenesis	Cancers: Overview
1021	CDK6	hsa05206	MicroRNAs in cancer	Cancers: Overview
1021	CDK6	hsa05212	Pancreatic cancer	Cancers: Specific types
1021	CDK6	hsa05214	Glioma	Cancers: Specific types
1021	CDK6	hsa05218	Melanoma	Cancers: Specific types
1021	CDK6	hsa05220	Chronic myeloid leukemia	Cancers: Specific types
1021	CDK6	hsa05222	Small cell lung cancer	Cancers: Specific types
1021	CDK6	hsa05223	Non-small cell lung cancer	Cancers: Specific types
1021	CDK6	hsa05224	Breast cancer	Cancers: Specific types
1026	CDKN1A	hsa01522	Endocrine resistance	Drug resistance: Antineoplastic
1026	CDKN1A	hsa01524	Platinum drug resistance	Drug resistance: Antineoplastic
1026	CDKN1A	hsa04012	ErbB signaling pathway	Signal transduction
1026	CDKN1A	hsa04066	HIF-1 signaling pathway	Signal transduction
1026	CDKN1A	hsa04068	FoxO signaling pathway	Signal transduction
1026	CDKN1A	hsa04110	Cell cycle	Cell growth and death
1026	CDKN1A	hsa04115	p53 signaling pathway	Cell growth and death
1026	CDKN1A	hsa04151	PI3K-Akt signaling pathway	Signal transduction
1026	CDKN1A	hsa05161	Hepatitis B	Infectious diseases: Viral
1026	CDKN1A	hsa05166	HTLV-I infection	Infectious diseases: Viral
1026	CDKN1A	hsa05200	Pathways in cancer	Cancers: Overview
1026	CDKN1A	hsa05203	Viral carcinogenesis	Cancers: Overview
1026	CDKN1A	hsa05205	Proteoglycans in cancer	Cancers: Overview
1026	CDKN1A	hsa05206	MicroRNAs in cancer	Cancers: Overview
1026	CDKN1A	hsa05214	Glioma	Cancers: Specific types
1026	CDKN1A	hsa05215	Prostate cancer	Cancers: Specific types
1026	CDKN1A	hsa05218	Melanoma	Cancers: Specific types

1026	CDKN1A	hsa05219	Bladder cancer	Cancers: Specific types
1026	CDKN1A	hsa05220	Chronic myeloid leukemia	Cancers: Specific types
1026	CDKN1A	hsa05224	Breast cancer	Cancers: Specific types
1027	CDKN1B	hsa01522	Endocrine resistance	Drug resistance: Antineoplastic
1027	CDKN1B	hsa04012	ErbB signaling pathway	Signal transduction
1027	CDKN1B	hsa04066	HIF-1 signaling pathway	Signal transduction
1027	CDKN1B	hsa04068	FoxO signaling pathway	Signal transduction
1027	CDKN1B	hsa04110	Cell cycle	Cell growth and death
1027	CDKN1B	hsa04151	PI3K-Akt signaling pathway	Signal transduction
1027	CDKN1B	hsa04933	AGE-RAGE signaling pathway in diabetic complications	Endocrine and metabolic diseases
1027	CDKN1B	hsa05161	Hepatitis B	Infectious diseases: Viral
1027	CDKN1B	hsa05162	Measles	Infectious diseases: Viral
1027	CDKN1B	hsa05200	Pathways in cancer	Cancers: Overview
1027	CDKN1B	hsa05203	Viral carcinogenesis	Cancers: Overview
1027	CDKN1B	hsa05206	MicroRNAs in cancer	Cancers: Overview
1027	CDKN1B	hsa05215	Prostate cancer	Cancers: Specific types
1027	CDKN1B	hsa05220	Chronic myeloid leukemia	Cancers: Specific types
1027	CDKN1B	hsa05222	Small cell lung cancer	Cancers: Specific types
1029	CDKN2A	hsa01522	Endocrine resistance	Drug resistance: Antineoplastic
1029	CDKN2A	hsa01524	Platinum drug resistance	Drug resistance: Antineoplastic
1029	CDKN2A	hsa04110	Cell cycle	Cell growth and death
1029	CDKN2A	hsa04115	p53 signaling pathway	Cell growth and death
1029	CDKN2A	hsa05166	HTLV-I infection	Infectious diseases: Viral
1029	CDKN2A	hsa05200	Pathways in cancer	Cancers: Overview
1029	CDKN2A	hsa05203	Viral carcinogenesis	Cancers: Overview
1029	CDKN2A	hsa05206	MicroRNAs in cancer	Cancers: Overview
1029	CDKN2A	hsa05212	Pancreatic cancer	Cancers: Specific types
1029	CDKN2A	hsa05214	Glioma	Cancers: Specific types
1029	CDKN2A	hsa05218	Melanoma	Cancers: Specific types
1029	CDKN2A	hsa05219	Bladder cancer	Cancers: Specific types
1029	CDKN2A	hsa05220	Chronic myeloid leukemia	Cancers: Specific types
1029	CDKN2A	hsa05223	Non-small cell lung cancer	Cancers: Specific types

1281	COL3A1	hsa04933	AGE-RAGE signaling pathway in diabetic complications	Endocrine and metabolic diseases
1407	CRY1	hsa04710	Circadian rhythm	Environmental adaptation
1408	CRY2	hsa04710	Circadian rhythm	Environmental adaptation
1453	CSNK1D	hsa04390	Hippo signaling pathway	Signal transduction
1453	CSNK1D	hsa04710	Circadian rhythm	Environmental adaptation
1454	CSNK1E	hsa04068	FoxO signaling pathway	Signal transduction
1454	CSNK1E	hsa04390	Hippo signaling pathway	Signal transduction
1454	CSNK1E	hsa04710	Circadian rhythm	Environmental adaptation
1457	CSNK2A1	hsa04520	Adherens junction	Cellular community
1457	CSNK2A1	hsa05162	Measles	Infectious diseases: Viral
2034	EPAS1	hsa05200	Pathways in cancer	Cancers: Overview
2034	EPAS1	hsa05211	Renal cell carcinoma	Cancers: Specific types
2064	ERBB2	hsa01521	EGFR tyrosine kinase inhibitor resistance	Drug resistance: Antineoplastic
2064	ERBB2	hsa01522	Endocrine resistance	Drug resistance: Antineoplastic
2064	ERBB2	hsa01524	Platinum drug resistance	Drug resistance: Antineoplastic
2064	ERBB2	hsa04012	ErbB signaling pathway	Signal transduction
2064	ERBB2	hsa04066	HIF-1 signaling pathway	Signal transduction
2064	ERBB2	hsa04520	Adherens junction	Cellular community
2064	ERBB2	hsa05200	Pathways in cancer	Cancers: Overview
2064	ERBB2	hsa05205	Proteoglycans in cancer	Cancers: Overview
2064	ERBB2	hsa05206	MicroRNAs in cancer	Cancers: Overview
2064	ERBB2	hsa05212	Pancreatic cancer	Cancers: Specific types
2064	ERBB2	hsa05213	Endometrial cancer	Cancers: Specific types
2064	ERBB2	hsa05215	Prostate cancer	Cancers: Specific types
2064	ERBB2	hsa05219	Bladder cancer	Cancers: Specific types
2064	ERBB2	hsa05223	Non-small cell lung cancer	Cancers: Specific types
2064	ERBB2	hsa05224	Breast cancer	Cancers: Specific types
2099	ESR1	hsa01522	Endocrine resistance	Drug resistance: Antineoplastic
2099	ESR1	hsa04917	Prolactin signaling pathway	Endocrine system
2099	ESR1	hsa05205	Proteoglycans in cancer	Cancers: Overview
2099	ESR1	hsa05224	Breast cancer	Cancers: Specific types
2264	FGFR4	hsa04151	PI3K-Akt signaling pathway	Signal transduction

2264	FGFR4	hsa04550	Signaling pathways regulating pluripotency of stem cells	Cellular community
288	ANK3	hsa05205	Proteoglycans in cancer	Cancers: Overview
2885	GRB2	hsa01521	EGFR tyrosine kinase inhibitor resistance	Drug resistance: Antineoplastic
2885	GRB2	hsa01522	Endocrine resistance	Drug resistance: Antineoplastic
2885	GRB2	hsa04012	ErbB signaling pathway	Signal transduction
2885	GRB2	hsa04068	FoxO signaling pathway	Signal transduction
2885	GRB2	hsa04151	PI3K-Akt signaling pathway	Signal transduction
2885	GRB2	hsa04550	Signaling pathways regulating pluripotency of stem cells	Cellular community
2885	GRB2	hsa04917	Prolactin signaling pathway	Endocrine system
2885	GRB2	hsa05161	Hepatitis B	Infectious diseases: Viral
2885	GRB2	hsa05200	Pathways in cancer	Cancers: Overview
2885	GRB2	hsa05203	Viral carcinogenesis	Cancers: Overview
2885	GRB2	hsa05205	Proteoglycans in cancer	Cancers: Overview
2885	GRB2	hsa05206	MicroRNAs in cancer	Cancers: Overview
2885	GRB2	hsa05211	Renal cell carcinoma	Cancers: Specific types
2885	GRB2	hsa05213	Endometrial cancer	Cancers: Specific types
2885	GRB2	hsa05214	Glioma	Cancers: Specific types
2885	GRB2	hsa05215	Prostate cancer	Cancers: Specific types
2885	GRB2	hsa05220	Chronic myeloid leukemia	Cancers: Specific types
2885	GRB2	hsa05221	Acute myeloid leukemia	Cancers: Specific types
2885	GRB2	hsa05223	Non-small cell lung cancer	Cancers: Specific types
2885	GRB2	hsa05224	Breast cancer	Cancers: Specific types
3091	HIF1A	hsa04066	HIF-1 signaling pathway	Signal transduction
3091	HIF1A	hsa05200	Pathways in cancer	Cancers: Overview
3091	HIF1A	hsa05205	Proteoglycans in cancer	Cancers: Overview
3091	HIF1A	hsa05211	Renal cell carcinoma	Cancers: Specific types
3320	HSP90AA1	hsa04151	PI3K-Akt signaling pathway	Signal transduction
3320	HSP90AA1	hsa05200	Pathways in cancer	Cancers: Overview
3320	HSP90AA1	hsa05215	Prostate cancer	Cancers: Specific types
3480	IGF1R	hsa01521	EGFR tyrosine kinase inhibitor resistance	Drug resistance: Antineoplastic
3480	IGF1R	hsa01522	Endocrine resistance	Drug resistance: Antineoplastic

3480	IGF1R	hsa04066	HIF-1 signaling pathway	Signal transduction
3480	IGF1R	hsa04068	FoxO signaling pathway	Signal transduction
3480	IGF1R	hsa04151	PI3K-Akt signaling pathway	Signal transduction
3480	IGF1R	hsa04520	Adherens junction	Cellular community
3480	IGF1R	hsa04550	Signaling pathways regulating pluripotency of stem cells	Cellular community
3480	IGF1R	hsa05200	Pathways in cancer	Cancers: Overview
3480	IGF1R	hsa05205	Proteoglycans in cancer	Cancers: Overview
3480	IGF1R	hsa05214	Glioma	Cancers: Specific types
3480	IGF1R	hsa05215	Prostate cancer	Cancers: Specific types
3480	IGF1R	hsa05218	Melanoma	Cancers: Specific types
3480	IGF1R	hsa05224	Breast cancer	Cancers: Specific types
367	AR	hsa05200	Pathways in cancer	Cancers: Overview
367	AR	hsa05215	Prostate cancer	Cancers: Specific types
3845	KRAS	hsa01521	EGFR tyrosine kinase inhibitor resistance	Drug resistance: Antineoplastic
3845	KRAS	hsa01522	Endocrine resistance	Drug resistance: Antineoplastic
3845	KRAS	hsa04012	ErbB signaling pathway	Signal transduction
3845	KRAS	hsa04068	FoxO signaling pathway	Signal transduction
3845	KRAS	hsa04151	PI3K-Akt signaling pathway	Signal transduction
3845	KRAS	hsa04550	Signaling pathways regulating pluripotency of stem cells	Cellular community
3845	KRAS	hsa04917	Prolactin signaling pathway	Endocrine system
3845	KRAS	hsa04933	AGE-RAGE signaling pathway in diabetic complications	Endocrine and metabolic diseases
3845	KRAS	hsa05161	Hepatitis B	Infectious diseases: Viral
3845	KRAS	hsa05166	HTLV-I infection	Infectious diseases: Viral
3845	KRAS	hsa05200	Pathways in cancer	Cancers: Overview
3845	KRAS	hsa05203	Viral carcinogenesis	Cancers: Overview
3845	KRAS	hsa05205	Proteoglycans in cancer	Cancers: Overview
3845	KRAS	hsa05206	MicroRNAs in cancer	Cancers: Overview
3845	KRAS	hsa05210	Colorectal cancer	Cancers: Specific types
3845	KRAS	hsa05211	Renal cell carcinoma	Cancers: Specific types
3845	KRAS	hsa05212	Pancreatic cancer	Cancers: Specific types

3845	KRAS	hsa05213	Endometrial cancer	Cancers: Specific types
3845	KRAS	hsa05214	Glioma	Cancers: Specific types
3845	KRAS	hsa05215	Prostate cancer	Cancers: Specific types
3845	KRAS	hsa05218	Melanoma	Cancers: Specific types
3845	KRAS	hsa05219	Bladder cancer	Cancers: Specific types
3845	KRAS	hsa05220	Chronic myeloid leukemia	Cancers: Specific types
3845	KRAS	hsa05221	Acute myeloid leukemia	Cancers: Specific types
3845	KRAS	hsa05223	Non-small cell lung cancer	Cancers: Specific types
3845	KRAS	hsa05224	Breast cancer	Cancers: Specific types
406	ARNTL	hsa04710	Circadian rhythm	Environmental adaptation
4087	SMAD2	hsa04068	FoxO signaling pathway	Signal transduction
4087	SMAD2	hsa04110	Cell cycle	Cell growth and death
4087	SMAD2	hsa04390	Hippo signaling pathway	Signal transduction
4087	SMAD2	hsa04520	Adherens junction	Cellular community
4087	SMAD2	hsa04550	Signaling pathways regulating pluripotency of stem cells	Cellular community
4087	SMAD2	hsa04933	AGE-RAGE signaling pathway in diabetic complications	Endocrine and metabolic diseases
4087	SMAD2	hsa05166	HTLV-I infection	Infectious diseases: Viral
4087	SMAD2	hsa05200	Pathways in cancer	Cancers: Overview
4087	SMAD2	hsa05205	Proteoglycans in cancer	Cancers: Overview
4087	SMAD2	hsa05210	Colorectal cancer	Cancers: Specific types
4087	SMAD2	hsa05212	Pancreatic cancer	Cancers: Specific types
4089	SMAD4	hsa04068	FoxO signaling pathway	Signal transduction
4089	SMAD4	hsa04110	Cell cycle	Cell growth and death
4089	SMAD4	hsa04390	Hippo signaling pathway	Signal transduction
4089	SMAD4	hsa04520	Adherens junction	Cellular community
4089	SMAD4	hsa04550	Signaling pathways regulating pluripotency of stem cells	Cellular community
4089	SMAD4	hsa04933	AGE-RAGE signaling pathway in diabetic complications	Endocrine and metabolic diseases
4089	SMAD4	hsa05161	Hepatitis B	Infectious diseases: Viral
4089	SMAD4	hsa05166	HTLV-I infection	Infectious diseases: Viral

4089	SMAD4	hsa05200	Pathways in cancer	Cancers: Overview
4089	SMAD4	hsa05210	Colorectal cancer	Cancers: Specific types
4089	SMAD4	hsa05212	Pancreatic cancer	Cancers: Specific types
4089	SMAD4	hsa05220	Chronic myeloid leukemia	Cancers: Specific types
4862	NPAS2	hsa04710	Circadian rhythm	Environmental adaptation
5000	ORC4	hsa04110	Cell cycle	Cell growth and death
5111	PCNA	hsa04110	Cell cycle	Cell growth and death
5111	PCNA	hsa05161	Hepatitis B	Infectious diseases: Viral
5111	PCNA	hsa05166	HTLV-I infection	Infectious diseases: Viral
5187	PER1	hsa04710	Circadian rhythm	Environmental adaptation
5290	PIK3CA	hsa01521	EGFR tyrosine kinase inhibitor resistance	Drug resistance: Antineoplastic
5290	PIK3CA	hsa01522	Endocrine resistance	Drug resistance: Antineoplastic
5290	PIK3CA	hsa01524	Platinum drug resistance	Drug resistance: Antineoplastic
5290	PIK3CA	hsa04012	ErbB signaling pathway	Signal transduction
5290	PIK3CA	hsa04066	HIF-1 signaling pathway	Signal transduction
5290	PIK3CA	hsa04068	FoxO signaling pathway	Signal transduction
5290	PIK3CA	hsa04151	PI3K-Akt signaling pathway	Signal transduction
5290	PIK3CA	hsa04550	Signaling pathways regulating pluripotency of stem cells	Cellular community
5290	PIK3CA	hsa04917	Prolactin signaling pathway	Endocrine system
5290	PIK3CA	hsa04933	AGE-RAGE signaling pathway in diabetic complications	Endocrine and metabolic diseases
5290	PIK3CA	hsa05161	Hepatitis B	Infectious diseases: Viral
5290	PIK3CA	hsa05162	Measles	Infectious diseases: Viral
5290	PIK3CA	hsa05166	HTLV-I infection	Infectious diseases: Viral
5290	PIK3CA	hsa05200	Pathways in cancer	Cancers: Overview
5290	PIK3CA	hsa05203	Viral carcinogenesis	Cancers: Overview
5290	PIK3CA	hsa05205	Proteoglycans in cancer	Cancers: Overview
5290	PIK3CA	hsa05206	MicroRNAs in cancer	Cancers: Overview
5290	PIK3CA	hsa05210	Colorectal cancer	Cancers: Specific types
5290	PIK3CA	hsa05211	Renal cell carcinoma	Cancers: Specific types
5290	PIK3CA	hsa05212	Pancreatic cancer	Cancers: Specific types
5290	PIK3CA	hsa05213	Endometrial cancer	Cancers: Specific types

5290	PIK3CA	hsa05214	Glioma	Cancers: Specific types
5290	PIK3CA	hsa05215	Prostate cancer	Cancers: Specific types
5290	PIK3CA	hsa05218	Melanoma	Cancers: Specific types
5290	PIK3CA	hsa05220	Chronic myeloid leukemia	Cancers: Specific types
5290	PIK3CA	hsa05221	Acute myeloid leukemia	Cancers: Specific types
5290	PIK3CA	hsa05222	Small cell lung cancer	Cancers: Specific types
5290	PIK3CA	hsa05223	Non-small cell lung cancer	Cancers: Specific types
5290	PIK3CA	hsa05224	Breast cancer	Cancers: Specific types
5519	PPP2R1B	hsa04151	PI3K-Akt signaling pathway	Signal transduction
5519	PPP2R1B	hsa04390	Hippo signaling pathway	Signal transduction
5787	PTPRB	hsa04520	Adherens junction	Cellular community
5925	RB1	hsa01522	Endocrine resistance	Drug resistance: Antineoplastic
5925	RB1	hsa04110	Cell cycle	Cell growth and death
5925	RB1	hsa05161	Hepatitis B	Infectious diseases: Viral
5925	RB1	hsa05166	HTLV-I infection	Infectious diseases: Viral
5925	RB1	hsa05200	Pathways in cancer	Cancers: Overview
5925	RB1	hsa05203	Viral carcinogenesis	Cancers: Overview
5925	RB1	hsa05212	Pancreatic cancer	Cancers: Specific types
5925	RB1	hsa05214	Glioma	Cancers: Specific types
5925	RB1	hsa05215	Prostate cancer	Cancers: Specific types
5925	RB1	hsa05218	Melanoma	Cancers: Specific types
5925	RB1	hsa05219	Bladder cancer	Cancers: Specific types
5925	RB1	hsa05220	Chronic myeloid leukemia	Cancers: Specific types
5925	RB1	hsa05222	Small cell lung cancer	Cancers: Specific types
5925	RB1	hsa05223	Non-small cell lung cancer	Cancers: Specific types
5925	RB1	hsa05224	Breast cancer	Cancers: Specific types
595	CCND1	hsa01522	Endocrine resistance	Drug resistance: Antineoplastic
595	CCND1	hsa04068	FoxO signaling pathway	Signal transduction
595	CCND1	hsa04110	Cell cycle	Cell growth and death
595	CCND1	hsa04115	p53 signaling pathway	Cell growth and death
595	CCND1	hsa04151	PI3K-Akt signaling pathway	Signal transduction
595	CCND1	hsa04390	Hippo signaling pathway	Signal transduction
595	CCND1	hsa04917	Prolactin signaling pathway	Endocrine system

595	CCND1	hsa04933	AGE-RAGE signaling pathway in diabetic complications	Endocrine and metabolic diseases
595	CCND1	hsa05161	Hepatitis B	Infectious diseases: Viral
595	CCND1	hsa05162	Measles	Infectious diseases: Viral
595	CCND1	hsa05166	HTLV-I infection	Infectious diseases: Viral
595	CCND1	hsa05200	Pathways in cancer	Cancers: Overview
595	CCND1	hsa05203	Viral carcinogenesis	Cancers: Overview
595	CCND1	hsa05205	Proteoglycans in cancer	Cancers: Overview
595	CCND1	hsa05206	MicroRNAs in cancer	Cancers: Overview
595	CCND1	hsa05210	Colorectal cancer	Cancers: Specific types
595	CCND1	hsa05212	Pancreatic cancer	Cancers: Specific types
595	CCND1	hsa05213	Endometrial cancer	Cancers: Specific types
595	CCND1	hsa05214	Glioma	Cancers: Specific types
595	CCND1	hsa05215	Prostate cancer	Cancers: Specific types
595	CCND1	hsa05218	Melanoma	Cancers: Specific types
595	CCND1	hsa05219	Bladder cancer	Cancers: Specific types
595	CCND1	hsa05220	Chronic myeloid leukemia	Cancers: Specific types
595	CCND1	hsa05221	Acute myeloid leukemia	Cancers: Specific types
595	CCND1	hsa05222	Small cell lung cancer	Cancers: Specific types
595	CCND1	hsa05223	Non-small cell lung cancer	Cancers: Specific types
595	CCND1	hsa05224	Breast cancer	Cancers: Specific types
5966	REL	hsa05203	Viral carcinogenesis	Cancers: Overview
672	BRCA1	hsa01524	Platinum drug resistance	Drug resistance: Antineoplastic
672	BRCA1	hsa04151	PI3K-Akt signaling pathway	Signal transduction
672	BRCA1	hsa05206	MicroRNAs in cancer	Cancers: Overview
672	BRCA1	hsa05224	Breast cancer	Cancers: Specific types
6774	STAT3	hsa01521	EGFR tyrosine kinase inhibitor resistance	Drug resistance: Antineoplastic
6774	STAT3	hsa04066	HIF-1 signaling pathway	Signal transduction
6774	STAT3	hsa04068	FoxO signaling pathway	Signal transduction
6774	STAT3	hsa04550	Signaling pathways regulating pluripotency of stem cells	Cellular community
6774	STAT3	hsa04917	Prolactin signaling pathway	Endocrine system

6774	STAT3	hsa04933	AGE-RAGE signaling pathway in diabetic complications	Endocrine and metabolic diseases
6774	STAT3	hsa05161	Hepatitis B	Infectious diseases: Viral
6774	STAT3	hsa05162	Measles	Infectious diseases: Viral
6774	STAT3	hsa05200	Pathways in cancer	Cancers: Overview
6774	STAT3	hsa05203	Viral carcinogenesis	Cancers: Overview
6774	STAT3	hsa05205	Proteoglycans in cancer	Cancers: Overview
6774	STAT3	hsa05206	MicroRNAs in cancer	Cancers: Overview
6774	STAT3	hsa05212	Pancreatic cancer	Cancers: Specific types
6774	STAT3	hsa05221	Acute myeloid leukemia	Cancers: Specific types
7040	TGFB1	hsa04068	FoxO signaling pathway	Signal transduction
7040	TGFB1	hsa04110	Cell cycle	Cell growth and death
7040	TGFB1	hsa04390	Hippo signaling pathway	Signal transduction
7040	TGFB1	hsa04933	AGE-RAGE signaling pathway in diabetic complications	Endocrine and metabolic diseases
7040	TGFB1	hsa05161	Hepatitis B	Infectious diseases: Viral
7040	TGFB1	hsa05166	HTLV-I infection	Infectious diseases: Viral
7040	TGFB1	hsa05200	Pathways in cancer	Cancers: Overview
7040	TGFB1	hsa05205	Proteoglycans in cancer	Cancers: Overview
7040	TGFB1	hsa05210	Colorectal cancer	Cancers: Specific types
7040	TGFB1	hsa05211	Renal cell carcinoma	Cancers: Specific types
7040	TGFB1	hsa05212	Pancreatic cancer	Cancers: Specific types
7040	TGFB1	hsa05220	Chronic myeloid leukemia	Cancers: Specific types
868	CBLB	hsa04012	ErbB signaling pathway	Signal transduction
868	CBLB	hsa05162	Measles	Infectious diseases: Viral
868	CBLB	hsa05200	Pathways in cancer	Cancers: Overview
868	CBLB	hsa05205	Proteoglycans in cancer	Cancers: Overview
868	CBLB	hsa05220	Chronic myeloid leukemia	Cancers: Specific types
8863	PER3	hsa04710	Circadian rhythm	Environmental adaptation
8864	PER2	hsa04710	Circadian rhythm	Environmental adaptation
9575	CLOCK	hsa04710	Circadian rhythm	Environmental adaptation

Cd9KO

Gene ID	Gene Symbol	Pathway ID	Pathway Name	KEGG Pathway sub-class
10154	PLXNC1	hsa04360	Axon guidance	Development
1021	CDK6	hsa04115	p53 signaling pathway	Cell growth and death
1021	CDK6	hsa04151	PI3K-Akt signaling pathway	Signal transduction
1021	CDK6	hsa05161	Hepatitis B	Infectious diseases: Viral
1021	CDK6	hsa05200	Pathways in cancer	Cancers: Overview
1021	CDK6	hsa05203	Viral carcinogenesis	Cancers: Overview
1021	CDK6	hsa05212	Pancreatic cancer	Cancers: Specific types
1021	CDK6	hsa05220	Chronic myeloid leukemia	Cancers: Specific types
10262	SF3B4	hsa03040	Spliceosome	Transcription
10291	SF3A1	hsa03040	Spliceosome	Transcription
1051	CEBPB	hsa04668	TNF signaling pathway	Signal transduction
1051	CEBPB	hsa05152	Tuberculosis	Infectious diseases: Bacterial
1054	CEBPG	hsa05152	Tuberculosis	Infectious diseases: Bacterial
10992	SF3B2	hsa03040	Spliceosome	Transcription
11140	CDC37	hsa04151	PI3K-Akt signaling pathway	Signal transduction
11338	U2AF2	hsa03040	Spliceosome	Transcription
116984	ARAP2	hsa04144	Endocytosis	Transport and catabolism
1387	CREBBP	hsa04024	cAMP signaling pathway	Signal transduction
1387	CREBBP	hsa04066	HIF-1 signaling pathway	Signal transduction
1387	CREBBP	hsa04068	FoxO signaling pathway	Signal transduction
1387	CREBBP	hsa04520	Adherens junction	Cellular community
1387	CREBBP	hsa05152	Tuberculosis	Infectious diseases: Bacterial
1387	CREBBP	hsa05161	Hepatitis B	Infectious diseases: Viral
1387	CREBBP	hsa05169	Epstein-Barr virus infection	Infectious diseases: Viral
1387	CREBBP	hsa05200	Pathways in cancer	Cancers: Overview
1387	CREBBP	hsa05203	Viral carcinogenesis	Cancers: Overview
1387	CREBBP	hsa05211	Renal cell carcinoma	Cancers: Specific types
1387	CREBBP	hsa05215	Prostate cancer	Cancers: Specific types
142	PARP1	hsa04210	Apoptosis	Cell growth and death
1457	CSNK2A1	hsa04520	Adherens junction	Cellular community
1457	CSNK2A1	hsa05169	Epstein-Barr virus infection	Infectious diseases: Viral
1459	CSNK2A2	hsa04520	Adherens junction	Cellular community

1459	CSNK2A2	hsa05169	Epstein-Barr virus infection	Infectious diseases: Viral
147700	KLC3	hsa05132	Salmonella infection	Infectious diseases: Bacterial
1571	CYP2E1	hsa04932	Non-alcoholic fatty liver disease (NAFLD)	Endocrine and metabolic diseases
1605	DAG1	hsa05416	Viral myocarditis	Cardiovascular diseases
163	AP2B1	hsa04144	Endocytosis	Transport and catabolism
1647	GADD45A	hsa04010	MAPK signaling pathway	Signal transduction
1647	GADD45A	hsa04068	FoxO signaling pathway	Signal transduction
1647	GADD45A	hsa04115	p53 signaling pathway	Cell growth and death
1647	GADD45A	hsa04210	Apoptosis	Cell growth and death
1649	DDIT3	hsa04010	MAPK signaling pathway	Signal transduction
1649	DDIT3	hsa04210	Apoptosis	Cell growth and death
1649	DDIT3	hsa04932	Non-alcoholic fatty liver disease (NAFLD)	Endocrine and metabolic diseases
1654	DDX3X	hsa05161	Hepatitis B	Infectious diseases: Viral
1654	DDX3X	hsa05203	Viral carcinogenesis	Cancers: Overview
1665	DHX15	hsa03040	Spliceosome	Transcription
1729	DIAPH1	hsa04510	Focal adhesion	Cellular community
1729	DIAPH1	hsa04933	AGE-RAGE signaling pathway in diabetic complications	Endocrine and metabolic diseases
1910	EDNRB	hsa05200	Pathways in cancer	Cancers: Overview
1942	EFNA1	hsa04014	Ras signaling pathway	Signal transduction
1942	EFNA1	hsa04151	PI3K-Akt signaling pathway	Signal transduction
1942	EFNA1	hsa04360	Axon guidance	Development
1946	EFNA5	hsa04014	Ras signaling pathway	Signal transduction
1946	EFNA5	hsa04151	PI3K-Akt signaling pathway	Signal transduction
1946	EFNA5	hsa04360	Axon guidance	Development
1948	EFNB2	hsa04360	Axon guidance	Development
1949	EFNB3	hsa04360	Axon guidance	Development
1977	EIF4E	hsa01521	EGFR tyrosine kinase inhibitor resistance	Drug resistance: Antineoplastic
1977	EIF4E	hsa04066	HIF-1 signaling pathway	Signal transduction
1977	EIF4E	hsa04151	PI3K-Akt signaling pathway	Signal transduction
1977	EIF4E	hsa04211	Longevity regulating pathway	Aging
1981	EIF4G1	hsa05416	Viral myocarditis	Cardiovascular diseases
2001	ELF5	hsa04917	Prolactin signaling pathway	Endocrine system

2043	EPHA4	hsa04360	Axon guidance	Development
2064	ERBB2	hsa01521	EGFR tyrosine kinase inhibitor resistance	Drug resistance: Antineoplastic
2064	ERBB2	hsa04066	HIF-1 signaling pathway	Signal transduction
2064	ERBB2	hsa04510	Focal adhesion	Cellular community
2064	ERBB2	hsa04520	Adherens junction	Cellular community
2064	ERBB2	hsa05200	Pathways in cancer	Cancers: Overview
2064	ERBB2	hsa05212	Pancreatic cancer	Cancers: Specific types
2064	ERBB2	hsa05215	Prostate cancer	Cancers: Specific types
2263	FGFR2	hsa01521	EGFR tyrosine kinase inhibitor resistance	Drug resistance: Antineoplastic
2263	FGFR2	hsa04010	MAPK signaling pathway	Signal transduction
2263	FGFR2	hsa04014	Ras signaling pathway	Signal transduction
2263	FGFR2	hsa04144	Endocytosis	Transport and catabolism
2263	FGFR2	hsa04151	PI3K-Akt signaling pathway	Signal transduction
2263	FGFR2	hsa05200	Pathways in cancer	Cancers: Overview
2263	FGFR2	hsa05215	Prostate cancer	Cancers: Specific types
2264	FGFR4	hsa04010	MAPK signaling pathway	Signal transduction
2264	FGFR4	hsa04014	Ras signaling pathway	Signal transduction
2264	FGFR4	hsa04144	Endocytosis	Transport and catabolism
2264	FGFR4	hsa04151	PI3K-Akt signaling pathway	Signal transduction
2289	FKBP5	hsa04915	Estrogen signaling pathway	Endocrine system
2316	FLNA	hsa04010	MAPK signaling pathway	Signal transduction
2316	FLNA	hsa04510	Focal adhesion	Cellular community
2316	FLNA	hsa05132	Salmonella infection	Infectious diseases: Bacterial
23350	U2SURP	hsa03040	Spliceosome	Transcription
2353	FOS	hsa04010	MAPK signaling pathway	Signal transduction
2353	FOS	hsa04024	cAMP signaling pathway	Signal transduction
2353	FOS	hsa04210	Apoptosis	Cell growth and death
2353	FOS	hsa04380	Osteoclast differentiation	Development
2353	FOS	hsa04660	T cell receptor signaling pathway	Immune system
2353	FOS	hsa04662	B cell receptor signaling pathway	Immune system
2353	FOS	hsa04668	TNF signaling pathway	Signal transduction
2353	FOS	hsa04915	Estrogen signaling pathway	Endocrine system
2353	FOS	hsa04917	Prolactin signaling pathway	Endocrine system

2353	FOS	hsa05132	Salmonella infection	Infectious diseases: Bacterial
2353	FOS	hsa05142	Chagas disease (American trypanosomiasis)	Infectious diseases: Parasitic
2353	FOS	hsa05161	Hepatitis B	Infectious diseases: Viral
2353	FOS	hsa05200	Pathways in cancer	Cancers: Overview
2353	FOS	hsa05210	Colorectal cancer	Cancers: Specific types
2355	FOSL2	hsa04380	Osteoclast differentiation	Development
2534	FYN	hsa04360	Axon guidance	Development
2534	FYN	hsa04380	Osteoclast differentiation	Development
2534	FYN	hsa04510	Focal adhesion	Cellular community
2534	FYN	hsa04520	Adherens junction	Cellular community
2534	FYN	hsa04660	T cell receptor signaling pathway	Immune system
2534	FYN	hsa05416	Viral myocarditis	Cardiovascular diseases
26121	PRPF31	hsa03040	Spliceosome	Transcription
27289	RND1	hsa04360	Axon guidance	Development
27339	PRPF19	hsa03040	Spliceosome	Transcription
2740	GLP1R	hsa04024	cAMP signaling pathway	Signal transduction
2810	SFN	hsa04115	p53 signaling pathway	Cell growth and death
2885	GRB2	hsa01521	EGFR tyrosine kinase inhibitor resistance	Drug resistance: Antineoplastic
2885	GRB2	hsa04010	MAPK signaling pathway	Signal transduction
2885	GRB2	hsa04014	Ras signaling pathway	Signal transduction
2885	GRB2	hsa04068	FoxO signaling pathway	Signal transduction
2885	GRB2	hsa04151	PI3K-Akt signaling pathway	Signal transduction
2885	GRB2	hsa04380	Osteoclast differentiation	Development
2885	GRB2	hsa04510	Focal adhesion	Cellular community
2885	GRB2	hsa04660	T cell receptor signaling pathway	Immune system
2885	GRB2	hsa04662	B cell receptor signaling pathway	Immune system
2885	GRB2	hsa04722	Neurotrophin signaling pathway	Nervous system
2885	GRB2	hsa04915	Estrogen signaling pathway	Endocrine system
2885	GRB2	hsa04917	Prolactin signaling pathway	Endocrine system
2885	GRB2	hsa05161	Hepatitis B	Infectious diseases: Viral
2885	GRB2	hsa05200	Pathways in cancer	Cancers: Overview
2885	GRB2	hsa05203	Viral carcinogenesis	Cancers: Overview

2885	GRB2	hsa05211	Renal cell carcinoma	Cancers: Specific types
2885	GRB2	hsa05215	Prostate cancer	Cancers: Specific types
2885	GRB2	hsa05220	Chronic myeloid leukemia	Cancers: Specific types
2885	GRB2	hsa05221	Acute myeloid leukemia	Cancers: Specific types
2892	GRIA3	hsa04024	cAMP signaling pathway	Signal transduction
29984	RHOD	hsa04360	Axon guidance	Development
3183	HNRNPC	hsa03040	Spliceosome	Transcription
3312	HSPA8	hsa03040	Spliceosome	Transcription
3312	HSPA8	hsa04010	MAPK signaling pathway	Signal transduction
3312	HSPA8	hsa04144	Endocytosis	Transport and catabolism
3312	HSPA8	hsa04915	Estrogen signaling pathway	Endocrine system
3312	HSPA8	hsa05169	Epstein-Barr virus infection	Infectious diseases: Viral
3315	HSPB1	hsa04010	MAPK signaling pathway	Signal transduction
3315	HSPB1	hsa05169	Epstein-Barr virus infection	Infectious diseases: Viral
3320	HSP90AA1	hsa04151	PI3K-Akt signaling pathway	Signal transduction
3320	HSP90AA1	hsa04621	NOD-like receptor signaling pathway	Immune system
3320	HSP90AA1	hsa04915	Estrogen signaling pathway	Endocrine system
3320	HSP90AA1	hsa05200	Pathways in cancer	Cancers: Overview
3320	HSP90AA1	hsa05215	Prostate cancer	Cancers: Specific types
3326	HSP90AB1	hsa04151	PI3K-Akt signaling pathway	Signal transduction
3326	HSP90AB1	hsa04621	NOD-like receptor signaling pathway	Immune system
3326	HSP90AB1	hsa04915	Estrogen signaling pathway	Endocrine system
3326	HSP90AB1	hsa05200	Pathways in cancer	Cancers: Overview
3326	HSP90AB1	hsa05215	Prostate cancer	Cancers: Specific types
3480	IGF1R	hsa01521	EGFR tyrosine kinase inhibitor resistance	Drug resistance: Antineoplastic
3480	IGF1R	hsa04014	Ras signaling pathway	Signal transduction
3480	IGF1R	hsa04066	HIF-1 signaling pathway	Signal transduction
3480	IGF1R	hsa04068	FoxO signaling pathway	Signal transduction
3480	IGF1R	hsa04144	Endocytosis	Transport and catabolism
3480	IGF1R	hsa04151	PI3K-Akt signaling pathway	Signal transduction
3480	IGF1R	hsa04211	Longevity regulating pathway	Aging
3480	IGF1R	hsa04510	Focal adhesion	Cellular community
3480	IGF1R	hsa04520	Adherens junction	Cellular community

3480	IGF1R	hsa05200	Pathways in cancer	Cancers: Overview
3480	IGF1R	hsa05215	Prostate cancer	Cancers: Specific types
355	FAS	hsa04010	MAPK signaling pathway	Signal transduction
355	FAS	hsa04115	p53 signaling pathway	Cell growth and death
355	FAS	hsa04210	Apoptosis	Cell growth and death
355	FAS	hsa04668	TNF signaling pathway	Signal transduction
355	FAS	hsa04932	Non-alcoholic fatty liver disease (NAFLD)	Endocrine and metabolic diseases
355	FAS	hsa05142	Chagas disease (American trypanosomiasis)	Infectious diseases: Parasitic
355	FAS	hsa05161	Hepatitis B	Infectious diseases: Viral
355	FAS	hsa05200	Pathways in cancer	Cancers: Overview
3725	JUN	hsa04010	MAPK signaling pathway	Signal transduction
3725	JUN	hsa04024	cAMP signaling pathway	Signal transduction
3725	JUN	hsa04210	Apoptosis	Cell growth and death
3725	JUN	hsa04380	Osteoclast differentiation	Development
3725	JUN	hsa04510	Focal adhesion	Cellular community
3725	JUN	hsa04660	T cell receptor signaling pathway	Immune system
3725	JUN	hsa04662	B cell receptor signaling pathway	Immune system
3725	JUN	hsa04668	TNF signaling pathway	Signal transduction
3725	JUN	hsa04722	Neurotrophin signaling pathway	Nervous system
3725	JUN	hsa04915	Estrogen signaling pathway	Endocrine system
3725	JUN	hsa04932	Non-alcoholic fatty liver disease (NAFLD)	Endocrine and metabolic diseases
3725	JUN	hsa04933	AGE-RAGE signaling pathway in diabetic complications	Endocrine and metabolic diseases
3725	JUN	hsa05132	Salmonella infection	Infectious diseases: Bacterial
3725	JUN	hsa05142	Chagas disease (American trypanosomiasis)	Infectious diseases: Parasitic
3725	JUN	hsa05161	Hepatitis B	Infectious diseases: Viral
3725	JUN	hsa05169	Epstein-Barr virus infection	Infectious diseases: Viral
3725	JUN	hsa05200	Pathways in cancer	Cancers: Overview
3725	JUN	hsa05203	Viral carcinogenesis	Cancers: Overview
3725	JUN	hsa05210	Colorectal cancer	Cancers: Specific types
3725	JUN	hsa05211	Renal cell carcinoma	Cancers: Specific types

3726	JUNB	hsa04380	Osteoclast differentiation	Development
3726	JUNB	hsa04668	TNF signaling pathway	Signal transduction
3727	JUND	hsa04010	MAPK signaling pathway	Signal transduction
3727	JUND	hsa04380	Osteoclast differentiation	Development
3800	KIF5C	hsa04144	Endocytosis	Transport and catabolism
3815	KIT	hsa04014	Ras signaling pathway	Signal transduction
3815	KIT	hsa04144	Endocytosis	Transport and catabolism
3815	KIT	hsa04151	PI3K-Akt signaling pathway	Signal transduction
3815	KIT	hsa05200	Pathways in cancer	Cancers: Overview
3815	KIT	hsa05221	Acute myeloid leukemia	Cancers: Specific types
3845	KRAS	hsa01521	EGFR tyrosine kinase inhibitor resistance	Drug resistance: Antineoplastic
3845	KRAS	hsa04010	MAPK signaling pathway	Signal transduction
3845	KRAS	hsa04014	Ras signaling pathway	Signal transduction
3845	KRAS	hsa04068	FoxO signaling pathway	Signal transduction
3845	KRAS	hsa04151	PI3K-Akt signaling pathway	Signal transduction
3845	KRAS	hsa04210	Apoptosis	Cell growth and death
3845	KRAS	hsa04211	Longevity regulating pathway	Aging
3845	KRAS	hsa04360	Axon guidance	Development
3845	KRAS	hsa04660	T cell receptor signaling pathway	Immune system
3845	KRAS	hsa04662	B cell receptor signaling pathway	Immune system
3845	KRAS	hsa04722	Neurotrophin signaling pathway	Nervous system
3845	KRAS	hsa04915	Estrogen signaling pathway	Endocrine system
3845	KRAS	hsa04917	Prolactin signaling pathway	Endocrine system
3845	KRAS	hsa04933	AGE-RAGE signaling pathway in diabetic complications	Endocrine and metabolic diseases
3845	KRAS	hsa05161	Hepatitis B	Infectious diseases: Viral
3845	KRAS	hsa05200	Pathways in cancer	Cancers: Overview
3845	KRAS	hsa05203	Viral carcinogenesis	Cancers: Overview
3845	KRAS	hsa05210	Colorectal cancer	Cancers: Specific types
3845	KRAS	hsa05211	Renal cell carcinoma	Cancers: Specific types
3845	KRAS	hsa05212	Pancreatic cancer	Cancers: Specific types
3845	KRAS	hsa05215	Prostate cancer	Cancers: Specific types
3845	KRAS	hsa05220	Chronic myeloid leukemia	Cancers: Specific types

3845	KRAS	hsa05221	Acute myeloid leukemia	Cancers: Specific types
387	RHOA	hsa04014	Ras signaling pathway	Signal transduction
387	RHOA	hsa04024	cAMP signaling pathway	Signal transduction
387	RHOA	hsa04144	Endocytosis	Transport and catabolism
387	RHOA	hsa04360	Axon guidance	Development
387	RHOA	hsa04510	Focal adhesion	Cellular community
387	RHOA	hsa04520	Adherens junction	Cellular community
387	RHOA	hsa04660	T cell receptor signaling pathway	Immune system
387	RHOA	hsa04722	Neurotrophin signaling pathway	Nervous system
387	RHOA	hsa05152	Tuberculosis	Infectious diseases: Bacterial
387	RHOA	hsa05200	Pathways in cancer	Cancers: Overview
387	RHOA	hsa05203	Viral carcinogenesis	Cancers: Overview
387	RHOA	hsa05210	Colorectal cancer	Cancers: Specific types
3932	LCK	hsa04380	Osteoclast differentiation	Development
3932	LCK	hsa04660	T cell receptor signaling pathway	Immune system
3939	LDHA	hsa04066	HIF-1 signaling pathway	Signal transduction
3991	LIPE	hsa04024	cAMP signaling pathway	Signal transduction
4046	LSP1	hsa05152	Tuberculosis	Infectious diseases: Bacterial
408	ARRB1	hsa04010	MAPK signaling pathway	Signal transduction
408	ARRB1	hsa04144	Endocytosis	Transport and catabolism
4089	SMAD4	hsa04068	FoxO signaling pathway	Signal transduction
4089	SMAD4	hsa04520	Adherens junction	Cellular community
4089	SMAD4	hsa04933	AGE-RAGE signaling pathway in diabetic complications	Endocrine and metabolic diseases
4089	SMAD4	hsa05161	Hepatitis B	Infectious diseases: Viral
4089	SMAD4	hsa05200	Pathways in cancer	Cancers: Overview
4089	SMAD4	hsa05210	Colorectal cancer	Cancers: Specific types
4089	SMAD4	hsa05212	Pancreatic cancer	Cancers: Specific types
4089	SMAD4	hsa05220	Chronic myeloid leukemia	Cancers: Specific types
409	ARRB2	hsa04010	MAPK signaling pathway	Signal transduction
409	ARRB2	hsa04144	Endocytosis	Transport and catabolism
4217	MAP3K5	hsa04010	MAPK signaling pathway	Signal transduction
4217	MAP3K5	hsa04210	Apoptosis	Cell growth and death

4217	MAP3K5	hsa04668	TNF signaling pathway	Signal transduction
4217	MAP3K5	hsa04722	Neurotrophin signaling pathway	Nervous system
4217	MAP3K5	hsa04932	Non-alcoholic fatty liver disease (NAFLD)	Endocrine and metabolic diseases
4254	KITLG	hsa04014	Ras signaling pathway	Signal transduction
4254	KITLG	hsa04151	PI3K-Akt signaling pathway	Signal transduction
4254	KITLG	hsa05200	Pathways in cancer	Cancers: Overview
4624	MYH6	hsa05416	Viral myocarditis	Cardiovascular diseases
468	ATF4	hsa04010	MAPK signaling pathway	Signal transduction
468	ATF4	hsa04151	PI3K-Akt signaling pathway	Signal transduction
468	ATF4	hsa04210	Apoptosis	Cell growth and death
468	ATF4	hsa04211	Longevity regulating pathway	Aging
468	ATF4	hsa04668	TNF signaling pathway	Signal transduction
468	ATF4	hsa04722	Neurotrophin signaling pathway	Nervous system
468	ATF4	hsa04915	Estrogen signaling pathway	Endocrine system
468	ATF4	hsa04932	Non-alcoholic fatty liver disease (NAFLD)	Endocrine and metabolic diseases
468	ATF4	hsa05161	Hepatitis B	Infectious diseases: Viral
468	ATF4	hsa05203	Viral carcinogenesis	Cancers: Overview
468	ATF4	hsa05215	Prostate cancer	Cancers: Specific types
4790	NFKB1	hsa04010	MAPK signaling pathway	Signal transduction
4790	NFKB1	hsa04014	Ras signaling pathway	Signal transduction
4790	NFKB1	hsa04024	cAMP signaling pathway	Signal transduction
4790	NFKB1	hsa04066	HIF-1 signaling pathway	Signal transduction
4790	NFKB1	hsa04151	PI3K-Akt signaling pathway	Signal transduction
4790	NFKB1	hsa04210	Apoptosis	Cell growth and death
4790	NFKB1	hsa04211	Longevity regulating pathway	Aging
4790	NFKB1	hsa04380	Osteoclast differentiation	Development
4790	NFKB1	hsa04621	NOD-like receptor signaling pathway	Immune system
4790	NFKB1	hsa04660	T cell receptor signaling pathway	Immune system
4790	NFKB1	hsa04662	B cell receptor signaling pathway	Immune system
4790	NFKB1	hsa04668	TNF signaling pathway	Signal transduction
4790	NFKB1	hsa04722	Neurotrophin signaling pathway	Nervous system
4790	NFKB1	hsa04917	Prolactin signaling pathway	Endocrine system
4790	NFKB1	hsa04932	Non-alcoholic fatty liver disease (NAFLD)	Endocrine and metabolic diseases

4790	NFKB1	hsa04933	AGE-RAGE signaling pathway in diabetic complications	Endocrine and metabolic diseases
4790	NFKB1	hsa05132	Salmonella infection	Infectious diseases: Bacterial
4790	NFKB1	hsa05142	Chagas disease (American trypanosomiasis)	Infectious diseases: Parasitic
4790	NFKB1	hsa05152	Tuberculosis	Infectious diseases: Bacterial
4790	NFKB1	hsa05161	Hepatitis B	Infectious diseases: Viral
4790	NFKB1	hsa05169	Epstein-Barr virus infection	Infectious diseases: Viral
4790	NFKB1	hsa05200	Pathways in cancer	Cancers: Overview
4790	NFKB1	hsa05203	Viral carcinogenesis	Cancers: Overview
4790	NFKB1	hsa05212	Pancreatic cancer	Cancers: Specific types
4790	NFKB1	hsa05215	Prostate cancer	Cancers: Specific types
4790	NFKB1	hsa05220	Chronic myeloid leukemia	Cancers: Specific types
4790	NFKB1	hsa05221	Acute myeloid leukemia	Cancers: Specific types
5054	SERPINE1	hsa04066	HIF-1 signaling pathway	Signal transduction
5054	SERPINE1	hsa04115	p53 signaling pathway	Cell growth and death
5054	SERPINE1	hsa04933	AGE-RAGE signaling pathway in diabetic complications	Endocrine and metabolic diseases
5054	SERPINE1	hsa05142	Chagas disease (American trypanosomiasis)	Infectious diseases: Parasitic
5105	PCK1	hsa04068	FoxO signaling pathway	Signal transduction
5105	PCK1	hsa04151	PI3K-Akt signaling pathway	Signal transduction
51082	POLR1D	hsa05169	Epstein-Barr virus infection	Infectious diseases: Viral
5290	PIK3CA	hsa01521	EGFR tyrosine kinase inhibitor resistance	Drug resistance: Antineoplastic
5290	PIK3CA	hsa04014	Ras signaling pathway	Signal transduction
5290	PIK3CA	hsa04024	cAMP signaling pathway	Signal transduction
5290	PIK3CA	hsa04066	HIF-1 signaling pathway	Signal transduction
5290	PIK3CA	hsa04068	FoxO signaling pathway	Signal transduction
5290	PIK3CA	hsa04151	PI3K-Akt signaling pathway	Signal transduction
5290	PIK3CA	hsa04210	Apoptosis	Cell growth and death
5290	PIK3CA	hsa04211	Longevity regulating pathway	Aging
5290	PIK3CA	hsa04360	Axon guidance	Development
5290	PIK3CA	hsa04380	Osteoclast differentiation	Development

5290	PIK3CA	hsa04510	Focal adhesion	Cellular community
5290	PIK3CA	hsa04660	T cell receptor signaling pathway	Immune system
5290	PIK3CA	hsa04662	B cell receptor signaling pathway	Immune system
5290	PIK3CA	hsa04668	TNF signaling pathway	Signal transduction
5290	PIK3CA	hsa04722	Neurotrophin signaling pathway	Nervous system
5290	PIK3CA	hsa04915	Estrogen signaling pathway	Endocrine system
5290	PIK3CA	hsa04917	Prolactin signaling pathway	Endocrine system
5290	PIK3CA	hsa04932	Non-alcoholic fatty liver disease (NAFLD)	Endocrine and metabolic diseases
5290	PIK3CA	hsa04933	AGE-RAGE signaling pathway in diabetic complications	Endocrine and metabolic diseases
5290	PIK3CA	hsa05142	Chagas disease (American trypanosomiasis)	Infectious diseases: Parasitic
5290	PIK3CA	hsa05161	Hepatitis B	Infectious diseases: Viral
5290	PIK3CA	hsa05169	Epstein-Barr virus infection	Infectious diseases: Viral
5290	PIK3CA	hsa05200	Pathways in cancer	Cancers: Overview
5290	PIK3CA	hsa05203	Viral carcinogenesis	Cancers: Overview
5290	PIK3CA	hsa05210	Colorectal cancer	Cancers: Specific types
5290	PIK3CA	hsa05211	Renal cell carcinoma	Cancers: Specific types
5290	PIK3CA	hsa05212	Pancreatic cancer	Cancers: Specific types
5290	PIK3CA	hsa05215	Prostate cancer	Cancers: Specific types
5290	PIK3CA	hsa05220	Chronic myeloid leukemia	Cancers: Specific types
5290	PIK3CA	hsa05221	Acute myeloid leukemia	Cancers: Specific types
533	ATP6V0B	hsa05152	Tuberculosis	Infectious diseases: Bacterial
5364	PLXNB1	hsa04360	Axon guidance	Development
55048	VPS37C	hsa04144	Endocytosis	Transport and catabolism
554313	HIST2H4B	hsa05203	Viral carcinogenesis	Cancers: Overview
55660	PRPF40A	hsa03040	Spliceosome	Transcription
5602	MAPK10	hsa04010	MAPK signaling pathway	Signal transduction
5602	MAPK10	hsa04014	Ras signaling pathway	Signal transduction
5602	MAPK10	hsa04024	cAMP signaling pathway	Signal transduction
5602	MAPK10	hsa04068	FoxO signaling pathway	Signal transduction
5602	MAPK10	hsa04210	Apoptosis	Cell growth and death
5602	MAPK10	hsa04380	Osteoclast differentiation	Development

5602	MAPK10	hsa04510	Focal adhesion	Cellular community
5602	MAPK10	hsa04621	NOD-like receptor signaling pathway	Immune system
5602	MAPK10	hsa04668	TNF signaling pathway	Signal transduction
5602	MAPK10	hsa04722	Neurotrophin signaling pathway	Nervous system
5602	MAPK10	hsa04917	Prolactin signaling pathway	Endocrine system
5602	MAPK10	hsa04932	Non-alcoholic fatty liver disease (NAFLD)	Endocrine and metabolic diseases
5602	MAPK10	hsa04933	AGE-RAGE signaling pathway in diabetic complications	Endocrine and metabolic diseases
5602	MAPK10	hsa05132	Salmonella infection	Infectious diseases: Bacterial
5602	MAPK10	hsa05142	Chagas disease (American trypanosomiasis)	Infectious diseases: Parasitic
5602	MAPK10	hsa05152	Tuberculosis	Infectious diseases: Bacterial
5602	MAPK10	hsa05161	Hepatitis B	Infectious diseases: Viral
5602	MAPK10	hsa05169	Epstein-Barr virus infection	Infectious diseases: Viral
5602	MAPK10	hsa05200	Pathways in cancer	Cancers: Overview
5602	MAPK10	hsa05210	Colorectal cancer	Cancers: Specific types
5602	MAPK10	hsa05212	Pancreatic cancer	Cancers: Specific types
5710	PSMD4	hsa05169	Epstein-Barr virus infection	Infectious diseases: Viral
572	BAD	hsa01521	EGFR tyrosine kinase inhibitor resistance	Drug resistance: Antineoplastic
572	BAD	hsa04014	Ras signaling pathway	Signal transduction
572	BAD	hsa04024	cAMP signaling pathway	Signal transduction
572	BAD	hsa04151	PI3K-Akt signaling pathway	Signal transduction
572	BAD	hsa04210	Apoptosis	Cell growth and death
572	BAD	hsa04510	Focal adhesion	Cellular community
572	BAD	hsa04722	Neurotrophin signaling pathway	Nervous system
572	BAD	hsa05152	Tuberculosis	Infectious diseases: Bacterial
572	BAD	hsa05161	Hepatitis B	Infectious diseases: Viral
572	BAD	hsa05200	Pathways in cancer	Cancers: Overview
572	BAD	hsa05203	Viral carcinogenesis	Cancers: Overview
572	BAD	hsa05210	Colorectal cancer	Cancers: Specific types
572	BAD	hsa05212	Pancreatic cancer	Cancers: Specific types
572	BAD	hsa05215	Prostate cancer	Cancers: Specific types
572	BAD	hsa05220	Chronic myeloid leukemia	Cancers: Specific types

572	BAD	hsa05221	Acute myeloid leukemia	Cancers: Specific types
5728	PTEN	hsa01521	EGFR tyrosine kinase inhibitor resistance	Drug resistance: Antineoplastic
5728	PTEN	hsa04068	FoxO signaling pathway	Signal transduction
5728	PTEN	hsa04115	p53 signaling pathway	Cell growth and death
5728	PTEN	hsa04151	PI3K-Akt signaling pathway	Signal transduction
5728	PTEN	hsa04510	Focal adhesion	Cellular community
5728	PTEN	hsa05161	Hepatitis B	Infectious diseases: Viral
5728	PTEN	hsa05200	Pathways in cancer	Cancers: Overview
5728	PTEN	hsa05215	Prostate cancer	Cancers: Specific types
5783	PTPN13	hsa04210	Apoptosis	Cell growth and death
58498	MYL7	hsa04510	Focal adhesion	Cellular community
5879	RAC1	hsa04010	MAPK signaling pathway	Signal transduction
5879	RAC1	hsa04014	Ras signaling pathway	Signal transduction
5879	RAC1	hsa04024	cAMP signaling pathway	Signal transduction
5879	RAC1	hsa04151	PI3K-Akt signaling pathway	Signal transduction
5879	RAC1	hsa04360	Axon guidance	Development
5879	RAC1	hsa04380	Osteoclast differentiation	Development
5879	RAC1	hsa04510	Focal adhesion	Cellular community
5879	RAC1	hsa04520	Adherens junction	Cellular community
5879	RAC1	hsa04662	B cell receptor signaling pathway	Immune system
5879	RAC1	hsa04722	Neurotrophin signaling pathway	Nervous system
5879	RAC1	hsa04932	Non-alcoholic fatty liver disease (NAFLD)	Endocrine and metabolic diseases
5879	RAC1	hsa04933	AGE-RAGE signaling pathway in diabetic complications	Endocrine and metabolic diseases
5879	RAC1	hsa05132	Salmonella infection	Infectious diseases: Bacterial
5879	RAC1	hsa05200	Pathways in cancer	Cancers: Overview
5879	RAC1	hsa05203	Viral carcinogenesis	Cancers: Overview
5879	RAC1	hsa05210	Colorectal cancer	Cancers: Specific types
5879	RAC1	hsa05211	Renal cell carcinoma	Cancers: Specific types
5879	RAC1	hsa05212	Pancreatic cancer	Cancers: Specific types
5879	RAC1	hsa05416	Viral myocarditis	Cardiovascular diseases
5888	RAD51	hsa05200	Pathways in cancer	Cancers: Overview
5888	RAD51	hsa05212	Pancreatic cancer	Cancers: Specific types

5906	RAP1A	hsa04010	MAPK signaling pathway	Signal transduction
5906	RAP1A	hsa04014	Ras signaling pathway	Signal transduction
5906	RAP1A	hsa04024	cAMP signaling pathway	Signal transduction
5906	RAP1A	hsa04510	Focal adhesion	Cellular community
5906	RAP1A	hsa04722	Neurotrophin signaling pathway	Nervous system
5906	RAP1A	hsa05211	Renal cell carcinoma	Cancers: Specific types
5966	REL	hsa04014	Ras signaling pathway	Signal transduction
5966	REL	hsa05203	Viral carcinogenesis	Cancers: Overview
5970	RELA	hsa04010	MAPK signaling pathway	Signal transduction
5970	RELA	hsa04014	Ras signaling pathway	Signal transduction
5970	RELA	hsa04024	cAMP signaling pathway	Signal transduction
5970	RELA	hsa04066	HIF-1 signaling pathway	Signal transduction
5970	RELA	hsa04151	PI3K-Akt signaling pathway	Signal transduction
5970	RELA	hsa04210	Apoptosis	Cell growth and death
5970	RELA	hsa04211	Longevity regulating pathway	Aging
5970	RELA	hsa04380	Osteoclast differentiation	Development
5970	RELA	hsa04621	NOD-like receptor signaling pathway	Immune system
5970	RELA	hsa04660	T cell receptor signaling pathway	Immune system
5970	RELA	hsa04662	B cell receptor signaling pathway	Immune system
5970	RELA	hsa04668	TNF signaling pathway	Signal transduction
5970	RELA	hsa04722	Neurotrophin signaling pathway	Nervous system
5970	RELA	hsa04917	Prolactin signaling pathway	Endocrine system
5970	RELA	hsa04932	Non-alcoholic fatty liver disease (NAFLD)	Endocrine and metabolic diseases
5970	RELA	hsa04933	AGE-RAGE signaling pathway in diabetic complications	Endocrine and metabolic diseases
5970	RELA	hsa05132	Salmonella infection	Infectious diseases: Bacterial
5970	RELA	hsa05142	Chagas disease (American trypanosomiasis)	Infectious diseases: Parasitic
5970	RELA	hsa05152	Tuberculosis	Infectious diseases: Bacterial
5970	RELA	hsa05161	Hepatitis B	Infectious diseases: Viral
5970	RELA	hsa05169	Epstein-Barr virus infection	Infectious diseases: Viral
5970	RELA	hsa05200	Pathways in cancer	Cancers: Overview
5970	RELA	hsa05203	Viral carcinogenesis	Cancers: Overview

5970	RELA	hsa05212	Pancreatic cancer	Cancers: Specific types
5970	RELA	hsa05215	Prostate cancer	Cancers: Specific types
5970	RELA	hsa05220	Chronic myeloid leukemia	Cancers: Specific types
5970	RELA	hsa05221	Acute myeloid leukemia	Cancers: Specific types
64170	CARD9	hsa04621	NOD-like receptor signaling pathway	Immune system
64170	CARD9	hsa05152	Tuberculosis	Infectious diseases: Bacterial
6444	SGCD	hsa05416	Viral myocarditis	Cardiovascular diseases
6445	SGCG	hsa05416	Viral myocarditis	Cardiovascular diseases
64764	CREB3L2	hsa04024	cAMP signaling pathway	Signal transduction
64764	CREB3L2	hsa04151	PI3K-Akt signaling pathway	Signal transduction
64764	CREB3L2	hsa04211	Longevity regulating pathway	Aging
64764	CREB3L2	hsa04668	TNF signaling pathway	Signal transduction
64764	CREB3L2	hsa04915	Estrogen signaling pathway	Endocrine system
64764	CREB3L2	hsa05161	Hepatitis B	Infectious diseases: Viral
64764	CREB3L2	hsa05203	Viral carcinogenesis	Cancers: Overview
64764	CREB3L2	hsa05215	Prostate cancer	Cancers: Specific types
6625	SNRNP70	hsa03040	Spliceosome	Transcription
7040	TGFB1	hsa04010	MAPK signaling pathway	Signal transduction
7040	TGFB1	hsa04068	FoxO signaling pathway	Signal transduction
7040	TGFB1	hsa04144	Endocytosis	Transport and catabolism
7040	TGFB1	hsa04380	Osteoclast differentiation	Development
7040	TGFB1	hsa04932	Non-alcoholic fatty liver disease (NAFLD)	Endocrine and metabolic diseases
7040	TGFB1	hsa04933	AGE-RAGE signaling pathway in diabetic complications	Endocrine and metabolic diseases
7040	TGFB1	hsa05142	Chagas disease (American trypanosomiasis)	Infectious diseases: Parasitic
7040	TGFB1	hsa05152	Tuberculosis	Infectious diseases: Bacterial
7040	TGFB1	hsa05161	Hepatitis B	Infectious diseases: Viral
7040	TGFB1	hsa05200	Pathways in cancer	Cancers: Overview
7040	TGFB1	hsa05210	Colorectal cancer	Cancers: Specific types
7040	TGFB1	hsa05211	Renal cell carcinoma	Cancers: Specific types
7040	TGFB1	hsa05212	Pancreatic cancer	Cancers: Specific types
7040	TGFB1	hsa05220	Chronic myeloid leukemia	Cancers: Specific types

7046	TGFBR1	hsa04010	MAPK signaling pathway	Signal transduction
7046	TGFBR1	hsa04068	FoxO signaling pathway	Signal transduction
7046	TGFBR1	hsa04144	Endocytosis	Transport and catabolism
7046	TGFBR1	hsa04380	Osteoclast differentiation	Development
7046	TGFBR1	hsa04520	Adherens junction	Cellular community
7046	TGFBR1	hsa04933	AGE-RAGE signaling pathway in diabetic complications	Endocrine and metabolic diseases
7046	TGFBR1	hsa05142	Chagas disease (American trypanosomiasis)	Infectious diseases: Parasitic
7046	TGFBR1	hsa05161	Hepatitis B	Infectious diseases: Viral
7046	TGFBR1	hsa05200	Pathways in cancer	Cancers: Overview
7046	TGFBR1	hsa05210	Colorectal cancer	Cancers: Specific types
7046	TGFBR1	hsa05212	Pancreatic cancer	Cancers: Specific types
7046	TGFBR1	hsa05220	Chronic myeloid leukemia	Cancers: Specific types
7048	TGFBR2	hsa04010	MAPK signaling pathway	Signal transduction
7048	TGFBR2	hsa04068	FoxO signaling pathway	Signal transduction
7048	TGFBR2	hsa04144	Endocytosis	Transport and catabolism
7048	TGFBR2	hsa04380	Osteoclast differentiation	Development
7048	TGFBR2	hsa04520	Adherens junction	Cellular community
7048	TGFBR2	hsa04933	AGE-RAGE signaling pathway in diabetic complications	Endocrine and metabolic diseases
7048	TGFBR2	hsa05142	Chagas disease (American trypanosomiasis)	Infectious diseases: Parasitic
7048	TGFBR2	hsa05200	Pathways in cancer	Cancers: Overview
7048	TGFBR2	hsa05210	Colorectal cancer	Cancers: Specific types
7048	TGFBR2	hsa05212	Pancreatic cancer	Cancers: Specific types
7048	TGFBR2	hsa05220	Chronic myeloid leukemia	Cancers: Specific types
7251	TSG101	hsa04144	Endocytosis	Transport and catabolism
7431	VIM	hsa05169	Epstein-Barr virus infection	Infectious diseases: Viral
7532	YWHAG	hsa04151	PI3K-Akt signaling pathway	Signal transduction
7532	YWHAG	hsa05169	Epstein-Barr virus infection	Infectious diseases: Viral
7532	YWHAG	hsa05203	Viral carcinogenesis	Cancers: Overview
7534	YWHAZ	hsa04151	PI3K-Akt signaling pathway	Signal transduction

7534	YWHAZ	hsa05161	Hepatitis B	Infectious diseases: Viral
7534	YWHAZ	hsa05169	Epstein-Barr virus infection	Infectious diseases: Viral
7534	YWHAZ	hsa05203	Viral carcinogenesis	Cancers: Overview
7846	TUBA1A	hsa04210	Apoptosis	Cell growth and death
841	CASP8	hsa04115	p53 signaling pathway	Cell growth and death
841	CASP8	hsa04210	Apoptosis	Cell growth and death
841	CASP8	hsa04621	NOD-like receptor signaling pathway	Immune system
841	CASP8	hsa04668	TNF signaling pathway	Signal transduction
841	CASP8	hsa04932	Non-alcoholic fatty liver disease (NAFLD)	Endocrine and metabolic diseases
841	CASP8	hsa05142	Chagas disease (American trypanosomiasis)	Infectious diseases: Parasitic
841	CASP8	hsa05152	Tuberculosis	Infectious diseases: Bacterial
841	CASP8	hsa05161	Hepatitis B	Infectious diseases: Viral
841	CASP8	hsa05200	Pathways in cancer	Cancers: Overview
841	CASP8	hsa05203	Viral carcinogenesis	Cancers: Overview
841	CASP8	hsa05416	Viral myocarditis	Cardiovascular diseases
8482	SEMA7A	hsa04360	Axon guidance	Development
8569	MKNK1	hsa04010	MAPK signaling pathway	Signal transduction
8569	MKNK1	hsa04066	HIF-1 signaling pathway	Signal transduction
8737	RIPK1	hsa04210	Apoptosis	Cell growth and death
8737	RIPK1	hsa04668	TNF signaling pathway	Signal transduction
8737	RIPK1	hsa05169	Epstein-Barr virus infection	Infectious diseases: Viral
896	CCND3	hsa04115	p53 signaling pathway	Cell growth and death
896	CCND3	hsa04151	PI3K-Akt signaling pathway	Signal transduction
896	CCND3	hsa04510	Focal adhesion	Cellular community
896	CCND3	hsa05203	Viral carcinogenesis	Cancers: Overview
9064	MAP3K6	hsa04010	MAPK signaling pathway	Signal transduction
9370	ADIPOQ	hsa04211	Longevity regulating pathway	Aging
9370	ADIPOQ	hsa04932	Non-alcoholic fatty liver disease (NAFLD)	Endocrine and metabolic diseases
9530	BAG4	hsa04668	TNF signaling pathway	Signal transduction
9821	RB1CC1	hsa04211	Longevity regulating pathway	Aging
9879	DDX46	hsa03040	Spliceosome	Transcription
998	CDC42	hsa04010	MAPK signaling pathway	Signal transduction

998	CDC42	hsa04014	Ras signaling pathway	Signal transduction
998	CDC42	hsa04144	Endocytosis	Transport and catabolism
998	CDC42	hsa04360	Axon guidance	Development
998	CDC42	hsa04510	Focal adhesion	Cellular community
998	CDC42	hsa04520	Adherens junction	Cellular community
998	CDC42	hsa04660	T cell receptor signaling pathway	Immune system
998	CDC42	hsa04722	Neurotrophin signaling pathway	Nervous system
998	CDC42	hsa04932	Non-alcoholic fatty liver disease (NAFLD)	Endocrine and metabolic diseases
998	CDC42	hsa04933	AGE-RAGE signaling pathway in diabetic complications	Endocrine and metabolic diseases
998	CDC42	hsa05132	Salmonella infection	Infectious diseases: Bacterial
998	CDC42	hsa05200	Pathways in cancer	Cancers: Overview
998	CDC42	hsa05203	Viral carcinogenesis	Cancers: Overview
998	CDC42	hsa05211	Renal cell carcinoma	Cancers: Specific types
998	CDC42	hsa05212	Pancreatic cancer	Cancers: Specific types
